# A geometry- and muscle-based control architecture for synthesising biological movement

**DOI:** 10.1007/s00422-020-00856-4

**Published:** 2021-02-15

**Authors:** Johannes R. Walter, Michael Günther, Daniel F. B. Haeufle, Syn Schmitt

**Affiliations:** 1 Institute for Modelling and Simulation of Biomechanical Systems, Computational Biophysics and Biorobotics, University of Stuttgart, Nobelstraße 15, 70569 Stuttgart, Germany; 2 Center of Neurology, Hertie Institute for Clinical Brain Research, Otfried-Müller-Strasse 25, 72076 Tübingen, Germany; 3 Stuttgart Center of Simulation Science (SimTech), Pfaffenwaldring 7a, 70569 Stuttgart, Germany

**Keywords:** Biomechanics, Viscoelastic actuators, Motor control, Jacobian, PID controller, Upright stance, Squat movement, Morphological intelligence

## Abstract

A key problem for biological motor control is to establish a link between an idea of a movement and the generation of a set of muscle-stimulating signals that lead to the movement execution. The number of signals to generate is thereby larger than the body’s mechanical degrees of freedom in which the idea of the movement may be easily expressed, as the movement is actually executed in this space. A mathematical formulation that provides a solving link is presented in this paper in the form of a layered, hierarchical control architecture. It is meant to synthesise a wide range of complex three-dimensional muscle-driven movements. The control architecture consists of a ‘conceptional layer’, where the movement is planned, a ‘structural layer’, where the muscles are stimulated, and between both an additional ‘transformational layer’, where the muscle-joint redundancy is resolved. We demonstrate the operativeness by simulating human stance and squatting in a three-dimensional digital human model (DHM). The DHM considers 20 angular DoFs and 36 Hill-type muscle–tendon units (MTUs) and is exposed to gravity, while its feet contact the ground via reversible stick–slip interactions. The control architecture continuously stimulates all MTUs (‘structural layer’) based on a high-level, torque-based task formulation within its ‘conceptional layer’. Desired states of joint angles (postural plan) are fed to two mid-level joint controllers in the ‘transformational layer’. The ‘transformational layer’ communicates with the biophysical structures in the ‘structural layer’ by providing direct MTU stimulation contributions and further input signals for low-level MTU controllers. Thereby, the redundancy of the MTU stimulations with respect to the joint angles is resolved, i.e. a link between plan and execution is established, by exploiting some properties of the biophysical structures modelled. The resulting joint torques generated by the MTUs via their moment arms are fed back to the conceptional layer, closing the high-level control loop. Within our mathematical formulations of the Jacobian matrix-based layer transformations, we identify the crucial information for the redundancy solution to be the muscle moment arms, the stiffness relations of muscle and tendon tissue within the muscle model, and the length–stimulation relation of the muscle activation dynamics. The present control architecture allows the straightforward feeding of conceptional movement task formulations to MTUs. With this approach, the problem of movement planning is eased, as solely the mechanical system has to be considered in the conceptional plan.

**Supplementary Information:**

The online version supplementary material available at 10.1007/s00422-020-00856-4.

## Introduction

The desire to understand the principles of biological motion has a long history that spreads across many fields of research in science and robotics (Wiener 1948; Full and Koditschek 1999; Holmes et al. 2006; Pratt and Pratt 1998; Park 2001). One of the most challenging tasks hereby is to decipher the organisation of control within the nervous system, as among others approached by Kiehn (2016), Tresch et al. (1999), and Herzfeld and Shadmehr (2014). For low-level structures in the spinal cord, including $\alpha$- and $\gamma$-motor neurons, as well as for the muscles themselves, there is already strong biological evidence to have significant impact on movement control (Bizzi et al. 1992; van Soest and Bobbert 1993; Hulliger et al. 1989; Brändle et al. 2020). Neglecting those structures in computational modelling studies can even more lead to misleading conclusions (Pinter et al. 2012). In the scenario of complex movements, higher centres of the central nervous system (CNS) take an important role in planning and successful execution of a movement task (Martin 2005; Doya 2000; Gao et al. 1996). For successful execution, internal models of the body’s biophysical properties, including its neural wiring, and the external world, as needed, are present in any stable feedback control mechanism, be them explicitly learned or implicitly encoded in the nervous control system (Wolpert and Kawato 1998). A basic characteristic of biological structures is the redundancy of neural commands to realise the kinematics of one specific movement task in a space of fewer degrees of freedom (DoFs) (Wolpert 1997; Bernstein 1967). For this, selecting an appropriate combination of neural signals is traditionally perceived to be a tricky thing (Latash et al. 2002). It has been confirmed recently that a hierarchical organisation is beneficial in technical systems and the counterparts in biological hierarchy have been proposed (Merel et al. 2019).

**Fig. 1:**
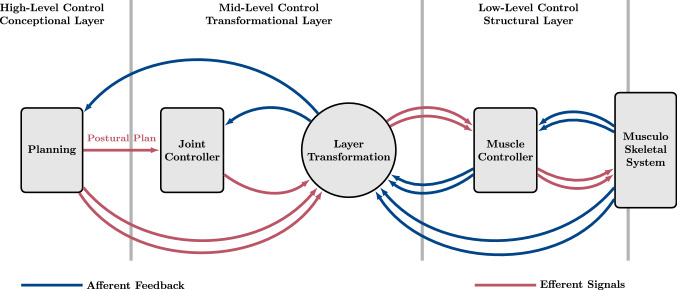
Schematic representation of the hierarchical control architecture. High-level conceptional planning in the control space of the task passes the postural plan to a mid-level Joint controller. The output of the joint controller is transformed to the control space of muscle variables and serves as input for the low-level structural muscle controller, which provides stimulation signals for the muscles (red lines). Proprioceptive sensors provide feedback signals, which are collected within the periphery and are passed to the hierarchically higher located control structures (blue lines). The process of planning and controlling hereby is easier in the control space of the task due to a smaller set of control variables. The dimensionality of the signals is illustrated by single (low-dimensional) and double (high-dimensional) arrows (colour figure online)

Similarly in this study, we present a hierarchical control architecture that is aimed towards full-scale body control. This control architecture is used in forward dynamics simulations to demonstrate that the biological feature of redundancy can be exploited for the generation of high-dimensional muscle stimulation signals in the lowest layer of the hierarchy. The movement plan is formulated in the top layer of the hierarchy in a specific low-dimensional coordinate space of an abstract task. The redundancy even opens a manifold of control signals to fulfil several criteria at once , for example, maintaining a posture with different levels of co-contraction. The conceptional planning in the task space has the advantage of a reduced processing effort due to a lower number of controlled state variables to consider and control parameters to deal with. The subsequent transformation of the task-fulfilling signals into the high-dimensional space of muscle stimulations is achieved by geometric transformations between task space coordinates (e.g. joint angles or torques) and actuator variables (e.g. muscle lengths or stimulations), as, for example, described by Pellionisz and Llinás (1985)^1^. That is, in the architecture suggested, these transformations resolve the redundancy by knowledge about the structural characteristics (e.g. anatomy). In the present work, all geometric transformations can even be expressed in closed form. With this, it becomes clear that some of the body’s properties, e.g. the muscle geometry and routing, are re-implemented in the suggested control architecture. An additional key property of this control architecture is the use of hierarchically distributed control laws in the conceptional, the transformational, and the structural layer (Fig. 1).

As an example, quiet upright stance is computer-synthesised. Human upright stance as a research topic has been widely studied in the fields of biomechanics and robotics (Rozendaal and van Soest 2005; Alexandrov et al. 2001; Edwards 2007; Günther and Wagner 2016; Pratt and Pratt 1998; Park 2001). As a preliminary step, thereby often the muscle contraction dynamics are neglected and the human movement system is idealised by its mechanical properties [as, for example, modelled solely by means of (1)]. This allows to propose control concepts which abstractly stabilise a multi-segment pendulum. One example is the control concept proposed by Günther and Wagner (2016). They suggested that at least a triple inverted pendulum with the open joints of ankle (An), knee (Kn), and hip (Hp) flexion–extension (FE) has to be considered to explain the natural oscillations. They (and others, e.g. Rozendaal and van Soest 2005; Alexandrov et al. 2001; Edwards 2007) suggested that a formulation of muscle-generated joint stiffnesses can lead to stable upright stance. This suggests a simple sensor-actor coupling similar to the idea of ‘impedance control’ (Hogan 1990) or ‘virtual model control’ (Henze 2002; Pratt et al. 1997). The (implicit) common ground that brings these works together is the idea of a decoupled planning and actuation frame [which is also hypothesised, for example, by Wolpert (1997)]: solving the mechanical system by calculating required torques must be followed (in the biological system) by muscle stimulations that eventually lead to said torques. This latter transformation is achieved by the here proposed hierarchical control architecture. It is a technical method that allows the straightforward execution of conceptional control hypotheses on muscle-actuated systems.

Concretely, in this contribution, the torque-based concept of Günther and Wagner (2016) is used for the synthesis of human upright stance. Their conceptional task formulation is based on joint torque–angle characteristics, which are expressed in terms of parameters like nominal angles, gains, and stiffnesses. Given a current posture, the joint torques advised by this task are the movement plan and used as desired input signals to the top-hierarchical controller in the conceptional layer. Its output is passed on to the transformational layer, where the plan is interpreted in terms of the postural plan (desired joint angles). In this layer, mid-level joint (angle) controllers and Jacobian-based layer transformations calculate desired muscle lengths and stimulation signals, that are fed to the structural layer. There, a low-level muscle (length) controller (Kistemaker et al. 2006; Bayer et al. 2017) that mimics the muscle spindle feedback loop generates a contribution to the stimulation signals applied to the modelled muscles. The torques caused by the generated muscle forces are fed back to the top-hierarchical conceptional controller, which closes the control loop.

This paper introduces the suggested hierarchical control architecture and demonstrates its performance, robustness, and potentially versatile operativeness by not only synthesising human stance (also perturbed by noise) but also a goal-directed movement, namely, squatting and rising again. Some degree of robustness can be inferred from the sheer fact that the whole set of parameters required by the architecture could be set heuristically manually.

## Model and control framework

To perform forward dynamics simulations of biophysical systems, mathematical models are required as a basis for algorithmic implementations. For this, it is not only the mathematical models of certain subsystems that are of interest but also the exchange of information between those subsystems. In computational modelling, exchange of information thereby describes the communication of the subsystems and represents biophysical processes, such as neural transmission of electrical signals along axons and transduction of muscle forces along tendons to bones. The control structure, in particular, forms a closed loop feedback unit, which interacts with the biomechanical structures of the body through sensor signals and muscle stimulations. Therefore, it is conceivable that, to produce coordinated movements, the controller benefits of having embedded some information about the body’s peripheral structures and the properties of the muscles. In the approach presented here, it is suggested that the geometry of the body and its joints, the routing of the muscles, and the dynamics of the muscles themselves are essential structural properties to be taken into account. In Sect. 2.1, these properties are introduced and then merged into the hierarchical control architecture as explained in Sect. 2.2.

### The musculo-skeletal model (plant)

*(a) The rigid-body model.* Rigid-body dynamics are used to simulate three-dimensional movements of the skeletal system. In this, a chain of rigid bodies, connected by rotational joints, is modelled by means of differential equations. The whole-body movement is described by six DoFs (each three linear and angular ones) of a reference body plus the ${n_\theta }$ DoFs of the rotational joints ${\varvec{\theta }}=[\theta _1,\,\dots ,\,\theta _{{n_\theta }}]^T\in {\mathbb {R}}^{{n_\theta }}$ that are actuated by muscles, and the ${\tilde{n}}_\theta$ rotational DoFs ${\tilde{{\varvec{\theta }}}}=[{\tilde{\theta }}_1,\,\dots ,{\tilde{\theta }}_{{\tilde{n}}_\theta }]^T\in {\mathbb {R}}^{{\tilde{n}}_\theta }$ that are not. That is, the vector of generalised coordinates

$$\begin{aligned} {\varvec{q}}=[{q}_1,\,\dots ,\,{q}_6,\,\theta _1,\,\dots ,\theta _{n_\theta },\, {\tilde{\theta }}_1,\,\dots ,{\tilde{\theta }}_{{\tilde{n}}_\theta }]^T\in {\mathbb {R}}^{{n_\text {DoF}}} \end{aligned}$$

contains a total number of ${n_\text {DoF}}=6+{n_\theta }+{\tilde{n}}_\theta$ mechanical DoFs. With these generalised coordinates ${{\varvec{q}}}$ being state variables, a Lagrangian formulation of the body’s equations of motion

1 $$\begin{aligned} {{M}}({{\varvec{q}}})\ddot{{{\varvec{q}}}}+{{\varvec{c}}}({{\varvec{q}}},\dot{{{\varvec{q}}}}) ={{{{\varvec{T}}}}}, \end{aligned}$$

can be set up algorithmically, where ${{M}}\in {\mathbb {R}}^{{{n}_\text {DoF}}\times {{n}_\text {DoF}}}$ is the mass matrix, ${{\varvec{c}}}\in {\mathbb {R}}^{{{n}_\text {DoF}}}$ is a vector of gravitational, centrifugal, and Coriolis forces, and ${{\varvec{T}}} ={[}{{{T}}}_{1} ,\ldots ,\, {{{{T}}}}_6,\,\tau _1,\ldots ,\tau _{{{n_\theta }}}, \,{\tilde{\tau }}_1,\ldots ,\,{\tilde{\tau }}_{{{\tilde{n}}_\theta }}]^T \in {\mathbb {R}}^{n}_{\mathrm{DoF}}$ is a vector of generalised forces, with ${\varvec{\tau }}=[\tau _1,\,\dots ,\,\tau _{{{n_\theta }}}]^T\in {\mathbb {R}}^{{{n_\theta }}}$ being the vector of generalised joint forces (joint torques)

2 $$\begin{aligned} {\varvec{\tau }}={\varvec{\tau }}^\text {MTU}+{\varvec{\tau }}^{\text {lmt}}+{\varvec{\tau }}^{\text {bsh}} \end{aligned}$$

of the body’s joints actuated by muscle–tendon units (MTUs). Equation (1) is implemented using the formulation described by Legnani et al. (1996a, 1996b). The vector ${{{{\varvec{T}}}}}$ includes forces due to the body contacting the ground (${{{{\varvec{T}}}}}^\text {cnt}$), as well as forces by muscles (${\varvec{\tau }}^\text {MTU}$), joint limitations (${\varvec{\tau }}^{\text {lmt}}$), and viscoelastic rotational springs (${\varvec{\tau }}^{\text {bsh}}$). Details on the specific force laws used, and their local incorporation into the model, are given in Appendix A, as well as in the *supplementary material*.

**Fig. 2:**
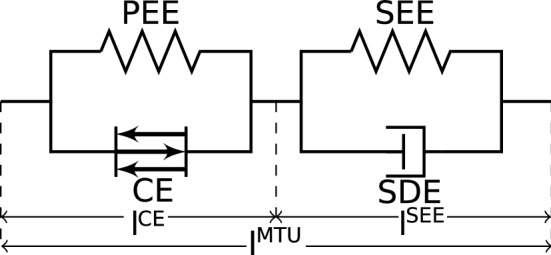
Diagram of a muscle–tendon unit (MTU) with contractile element (CE), parallel elastic element (PEE), serial elastic element (SEE), and serial damping element (SDE) (Haeufle et al. 2014a)

*(b) The extended Hill-type muscle model.* The muscle model used in this study describes each muscle as a deflected, one-dimensional, massless, string-like muscle–tendon unit (MTU), in detail outlined in Haeufle et al. (2014a). It consists of an active contractile element (CE), a passive parallel elastic element (PEE), a serial elastic element (SEE), and a serial damping element (SDE) (Fig. 2). The CE and the PEE represent the properties of the fibre material in the muscle belly, SEE and SDE those of the aponeuroses and the tendons. The total length $l^\text {MTU}_k$ of the *k*-th muscle is composed of the CE length $l^{\mathrm{CE}}_k$ and the SEE length $l^{\mathrm{SEE}}_k$:

3 $$\begin{aligned} l^\text {MTU}_k=l^{\mathrm{CE}}_k+l^{\mathrm{SEE}}_k. \end{aligned}$$

The resulting force $f^\text {MTU}_k$ of the *k*-th MTU is then given by the force equilibrium of the forces of the CE, the PEE, the SEE, and the SDE:

4 $$\begin{aligned} f^\text {MTU}_k=f^{\mathrm{CE}}_k+f^{\mathrm{PEE}}_k=f^{\mathrm{SEE}}_k+f^\text {SDE}_k. \end{aligned}$$

Here, each force element is described by nonlinear functions. A synopsis of the force characteristics of the MTU elements is given in Appendix A.1.

Solving the right-hand equality of (4) for the CE’s contraction velocity ${\dot{l}}^{\mathrm{CE}}_k$ provides the contraction dynamics of the *k*-th CE:

5 $$\begin{aligned} {\dot{l}}^{\mathrm{CE}}_k={\dot{l}}^{\mathrm{CE}}_k(l^{\mathrm{CE}}_k, l^\text {MTU}_k, {\dot{l}}^{\mathrm{MTU}}_k, a_k), \end{aligned}$$

with $a_k(t)\in {\mathbb {R}}_{01}$ being the CE’s activity (see (7) below) and ${\mathbb {R}}_{01}$ the set of real numbers ${\mathbb {R}}$ bounded by the interval $[0\;1]$. When a neural spike is received by a muscle fibre at its neuromuscular junction, a biochemical process is initiated that leads to the release of calcium ions and a subsequent fibre twitch. The response of the normalised concentration $\gamma _k(t)\in {\mathbb {R}}_{01}$ of free $\text {Ca}^{2+}$-ions in the sarcoplasm to a train of neural spikes (tetanus) is modelled as the CE’s activation dynamics

6 $$\begin{aligned} {\dot{\gamma }}_k(t)=f_{\gamma ,k}(\gamma _k(t), u_k(t)), \end{aligned}$$

in which the neural excitation that reaches the *k*-th MTU is a continuous stimulation signal $u_k(t)\in {\mathbb {R}}_{01}$. The subsequent biochemical processes to the point of steady-state cross-bridge force generation are implemented in the form of a sigmoidal (activity) function (Rockenfeller and Günther 2017):

7 $$\begin{aligned} a_k(t)=f_{a,k}(\gamma _k(t), l^{\mathrm{CE}}_k(t)). \end{aligned}$$

Equations (6) and (7) taken together are Hatze’s activation dynamics (Hatze 1977; Rockenfeller et al. 2015), which are implemented here in a polished version (Rockenfeller and Günther 2018). The system of coupled contraction (5) and activation (6) dynamics is numerically integrated over time during a body movement to provide the state variables CE length $l^{\mathrm{CE}}_k(t)$ and ion concentration $\gamma _k(t)$ of the *k*-th MTU. The consequential state of activity $a_k(t)$ from (7) represents the source of mechanical energy provided by ATP hydrolysis. Activity causes a contractile force $f^{\mathrm{CE}}_k(l^{\mathrm{CE}}_k, {\dot{l}}^{\mathrm{CE}}_k, a_k)$ of the CE, which eventually determines MTU force $f^\text {MTU}_k(l^{\mathrm{CE}}_k, {\dot{l}}^{\mathrm{CE}}_k, a_k)$ at any point in time by summing up $f^{\mathrm{CE}}_k$ and $f^{\mathrm{PEE}}_k$ with knowing the state of the mechanical system (1) and the MTU (5, 6) [left-hand equality in (4)]. Note that ${\dot{l}}^{\mathrm{CE}}_k$ depends on $l^\text {MTU}_k$, ${\dot{l}}^{\mathrm{MTU}}_k$, $l^{\mathrm{CE}}_k$, and $a_k$ [see (5)], as does $a_k$ on $l^{\mathrm{CE}}_k$ and $\gamma _k$ [see (7)]. Thus, both $f^{\mathrm{CE}}_k$ and $f^\text {MTU}_k$ inherit these dependencies. The whole process of MTU force production is described at full length by Haeufle et al. (2014a).

**Fig. 3:**
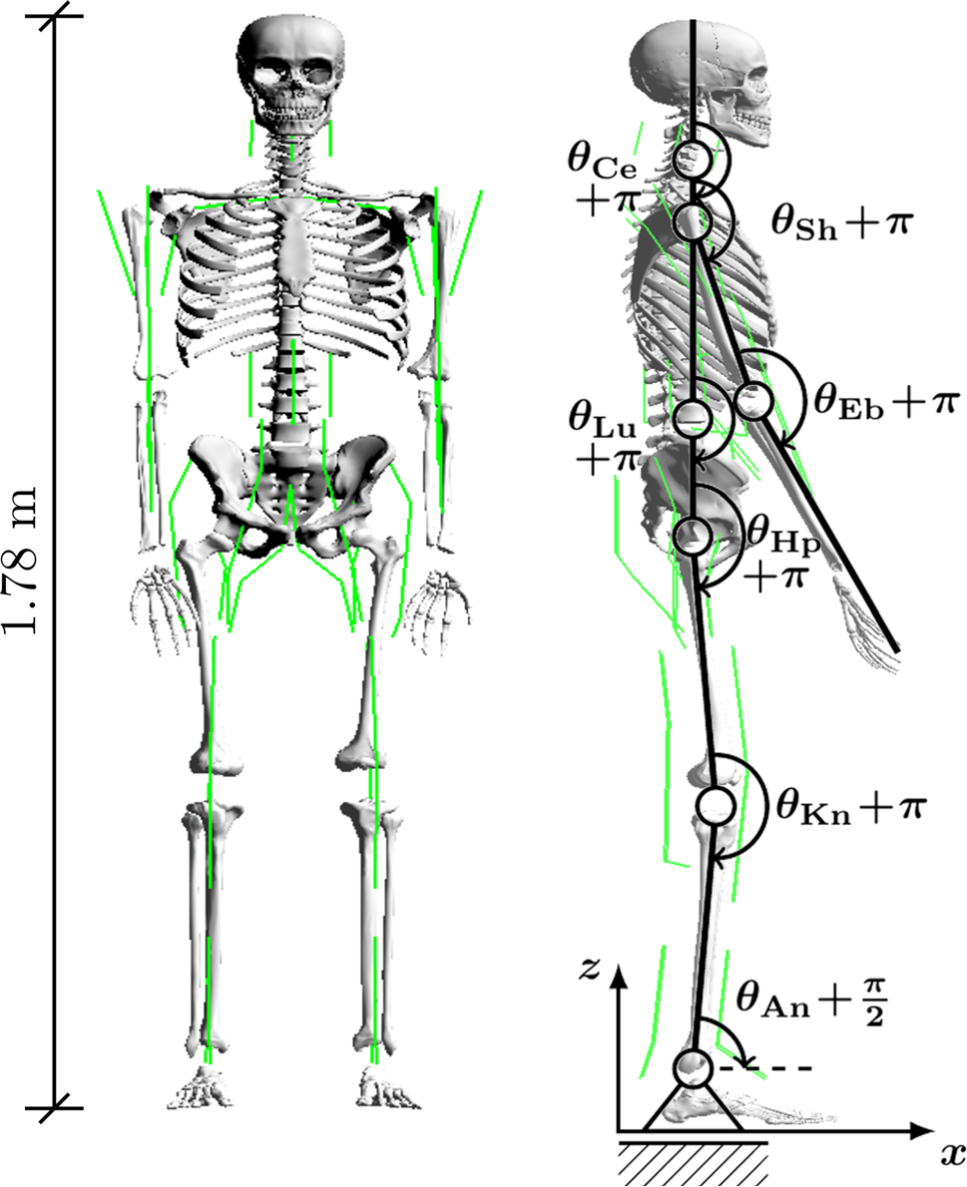
The digital human model (DHM) used in this study is a simplified representation of the human body. The DHM consists of a chain of 15 rigid bodies (segments), which are connected by joints that allow movements in $n_\text {DoF}=20$ angular degrees of freedom (DoFs). The joints are actuated by ${n_\text {MTU}}=36$ string-like, massless Hill-type muscle–tendon units (MTUs) (Haeufle et al. 2014a) (see Sect. 2.1b). With some exceptions, MTUs are routed by via-points. The wrist joints are stabilised by passive spring like elements, rather than actuated by MTUs. Nonlinear viscoelastic force elements, allowing reversible stick–slip transitions, model ground-feet contact interactions (Appendix A.4). For further details on the parameter values of the DHM, refer to the *supplementary material*

The MTU string’s deflection by anatomical structures is achieved with the ‘via-ellipse’ method (Hammer et al. 2019). This deflection method leads to nonlinear joint angle-dependent moment arms of the MTUs. The resulting generalised force $\tau ^\text {MTU}_{j,k}$, generated by the *k*-th MTU, acting in the direction of the *j*-th degree of freedom is $\tau ^\text {MTU}_{j,k}= -{r}_{j,k}({\varvec{\theta }})f^\text {MTU}_k$, where ${r}_{j,k}({\varvec{q}})$ denotes the joint angle-dependent moment arm of the *k*-th muscle passing the *j*-th joint. This moment arm can be visualised geometrically as the perpendicular distance of the muscle string’s line of action from the joint DoF’s centre of rotation. For a multi-joint, multi-muscle system with the vector of MTU lengths ${\varvec{l}}^{\mathrm{MTU}}=\left[ l^\text {MTU}_1,\, \ldots ,\, l^\text {MTU}_{{n_\text {MTU}}}\right] ^T\in {\mathbb {R}}^{{n_\text {MTU}}}$ and the vector of MTU forces ${\varvec{f}}^\text {MTU}=\left[ f^\text {MTU}_1,\, \ldots ,\, f^\text {MTU}_{{n_\text {MTU}}}\right] ^T\in {\mathbb {R}}^{{n_\text {MTU}}}$, the Jacobi-like matrix of moment arms

8 $$\begin{aligned} {{R}}({\varvec{\theta }})=\frac{\partial {\varvec{l}}^{\mathrm{MTU}}}{\partial {\varvec{\theta }}} \in {\mathbb {R}}^{{n_\text {DoF}}\times {n_\text {MTU}}} \end{aligned}$$

can be defined, and the vector of generalised muscle forces ${\varvec{\tau }}^\text {MTU}=\left[ \tau ^\text {MTU}_1,\, \ldots ,\, \tau ^\text {MTU}_{{n_\text {MTU}}}\right] ^T\in {\mathbb {R}}^{{n_\text {MTU}}}$, acting on the skeletal system, is given by

9 $$\begin{aligned} {\varvec{\tau }}^\text {MTU}=-{{R}}^T({\varvec{\theta }}){\varvec{f}}^\text {MTU}. \end{aligned}$$

Here, the negative sign is chosen in accordance to Stanev and Moustakas (2019). Though, it must be emphasised that a set of conventions for the physical variables involved ($l^\text {MTU}$, $\theta$, $f^\text {MTU}$ and $\tau ^\text {MTU}$), and how they are used in the mechanical equations of motion, determine what sign each component of the Jacobian ${{R}}({\varvec{\theta }})$ eventually carries.

*(c) The digital human model (DHM).* The DHM used in this study is shown in Fig. 3 and is in detail explained in the *supplementary material*. The pelvis body is the centre of the ramified rigid-body chain. In the neutral, upright posture of the DHM, as depicted in Fig. 3, due to hinge joint axes aligned in parallel, active FE movements within the sagittal plane can be generated in the e lumbar joint (Lu), the cervical joint (Ce), and each left and right Hp, Kn, An, shoulder (Sh), and elbow (Eb). Additionally, due to perpendicularly oriented hinge joint axes, AA movements within the coronal plane can occur in the Lu, the Ce, the Hps and the Shs. The muscles are lumped (mono-articular) constructs, implementing the concept of an ‘elementary biological drive’ (Schmitt et al. 2019) for joint actuation: for each hinge joint, there is exactly one flexor (Fx) and one extensor (Ex) muscle.

### The hierarchical control architecture

As biological movement is driven by stimulated muscles, both the movement and the goal of the task are implicitly present in the coordinate frame of muscle stimulations, which encode the neural input to the musculo-skeletal model. This does not imply, though, that the whole movement is planned in terms of muscle stimulation coordinates, and it is worth considering that planning may be done significantly easier in terms of a more suitable set of coordinates.

Picking up this consideration and applying it in a straightforward way, in this section, an essentially plain concept of planning and then generating coordinated movements is laid out: The MTUs actuating the joint DoFs ${\varvec{\theta }}$ are stimulated by signals ${\varvec{u}}(t)=[u_1,\,\ldots ,\,u_{{n_\text {MTU}}}]^T\in {\mathbb {R}}_{01}^{{n_\text {MTU}}}$, which lead to MTU forces ${\varvec{f}}^\text {MTU}(t)\in {\mathbb {R}}^{{n_\text {MTU}}}$ and, thus, joint torques ${\varvec{\tau }}^\text {MTU}(t)\in {\mathbb {R}}^{{n_\text {DoF}}}$ that drive the current joint state (posture) ${\varvec{\theta }}(t)$ of the movement system to the desired state ${\varvec{\theta }}^\text {des}(t)=[\theta ^\text {des}_1,\,\ldots ,\,\theta ^\text {des}_{{n_\theta }}]^T\in {\mathbb {R}}^{{n_\theta }}$.

**Fig. 4:**
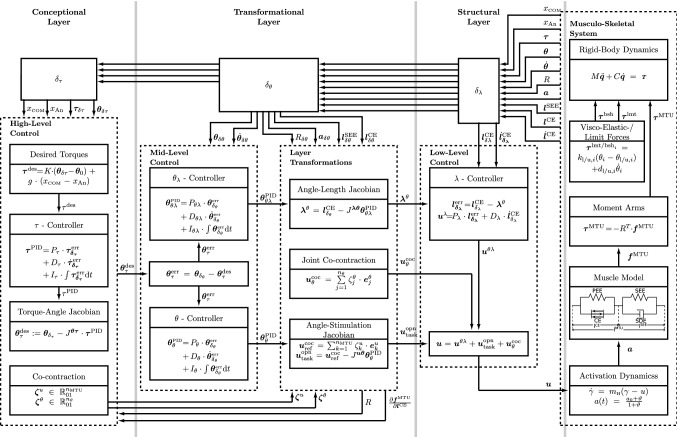
Complete schematics of the presented feedback control architecture. In the conceptional layer, a desired joint angle configuration ${\varvec{\theta }}^\text {des}$ (postural plan) is calculated for a specific task that is formulated as a torque-based concept. Comparing ${\varvec{\theta }}^\text {des}$ to the joint angle configuration ${\varvec{\theta }}_{\delta _\theta }$, the resulting control error ${\varvec{\theta }}^\text {err}$ serves as input for the PID controllers in the transformational layer, i.e. for the direct $\theta$-controller and the hierarchical $\theta _\lambda$-controller. The subsequent controller outputs are transformed to the structural layer by Jacobian matrices (layer transformations). The output of the $\theta _\lambda$-controller is passed to the low-level $\lambda$-controller, which produces the hierarchically controlled stimulation signal ${\varvec{u}}^{\theta \lambda }$ (23). The co-contraction stimulations ${\varvec{u}}^\text {coc}_\theta$ (36) and ${\varvec{u}}^\text {coc}_\text {ref}$ (33) plus the $\theta$-controlled stimulation ${\varvec{u}}^\theta$ (30) provide a direct contribution to the MTUs stimulation, as proposed by the hybrid control ansatz (Bayer et al. 2017), there labelled ‘open loop’. The two addends ${\varvec{u}}^\text {coc}_\text {ref}$ and ${\varvec{u}}^\theta$ are stimulation signals complementary for fulfilling the task: ${\varvec{u}}^\text {coc}_\text {ref}$ is an arbitrary base contraction level, and ${\varvec{u}}^\theta$ minimises the difference between the base ${\varvec{u}}^\text {coc}_\text {ref}$ and the desired stimulation value ${\varvec{u}}^\text {opn}_\text {task}$ that corresponds to ${\varvec{\theta }}^\text {des}$ and constitutes the plan. To fulfil movement criteria along with the desired task, ${\varvec{u}}^\text {coc}_\theta$ can adjust the MTU co-contractions at each joint by utilising the null-space (uncontrolled manifold) of (the pseudo-inverse of) ${J}^{{\varvec{u}}{\varvec{\theta }}}$. As a specific example, the torque concept delivering the postural plan ${\varvec{\theta }}^\text {des}$ deploys, in this study, angle–torque characteristics that stabilise upright stance (Günther and Wagner 2016) (see Sect. 3.1). Yet, the present hierarchical control architecture generally invites deploying arbitrary torque-based concepts

This concept requires drafting a desired state ${\varvec{\theta }}^\text {des}$. In the hierarchical control architecture proposed here (Fig. 1), ${\varvec{\theta }}^\text {des}$ is drafted in a planning process in the conceptional layer (high-level control). Depending on a specific movement task, many planning strategies and mechanisms in various frames of coordinates may have the potential of successful execution. For the exemplary movement task of human upright stance examined here, the desired state ${\varvec{\theta }}^\text {des}$ is obtained (see Sect. 2.2.3) from a characteristic relation between joint torques ${\varvec{\tau }}$, which are interpreted as desired torques ${\varvec{\tau }}^\text {des}$, and joint angles ${\varvec{\theta }}$, which represent the current mechanical state. The postural movement plan ${\varvec{\theta }}^\text {des}$ obtained this way is then used as input for a joint controller in the transformational layer (mid-level control). A subsequent transformation to the structural layer (low-level control) provides muscle stimulations that cause the MTU contributions ${\varvec{\tau }}^\text {MTU}$ to the joint torques to fulfil the task by continuously letting ${\varvec{\tau }}^\text {MTU}$ approach ${\varvec{\tau }}^\text {des}$ (symbolised by ${\varvec{\tau }}^\text {MTU}\rightarrow {\varvec{\tau }}^\text {des}$). Within its layers—the conceptional, the transformational, and the structural (Fig 1)—the proposed control architecture implements the planning and generating concept by combining ordinary proportional–integral–derivative (PID) feedback control laws with Jacobian matrices that transform from the set of controlled physical variables on the layer input side to another set on the output side (see Fig. 4).

The task planning in the conceptional layer of joint torques is eased because solely the state of the mechanical system (1) has to be considered, that is, the dimension of the planning space is of lower dimension ($\le 2\cdot {n_\text {DoF}}$) than the state space of the whole movement system ($\ge 2\cdot {n_\text {MTU}}+2\cdot {n_\text {DoF}}$), and nothing has to be known about the properties of the biological structures. However, in order to execute the planned control scheme, the remainder of the proposed control architecture then heavily relies on structural knowledge, as any Jacobian introduced contains such. This can be seen as a general principle of resolving redundancy. As the so introduced Jacobians are generally non-square, with the transformation’s image space being of the higher dimension, the non-uniqueness of the solution in the image space [null-space or ‘uncontrolled manifold’ (Scholz and Schöner 1999)] opens freedom for control, as it allows a manifold of movement solutions executing the same task, e.g. with selectable degrees of joint co-contractions. The structural knowledge represented by the Jacobians is such about the muscle’s routing (moment arms), the stiffnesses due to passive and active muscle tissue properties (contraction dynamics), and the characteristics of the activation dynamics.

In the following, the constituents of the three layers of the control architecture, that is, the four controllers, the three Jacobian-based transformations, and the fixation of co-contraction parameter values, are explained in detail.

#### Low-level control in the structural layer

The structural layer is perceived here as an ${n_\text {MTU}}$-dimensional space, which contains the representations of the MTUs and associated biological structures (e.g. muscle spindles). Biophysical control-related signals within this layer include the vector of muscle stimulation signals ${\varvec{u}}\in {\mathbb {R}}^{{n_\text {MTU}}}_{01}$, which is the layer’s output that serves as neural input to the muscles’ activation dynamics (6), and the vector of CE lengths ${\varvec{l}}^{\mathrm{CE}}=\left[ l^{\mathrm{CE}}_1,\, \ldots ,\, l^{\mathrm{CE}}_{{n_\text {MTU}}}\right] ^T\in {\mathbb {R}}^{{n_\text {MTU}}}$ being the state variables of the muscles’ contraction dynamics (5).

In this structural layer, for each MTU, a mathematical representation of a low-level feedback control mechanism on the spinal level is implemented, constituting the initial nucleus and pivot of the here presented hierarchical control architecture. The structural embodiment of this feedback control mechanism is the mono-synaptic spinal cord reflex arc that transmits muscle spindle signals from the intrafusal muscle fibres via a pool of $\alpha$-motoneurons to the extrafusal fibres. A most simple implementation of this spindle-based feedback control mechanism is a simple P-controller comparison of the actual and desired muscle fibre length (in the literature, for example, associated with $\lambda$-controller) (McIntyre and Bizzi 1993). This simple approach has proven to be a practicable approach for the forward-dynamics synthesis of biological movements (Günther and Ruder 2003), especially as part of a ‘hybrid controller’, i.e. combined with a feedforward term (Kistemaker et al. 2006; Bayer et al. 2017; Stollenmaier et al. 2020). It is here implemented in the form of a proportional–derivative (PD) controller, taking respect of the potential contraction–velocity-related spindle-feedback signal. The symbol $\lambda$ represents the length threshold of a muscle’s stretch reflex (Feldman 1974), which can be adjusted by the excitation (and inhibition) of the intrafusal fibres via the $\gamma$-motoneurons (Matthews 1959). This is reflected in Fig. 4 by the arrow labelled with ${\varvec{\lambda }}^\theta$, which points from the transformational to the structural layer.

Using the vectors of actual and desired CE lengths, ${\varvec{l}}^{\mathrm{CE}}\in {\mathbb {R}}^{{n_\text {MTU}}}$ and ${\varvec{\lambda }}=\left[ \lambda _1,\, \ldots ,\, \lambda _{{n_\text {MTU}}}\right] ^T\in {\mathbb {R}}^{{n_\text {MTU}}}$, respectively, the control error on the structural layer, with the neural pathway latency times ${\varvec{\delta _\lambda }}=\left[ \delta _{\lambda ,1},\, \ldots ,\, \delta _{\lambda ,{n_\text {MTU}}}\right] ^T\in {\mathbb {R}}^{{n_\text {MTU}}}$, is given by

10 $$\begin{aligned} {\varvec{l}}^\text {err}_{{\varvec{\delta _\lambda }}}(t):={\varvec{l}}^{\mathrm{CE}}(t,{\varvec{\delta _\lambda }})-{\varvec{\lambda }}(t), \end{aligned}$$

with ${\varvec{l}}^{\mathrm{CE}}(t,{\varvec{\delta _\lambda }}):=\left[ l^{\mathrm{CE}}_1(t-\delta _{\lambda ,1}),\,\ldots ,\,l^{\mathrm{CE}}_{{n_\text {MTU}}} (t-\delta _{\lambda ,{n_\text {MTU}}})\right] ^T$ being the vector of CE lengths, delayed by their respective individual latency times. The $\lambda$-controller is then used to generate the stimulation signals ${\varvec{u}}^\lambda =\left[ u^\lambda _{1},\, \ldots ,\, u^\lambda _{{n_\text {MTU}}}\right] ^T\in {\mathbb {R}}^{n_\text {MTU}}$ by

11 $$\begin{aligned} {\varvec{u}}^\lambda (t)={P}_\lambda \cdot {\varvec{l}}^\text {err}_{{\varvec{\delta _\lambda }}}(t)+{D}_\lambda \cdot \dot{{\varvec{l}}}^\text {err}_{{\varvec{\delta _\lambda }}}(t), \end{aligned}$$

where the matrices ${P}_\lambda =\text {diag}(p_{\lambda ,1},\,\ldots \,p_{\lambda ,{n_\text {MTU}}})\in {\mathbb {R}}^{{n_\text {MTU}}\times {n_\text {MTU}}}$ and ${D}_\lambda =\text {diag}(d_{\lambda ,1},\,\ldots \,d_{\lambda ,{n_\text {MTU}}})\in {\mathbb {R}}^{{n_\text {MTU}}\times {n_\text {MTU}}}$ are diagonal matrices, containing the control parameters $p_{\lambda ,i}>0$ and $d_{\lambda ,i}>0$, respectively.

This closed-loop feedback controller can be sufficient for the generation of asymptotically stable postures even in the presence of an external force (e.g. gravity) to which a remaining constant control deviation corresponds. This can be clearly seen, as $u^\lambda _{k}\rightarrow 0$ for $l^{\mathrm{CE}}_k\rightarrow \lambda _k$, which yields $f^{\mathrm{CE}}_k\rightarrow 0$. For still reaching a desired posture, i.e. compensating the control deviation, an additional ‘open-loop’ (from the perspective of the structural layer) stimulation signal ${\varvec{u}}^\text {opn}(t)=\left[ u^\text {opn}_{1},\, \ldots ,\, u^\text {opn}_{{n_\text {MTU}}}\right] ^T\in {\mathbb {R}}^{{n_\text {MTU}}}$ can be deployed. Such compensating signals may be provided in a biological system via the pool of $\alpha$-motoneurons adding contributions to the MTU stimulations that are based on memory or state knowledge from outside the structural layer. This is reflected in Fig. 4 by the bottom summation box in structural layer (see also Sect. 2.2.2).

The combination of closed-loop $\lambda$-control stimulation ${\varvec{u}}^\lambda$ according to (11) and open-loop stimulation ${\varvec{u}}^\text {opn}$ has been shown to be capable of generating simple coordinated movements and is also termed ‘hybrid controller’ of muscle-based systems (Bayer et al. 2017; Kistemaker et al. 2006):

12 $$\begin{aligned} {\varvec{u}}(t)={\varvec{u}}^\lambda (t)+{\varvec{u}}^\text {opn}(t). \end{aligned}$$

However, for the generation of complex movements, finding suitable inputs ${\varvec{\lambda }}\in {\mathbb {R}}^{{n_\text {MTU}}}$ and ${\varvec{u}}^\text {opn}\in {\mathbb {R}}^{{n_\text {MTU}}}$ for a stand-alone hybrid controller will result complicated. For a system of ${n_\text {MTU}}$ muscles, a total of $2\cdot {n_\text {MTU}}$ control inputs are needed. Furthermore, due to the nonlinear muscle properties and their redundant embodiment, the controller inputs cannot be chosen arbitrarily, as they are constrained, e.g. due to muscles acting on the same joint requiring synergistic action. Given the hybrid controller constitutes the core of a low-level structural layer, the redundancy of the plant has therefore to be resolved in order to set a well-tuned input for the hybrid controller.

#### Mid-level control in the transformational layer

A control architecture in the transformational (joint) layer can help in resolving the redundancy problem for which the simplest description may be that there is a greater number of ${n_\text {MTU}}$ muscles acting on a smaller set of ${n_\theta }$ joints. As already mentioned above, there are two benefits of this: one for movement planning and a second for movement control.

Regarding the first, the space for planning is of lower dimension (${n_\theta }$ desired joint angles) than the actuator space (${n_\text {MTU}}$ MTUs). Regarding the second, this opens a manifold of dimension $({n_\text {MTU}}-{n_\theta })$ to satisfy additional criteria (see in detail Sect. 2.2.2d) during the controlled movement, concisely symbolised by $\theta \rightarrow \theta ^\text {des}$.

In the following, the two controllers in the transformational layer and their respective Jacobian-based transformations are presented. Exactly these Jacobians solve the joint-muscle redundancy.

The first of the two (mid-level) controllers in this transformational layer is termed $\theta _\lambda$-controller (for details, see Sect. 2.2.2a). It takes the desired state in terms of joint angles ${\varvec{\theta }}^\text {des}\in {\mathbb {R}}^{{n_\theta }}$ and uses the angle–length Jacobian ${J}^{{\varvec{\lambda }}{\varvec{\theta }}}\in {\mathbb {R}}^{{n_\text {MTU}}\times {n_\theta }}$, which contains structural knowledge about muscle moment arms and MTU-internal stiffness ratios, to transmit values of desired muscle lengths ${\varvec{\lambda }}^\theta (t)=\left[ \lambda ^\theta _{1},\, \ldots ,\, \lambda ^\theta _{{n_\text {MTU}}}\right] ^T\in {\mathbb {R}}^{n_\text {MTU}}$ to the associated (low-level) $\lambda$-controller in the structural layer. This can be seen as a re-interpretation of the movement plan in terms of nominal muscle lengths. The nominal lengths ${\varvec{\lambda }}^\theta$ are prepared to feed the model representation of the mono-synaptic spinal cord reflex arc [$\lambda$-controller (10, 11)]. The output of the cascaded $\theta _\lambda$- and $\lambda$-controllers, which form a hierarchical control substructure of the overall architecture, is the stimulation signal ${\varvec{u}}^{\theta \lambda }(t)=\left[ u^{\theta \lambda }_{1},\, \ldots ,\, u^{\theta \lambda }_{{n_\text {MTU}}}\right] ^T\in {\mathbb {R}}^{n_\text {MTU}}$, with ${\varvec{u}}^{\theta \lambda }(t):={\varvec{u}}^\lambda (t,{\varvec{\lambda }}(t)={\varvec{\lambda }}^\theta (t))$ according to (10,11).

The second controller is termed the $\theta$-controller (for details, see Sect. 2.2.2b). It likewise takes the desired state ${\varvec{\theta }}^\text {des}\in {\mathbb {R}}^{{n_\theta }}$ and uses the angle–stimulation Jacobian ${J}^{{\varvec{u}}{\varvec{\theta }}}={J}^{{\varvec{u}}{\varvec{\lambda }}}\cdot {J}^{{\varvec{\lambda }}{\varvec{\theta }}}\in {\mathbb {R}}^{{n_\text {MTU}}\times {n_\theta }}$, which additionally contains steady-state knowledge in ${J}^{{\varvec{u}}{\varvec{\lambda }}}\in {\mathbb {R}}^{{n_\text {MTU}}\times {n_\text {MTU}}}$ about the stimulation–length relation of the MTU’s activation dynamics, to transmit a second task-fulfilling contribution to the MTU stimulations. This contribution is ‘open-loop’ from the perspective of the structural layer, as bypassing the muscle spindle reflex path, even though ‘closed-loop’ from the perspective of the transformational layer. It consist of the two stimulation vectors ${\varvec{u}}^\theta (t) = \left[ u^\theta _{1},\, \ldots ,\, u^\theta _{{n_\text {MTU}}}\right] ^T \in {\mathbb {R}}^{n_\text {MTU}}$ [see (30)] and ${\varvec{u}}^\text {coc}_\text {ref}(t)=\left[ {u_{\text {ref},{1}}^\text {coc}},\, \ldots ,\, {u_{\text {ref},{{n_\text {MTU}}}}^\text {coc}}\right] ^T\in {\mathbb {R}}^{n_\text {MTU}}_{01}$ [see (33)], of which the sum is termed

13 $$\begin{aligned} {\varvec{u}}^\text {opn}_\text {task}={\varvec{u}}^\theta +{\varvec{u}}^\text {coc}_\text {ref} \end{aligned}$$

to be still in accordance with (12). The two addends to ${\varvec{u}}^\text {opn}_\text {task}$ play complementary roles in fulfilling the task: ${\varvec{u}}^\text {coc}_\text {ref}$ are arbitrary reference stimulation signals (base contraction levels, for details see Sect. 2.2.2c), and ${\varvec{u}}^\theta$ is set up to minimise the error between ${\varvec{u}}^\text {coc}_\text {ref}$ and the desired stimulation values that corresponds to ${\varvec{\theta }}^\text {des}$, namely, ${\varvec{u}}^\text {opn}_\text {task}$ itself.

To allow the system satisfying additional criteria or side conditions along with the movement task, a second co-contraction contribution ${\varvec{u}}^\text {coc}_\theta =[u_{\theta ,{1}}^\text {coc},\,\dots ,\,u_{\theta ,{{n_\text {MTU}}}}^\text {coc}]^T\in {\mathbb {R}}^{{n_\text {MTU}}}$ to ‘open-loop’ MTU stimulation can be provided by the transformational layer within the control architecture. This adjusts the stimulations of all MTUs that act on the same joint exactly without interfering with the fulfilment of the movement task (for details see Sect. 2.2.2d).

Taken together, four contributions of MTU stimulations have been distinguished that feed the two addends in (12). The addend ${\varvec{u}}^\lambda$ identifies with ${\varvec{u}}^{\theta \lambda }$, and ${\varvec{u}}^\text {opn}$ with ${\varvec{u}}^\theta +{\varvec{u}}^\text {coc}_\text {ref}+{\varvec{u}}^\text {coc}_\theta$:

14 $$\begin{aligned} {\varvec{u}}(t)=\underbrace{{\varvec{u}}^{\theta \lambda }(t)}_{\widehat{=}{\varvec{u}}^\lambda (t)} + \underbrace{{\varvec{u}}^\theta (t)+{\varvec{u}}^\text {coc}_\text {ref}(t)+{\varvec{u}}^\text {coc}_\theta }_{\widehat{=}{\varvec{u}}^\text {opn}(t)}. \end{aligned}$$

The summed stimulation signal in (14) constitutes the total output of the proposed hierarchical control architecture, which drives the actuators of the musculo-skeletal system.

The details on the calculations of these stimulation signals and their underlying control concepts are given in the following, starting with the hierarchical $\theta _\lambda$-controller in Sect. 2.2.2a, followed by the direct $\theta$-controller including co-contraction in Sect. 2.2.2b, c. The joint co-contraction ${\varvec{u}}^\text {coc}_\theta$ and its basis for construction are outlined in Sect. 2.2.2d.

*(a) The hierarchical* $\theta _\lambda$*-controller.* In this section, the mathematical particulars of the $\theta _\lambda$-controller are outlined. It is described here, how the postural plan ${\varvec{\theta }}^\text {des}$ (see Sect. 2.2.3a) is interpreted in the transformational layer, and how it is transformed to the structural layer to provide the corresponding desired MTU lengths ${\varvec{\lambda }}^\theta$ that lead to the hierarchically controlled stimulation signal ${\varvec{u}}^{\theta \lambda }$ in (14) (see also Fig. 4).

Based on the set of controlled joint angles ${\varvec{\theta }}(t)$, the vector of neural sensor delays to the transformational layer ${\varvec{\delta _\theta }}=\left[ \delta _{\theta ,1},\,\ldots ,\,\delta _{\theta ,{n_\theta }}\right] ^T\in {\mathbb {R}}^{{n_\theta }}$, and the vector of desired joint angles ${\varvec{\theta }}^\text {des}(t)$, firstly, the control error is defined as

15 $$\begin{aligned} {\varvec{\theta }}^\text {err}_{{\varvec{\delta _\theta }}}(t):={\varvec{\theta }}(t,{\varvec{\delta _\theta }})-{\varvec{\theta }}^\text {des}(t) \in {\mathbb {R}}^{{n_\theta }}, \end{aligned}$$

where ${\varvec{\theta }}(t,{\varvec{\delta _\theta }}):=\left[ \theta _1(t-\delta _{\theta ,1}),\,\ldots ,\,\theta _{{n_\theta }}(t-\delta _{\theta ,{n_\theta }})\right] ^T$ denotes the vector of joint angles, delayed by their respective individual delay times. This error signal describes the deviation of the actual known body posture ${\varvec{\theta }}$ from the desired state ${\varvec{\theta }}^\text {des}$. Subsequently, the PID control law

16 $$\begin{aligned} {\varvec{\theta }}^\text {PID}_{\theta _\lambda }:={P}_{\theta _\lambda }\cdot {\varvec{\theta }}^\text {err}_{\delta _\theta }+{D}_{\theta _\lambda }\cdot {\dot{{\varvec{\theta }}}}^\text {err}_{\delta _\theta }+ {I}_{\theta _\lambda }\cdot \int {\varvec{\theta }}^\text {err}_{\delta _\theta }\mathrm {d}t \in {\mathbb {R}}^{{n_\theta }} \end{aligned}$$

is used to create a robust and stabilising output of controlled joint angles, where the matrices ${P}_{\theta _\lambda }=\text {diag}(p_{\theta _\lambda ,1},\,\dots ,p_{\theta _\lambda ,{n_\theta }})\in {\mathbb {R}}^{{n_\theta }\times {n_\theta }}$, ${D}_{\theta _\lambda }=\text {diag}(d_{\theta _\lambda ,1},\,\dots ,\,d_{\theta _\lambda ,{n_\theta }})\in {\mathbb {R}}^{{n_\theta }\times {n_\theta }}$, and ${I}_{\theta _\lambda }=\text {diag}({\textsc {i}}_{\theta _\lambda ,1},\,\dots ,{\textsc {i}}_{\theta _\lambda ,{n_\theta }})\in {\mathbb {R}}^{{n_\theta }\times {n_\theta }}$ are diagonal control matrices, with parameters $p_{\theta _\lambda ,i}>0$, $d_{\theta _\lambda ,i}>0$, and ${\textsc {i}}_{\theta _\lambda ,i}>0$. The output ${\varvec{\theta }}^\text {PID}_{\theta _\lambda }(t)$ allows to fix the values of desired CE lengths ${\varvec{\lambda }}^\theta (t)$ (see (21) below). It mainly scales (by ${P}_{\theta _\lambda }$) with the control error signal ${\varvec{\theta }}^\text {err}_{\delta _\theta }(t)$, while a modifying angular-rate-dependent (by ${D}_{\theta _\lambda }$) contribution, and some memory (by ${I}_{\theta _\lambda }\cdot \int {\varvec{\theta }}^\text {err}_{\delta _\theta }\mathrm {d}t$) of the angle error are added, with the latter eliminating any residual control deviations.

To eventually fix the desired CE lengths ${\varvec{\lambda }}^\theta (t)$ (21), the output of the $\theta _\lambda$-PID control law (16) from the transformational layer must be transformed to the structural layer. Instead of transforming the joint angles directly to CE lengths, changes of the joint angles $\partial {\varvec{\theta }}$ are transformed to changes of muscle lengths $\partial {\varvec{l}}^{\mathrm{CE}}$, which corresponds to answering the question how much the contractile parts of the muscles need to contract (or relax) to reach ${\varvec{\theta }}^\text {des}$. Such a transformation has already been discussed in literature (Pellionisz and Llinás 1985) and can be achieved by a Jacobian matrix of the form

17 $$\begin{aligned} {J}^{{\varvec{\lambda }}{\varvec{\theta }}}:=\frac{\partial {\varvec{l}}^{\mathrm{CE}}}{\partial {\varvec{\theta }}}\in {\mathbb {R}}^{{n_\text {MTU}}\times {n_\theta }} \quad \Leftrightarrow \quad \partial {\varvec{l}}^{\mathrm{CE}}={J}^{{\varvec{\lambda }}{\varvec{\theta }}}\cdot \partial {\varvec{\theta }}. \end{aligned}$$

Note, that this angle–length Jacobian has a similar definition as the matrix of MTU moment arms (8). In fact, ${J}^{{\varvec{\lambda }}{\varvec{\theta }}}$ can approximately be derived from the matrix of moment arms ${{R}}({\varvec{\theta }})$, by using its definition (8) and the length constraint ${\varvec{l}}^{\mathrm{MTU}}={\varvec{l}}^{\mathrm{CE}}+{\varvec{l}}^{\mathrm{SEE}}$ (3):

$$\begin{aligned} {{R}}({\varvec{\theta }})=\frac{\partial {\varvec{l}}^{\mathrm{MTU}}}{\partial {\varvec{\theta }}} = \frac{\partial \left( {\varvec{l}}^{\mathrm{CE}}+{\varvec{l}}^{\mathrm{SEE}}\right) }{\partial {\varvec{\theta }}}=\frac{\partial {\varvec{l}}^{\mathrm{CE}}}{\partial {\varvec{\theta }}}+ \frac{\partial {\varvec{l}}^{\mathrm{SEE}}}{\partial {\varvec{\theta }}}, \end{aligned}$$

where $\frac{\partial {\varvec{l}}^{\mathrm{SEE}}}{\partial {\varvec{\theta }}}$ can be rewritten to $\frac{\partial {\varvec{l}}^{\mathrm{SEE}}}{\partial {\varvec{l}}^{\mathrm{CE}}}\frac{\partial {\varvec{l}}^{\mathrm{CE}}}{\partial {\varvec{\theta }}}$, which allows the following formulation of the angle–length Jacobian

18 $$\begin{aligned} {J}^{{\varvec{\lambda }}{\varvec{\theta }}}=\left( {I}_{{n_\text {MTU}}}+\frac{\partial {\varvec{l}}^{\mathrm{SEE}}}{\partial {\varvec{l}}^{\mathrm{CE}}}\right) ^{-1}\cdot {{R}}({\varvec{\theta }}). \end{aligned}$$

Here, ${I}_{{n_\text {MTU}}}\in {\mathbb {R}}^{{n_\text {MTU}}\times {n_\text {MTU}}}$ is the identity matrix of dimension ${n_\text {MTU}}$, and $\frac{\partial {\varvec{l}}^{\mathrm{SEE}}}{\partial {\varvec{l}}^{\mathrm{CE}}}=\mathrm {diag\left( {\partial l^{\mathrm{SEE}}_i}/{\partial l^{\mathrm{CE}}_i}\right) }$, with $i=1\,\ldots ,\,{n_\text {MTU}}$, describes the change of the length of the SEE w.r.t. the change of the length of the CE. This relation can be approximated from knowing the stiffnesses of the elements of the MTU under quasi-static assumptions. Details for this calculation are given in Appendix B, using the force laws of the Hill-type muscle model (Haeufle et al. 2014a) (see also Appendix A.1). Thus, the angle–length Jacobian (18) contains information about the muscles’ routings (moment arms), adjusted by internal stiffness ratios of the active CEs (at the current activity level) and the respective passive SEEs (contraction dynamics). Exactly this Jacobian matrix is used here for solving the muscle-joint redundancy of the musculo-skeletal system. Its resolved algebraic form (18, 76) constitutes the implementation of the body’s biophysical actuator properties within the control architecture.

For transforming—by means of the angle–length Jacobian (17)—the control error (15), as determined in the transformational layer in terms of joint angles, to its error equivalent (10) in the structural layer in terms of CE lengths, a first-order Taylor approximation is applied at the set-point of the current joint angles ${\varvec{\theta }}(t)$ and the CE lengths ${\varvec{l}}^{\mathrm{CE}}(t)$, with the integration limits of ${\varvec{\theta }}^\text {des}$ and ${\varvec{\lambda }}^\theta \widehat{=}{\varvec{l}}^{\mathrm{CE}}\vert _{{\varvec{\theta }}^\text {des}}$, respectively:

19 $$\begin{aligned} {\varvec{l}}^\text {err}(t) = {J}^{{\varvec{\lambda }}{\varvec{\theta }}}\cdot {\varvec{\theta }}^\text {err}(t)+ {\mathcal {O}}({{\varvec{\theta }}^\text {err}}^2). \end{aligned}$$

The detailed calculation thereof is given in Appendix B.2.

Finally, to complete the hierarchical $\theta _\lambda$-controller, the vector of desired CE lengths ${\varvec{\lambda }}^\theta (t)$ and their time rates ${\dot{{\varvec{\lambda }}}}^\theta$ must be provided as input to the $\lambda$-controller (11). The vector ${\varvec{\lambda }}^\theta (t)$ is obtained by equating ${\varvec{l}}^\text {err}(t)$ in (19) with (10) and solve the remaining equation for ${\varvec{\lambda }}(t)$. As these desired CE lengths are assigned within the transformational layer, and based on angle information, they are written as ${\varvec{\lambda }}^\theta (t)$. Additionally, to compensate approximation errors, and to ensure that ${\varvec{\theta }}^\text {err}(t)$ approaches zero along with ${\varvec{\lambda }}^\theta (t)\rightarrow {\varvec{l}}^{\mathrm{CE}}(t)$, the output ${\varvec{\theta }}^\text {PID}_{\theta _\lambda }(t)$ of the $\theta _\lambda$-PID controller (16), rather than ${\varvec{\theta }}^\text {err}(t)$, is used as input for the Jacobian transformation (19). With this, and by neglecting the higher-order terms ${\mathcal {O}}({{\varvec{\theta }}^\text {err}}^2)$, and further considering the delay times $\delta _\theta$ of the peripheral sensor signals [${\varvec{l}}^{\mathrm{CE}}(t,\delta _\theta$), (10)] in the transformational layer, ${\varvec{l}}^\text {err}(t)$ in (19) now writes

20 $$\begin{aligned} {\varvec{l}}^\text {err}_{{\varvec{\delta _\theta }}}={\varvec{l}}^{\mathrm{CE}}\left( t,\delta _\theta \right) -{\varvec{\lambda }}^\theta (t)\approx {J}^{{\varvec{\lambda }}{\varvec{\theta }}}\cdot {{\varvec{\theta }}^\text {PID}_{\theta _\lambda }}(t), \end{aligned}$$

which eventually yields (compare to 10)

21 $$\begin{aligned} {\varvec{\lambda }}^\theta (t):={\varvec{l}}^{\mathrm{CE}}\left( t,\delta _\theta \right) -{J}^{{\varvec{\lambda }}{\varvec{\theta }}}\cdot {{\varvec{\theta }}^\text {PID}_{\theta _\lambda }}(t), \end{aligned}$$

that is, a reliable estimate of how the respective CE lengths have to be adapted to fulfil the task, i.e. reach ${\varvec{\theta }}^\text {des}$. In this, an estimation of the desired contraction velocity ${\dot{{\varvec{\lambda }}}}^\theta$ is already included, since the definition of the angle–length Jacobian matrix (17) can also be read as (Sherman et al. 2013)

22 $$\begin{aligned} {J}^{{\varvec{\lambda }}{\varvec{\theta }}}=\left. \frac{\partial {\varvec{l}}^{\mathrm{CE}}}{\partial {{\varvec{\theta }}}}=\frac{\text {d} {\varvec{l}}^{\mathrm{CE}}}{\text {d}t}\big /\frac{\text {d} {\varvec{\theta }}}{\text {d}t}\right. = \frac{\dot{{\varvec{l}}}^\text {CE}}{{\dot{{\varvec{\theta }}}}}, \end{aligned}$$

which can be used for transforming desired joint angle velocities ${\dot{{\varvec{\theta }}}}^\text {des}$. The (linear summation inherent to the) $\theta _\lambda$-PID controller (16) delivers an input for the layer transformation (21), that includes a velocity component to be transformed along in a natural way. Therefore, the transformational layer is not required to generate a separate signal ${\dot{{\varvec{\lambda }}}}^\theta$ (i.e. ${\dot{{\varvec{\lambda }}}}^\theta =0$) for feeding the structural layer.

By making use of the $\lambda$-control law (11) with the hierarchical input signal ${\varvec{\lambda }}^\theta$ according to (21), the stimulation signal contribution by the $\theta _\lambda$-controller to (14) is

23 $$\begin{aligned} {\varvec{u}}^{\theta \lambda }(t):={P}_\lambda \cdot \left( {\varvec{l}}^{\mathrm{CE}}(t,\delta _\lambda )-{\varvec{\lambda }}^\theta (t)\right) +{D}_\lambda \dot{{\varvec{l}}}^\text {CE}(t,\delta _\lambda ), \end{aligned}$$

that is, the closed-loop, low-level feedback control law in the structural layer, to which the input signals ${\varvec{\lambda }}^\theta (t)$ originate from the mid-level control in the transformational layer. Due to the hierarchical cascade ${\varvec{u}}^{\theta \lambda }({\varvec{\lambda }}^\theta ({\varvec{\theta }}^\text {des}))$, with distributed PID and PD controllers, this stimulation contribution is task-fulfilling, even under gravity, notably because of the integrative part^2^ of the $\theta _\lambda$-controller.

By following the hybrid control approach (12, 14) (Bayer et al. 2017; Kistemaker et al. 2006), an ‘open-loop’ stimulation contribution, which is, in addition, physiological meaningful, can improve the control performance (Kistemaker et al. 2006). The ‘open-loop’ part of the present control architecture is presented in the following: it produces a stimulation signal that is based on the set of desired joint angles ${\varvec{\theta }}^\text {des}$ (postural plan), without making draft on the low-level control mechanisms in the structural layer.

*(b) The direct* $\theta$-*controller with co-contraction.* The approach to the direct $\theta$-controller with co-contraction is analogous to the hierarchical $\theta _\lambda$ controller from above. The desired joint angles ${\varvec{\theta }}^\text {des}(t)$ are compared to the actual joint angles ${\varvec{\theta }}(t)$, and the resulting control errors ${\varvec{\theta }}^\text {err}_{\delta _\theta }$, with latencies ${\varvec{\delta _\theta }}$ according to (15), serves as input to a PID control law in the transformational layer

24 $$\begin{aligned} {\varvec{\theta }}^\text {PID}_{\theta }:={P}_\theta \cdot {\varvec{\theta }}^\text {err}_{\delta _\theta }+{D}_\theta \cdot {\dot{{\varvec{\theta }}}}^\text {err}_{\delta _\theta } + {I}_\theta \cdot \int {\varvec{\theta }}^\text {err}_{\delta _\theta }\mathrm {d}t, \end{aligned}$$

with the diagonal control matrices ${P}_\theta =\text {diag}(p_{\theta ,1},\,\dots ,\,p_{\theta ,{n_\theta }})$, ${D}_\theta =\text {diag}(d_{\theta ,1},\,\dots ,\,d_{\theta ,{n_\theta }})$, ${I}_\theta =\text {diag}({\textsc {i}}_{\theta ,1},\,\dots ,\,{\textsc {i}}_{\theta ,{n_\theta }})$, containing the components $p_{\theta ,i}>0$, $d_{\theta ,i}>0$ and ${\textsc {i}}_{\theta ,i}>0$.

As an intermediate but only half-way step, the output of this $\theta$-controller can be transformed through the angle–length Jacobian ${J}^{{\varvec{\lambda }}{\varvec{\theta }}}$ (17, 18), in a first instance, to the transformational layer. However, and in contrast to the $\theta _\lambda$-controller, the low-level $\lambda$ control law in the structural layer is eventually in fact bypassed by an additional Jacobian transformation ${J}^{{\varvec{u}}{\varvec{\lambda }}}$, obtaining the stimulation contribution ${\varvec{u}}^\theta$ directly. By constructing this (length–stimulation) Jacobian ${J}^{{\varvec{u}}{\varvec{\lambda }}}$ (26), an angle–stimulation Jacobian ${J}^{{\varvec{u}}{\varvec{\theta }}}$ can be composed that contains ${J}^{{\varvec{\lambda }}{\varvec{\theta }}}$ and immediately transforms the changes of joint angles $\partial {\varvec{\theta }}$ in the transformational layer to changes of MTU stimulations $\partial {\varvec{u}}$ in the structural layer:

25 $$\begin{aligned} {J}^{{\varvec{u}}{\varvec{\theta }}}={J}^{{\varvec{u}}{\varvec{\lambda }}}\cdot {J}^{{\varvec{\lambda }}{\varvec{\theta }}}= & {} \frac{\partial {\varvec{u}}}{\partial {\varvec{l}}^{\mathrm{CE}}}\cdot \frac{\partial {\varvec{l}}^{\mathrm{CE}}}{\partial {\varvec{\theta }}}=\frac{\partial {\varvec{u}}}{\partial {\varvec{\theta }}} \in {\mathbb {R}}^{{n_\text {MTU}}\times {n_\theta }}\nonumber \\ \partial {\varvec{u}}= & {} {J}^{{\varvec{u}}{\varvec{\theta }}}\cdot \partial {\varvec{\theta }}, \end{aligned}$$

with ${J}^{{\varvec{\lambda }}{\varvec{\theta }}}$ known from (18) and

26 $$\begin{aligned} {J}^{{\varvec{u}}{\varvec{\lambda }}}:=\frac{\partial {\varvec{u}}}{\partial {\varvec{l}}^{\mathrm{CE}}} \in {\mathbb {R}}^{{n_\text {MTU}}\times {n_\text {MTU}}}. \end{aligned}$$

For calculating this length–stimulation Jacobian ${J}^{{\varvec{u}}{\varvec{\lambda }}}$, structural knowledge of the muscle activation dynamics (7) and their steady state (${\dot{\gamma }}_k=0$, $\gamma _k=u_k$), symbolised by $a^\text {ss}$, is utilised. While the detailed calculation of ${J}^{{\varvec{u}}{\varvec{\lambda }}}$ is given in Appendix C, the scalar result $j^{{\varvec{u}}{\varvec{\lambda }}}_k$ for the diagonal Jacobian matrix ${J}^{{\varvec{u}}{\varvec{\lambda }}}=\text {diag}(j^{{\varvec{u}}{\varvec{\lambda }}}_k)$ for the *k*-th MTU follows as

27 $$\begin{aligned} \left. j^{{\varvec{u}}{\varvec{\lambda }}}_k(t)\right| _{a^\text {ss}}=-\frac{u_k(t)}{l^{\mathrm{CE}}_k(t)}, \end{aligned}$$

i.e. the instantaneous change of $u_k(t)$ w.r.t. a change of $l^{\mathrm{CE}}_k(t)$, assuming steady-state conditions $a^\text {ss}_k(u_k(t),l^{\mathrm{CE}}_k(t))$.

For using the length–stimulation Jacobian (27) in the $\theta$-controller, a first-order Taylor approximation is applied at the set-point of the current CE lengths ${\varvec{l}}^{\mathrm{CE}}$ and reference stimulation levels ${\varvec{u}}^\text {coc}_\text {ref}$ (see Sect. 2.2.2c) with the integration limits of ${\varvec{\lambda }}^\theta ={\varvec{l}}^{\mathrm{CE}}\vert _{{\varvec{\theta }}^\text {des}}$ and ${\varvec{u}}^\text {opn}_\text {task}$. The calculation of this Taylor approximation is given in Appendix C.1. It implies the definition of the stimulation error

28 $$\begin{aligned} {\varvec{u}}^\text {err}(t):={\varvec{u}}^\text {coc}_\text {ref}(t)-{\varvec{u}}^\text {opn}_\text {task}(t) \in {\mathbb {R}}^{{n_\text {MTU}}}, \end{aligned}$$

where ${\varvec{u}}^\text {coc}_\text {ref}$ is an arbitrary but nonzero reference stimulation and ${\varvec{u}}^\text {opn}_\text {task}$ is a task-fulfilling stimulation contribution that eventually corresponds to the postural plan ${\varvec{\theta }}^\text {des}$. The Taylor approximation yields

29 $$\begin{aligned} {\varvec{u}}^\text {err}(t)=\left. {J}^{{\varvec{u}}{\varvec{\lambda }}}\right| _{{\varvec{u}}={\varvec{u}}^\text {coc}_\text {ref}}\cdot {\varvec{l}}^\text {err}(t) +{\mathcal {O}}({{\varvec{l}}^\text {err}}^2). \end{aligned}$$

With this, and neglecting all higher-order terms ${\mathcal {O}}({{\varvec{l}}^\text {err}}^2)$, (28, 29) can be solved for the stimulation contribution ${\varvec{u}}^\text {opn}_\text {task}(t)$ (13) sought after.

To complete the construction of the $\theta$-controller, similar as for the $\theta _\lambda$-controller [see (21)], ${\varvec{l}}^\text {err}(t)$ is now replaced in (29) by making use of (19) and the angle–length Jacobian ${J}^{{\varvec{\lambda }}{\varvec{\theta }}}$ (17) as well as the $\theta$-PID controller output ${\varvec{\theta }}^\text {PID}(t)$ (24) as an estimate of ${\varvec{\theta }}^\text {err}(t)$. This gives a robust, stabilising estimate

30 $$\begin{aligned} {\varvec{u}}^\theta (t):=-\left. {J}^{{\varvec{u}}{\varvec{\lambda }}}\right| _{{\varvec{u}}={\varvec{u}}^\text {coc}_\text {ref}}\cdot {J}^{{\varvec{\lambda }}{\varvec{\theta }}}\cdot {\varvec{\theta }}^\text {PID}_{\theta }(t) \end{aligned}$$

of ${\varvec{u}}^\text {err}(t)$ (28, 29) , which substantiates the proposed ansatz for the $\theta$-controller by approximating ${\varvec{u}}^\text {opn}_\text {task}$ according to (13) by

31 $$\begin{aligned} {\varvec{u}}^\text {opn}_\text {task}(t)&:\approx&{\varvec{u}}^\text {coc}_\text {ref}(t)-\left. {J}^{{\varvec{u}}{\varvec{\lambda }}}\right| _{{\varvec{u}}={\varvec{u}}^\text {coc}_\text {ref}}\cdot {J}^{{\varvec{\lambda }}{\varvec{\theta }}}\cdot {\varvec{\theta }}^\text {PID}_{\theta }(t) \end{aligned}$$

32 $$\begin{aligned}= & {} {\varvec{u}}^\text {coc}_\text {ref}(t)-\underbrace{\left. {J}^{{\varvec{u}}{\varvec{\theta }}}\right| _{{\varvec{u}}={\varvec{u}}^\text {coc}_\text {ref}} \cdot {\varvec{\theta }}^\text {PID}_{\theta }(t)}_{:=-{\varvec{u}}^\theta (t)}. \end{aligned}$$

Here, ${\varvec{u}}^\theta$ is the output of the $\theta$-PID controller that is designed to adapt the reference stimulation ${\varvec{u}}^\text {coc}_\text {ref}$ to the task-fulfilling ‘open-loop’ (from the perspective of the structural layer) stimulation contribution ${\varvec{u}}^\text {opn}_{\text {task}}$. Thus, the base contraction level ${\varvec{u}}^\text {coc}_\text {ref}$ is not required to yield the desired equilibrium and can be chosen more freely.

*(c) Choosing the base reference stimulation level* ${\varvec{u}}^\text {coc}_\text {ref}$. The assignment of the base reference stimulations ${\varvec{u}}^\text {coc}_\text {ref}$ is technically simple. Firstly, a basis for the $k=1\,\ldots \,{n_\text {MTU}}$ MTU stimulations is constructed by the linear independent vectors ${\mathbf {e}}_k^u=[{e}_{k,1},\,\dots ,\,{e}_{k,{{n_\text {MTU}}}}]\in {\mathbb {R}}_{01}^{n_\text {MTU}}$, with ${e}_{k,i}^u=0$ for $i\ne k$ and ${e}_{k,i}^u=1$ for $i=k$. The reference stimulation ${\varvec{u}}^\text {coc}_\text {ref}$ is then obtained by choosing a base contraction parameter $\zeta ^u_k\in {\mathbb {R}}_{01}$ for each MTU to scale the basis of the stimulation vectors:

33 $$\begin{aligned} {\varvec{u}}^\text {coc}_\text {ref}=\sum _{k=1}^{n_\text {MTU}}\zeta ^u_k\cdot {\mathbf {e}}_k^u\in {\mathbb {R}}^{{n_\text {MTU}}}_{01}. \end{aligned}$$

With this, an arbitrary reference stimulation level can be assigned to each MTU individually. As already outlined in the previous section, the presented control architecture does not require ${\varvec{u}}^\text {coc}_\text {ref}$ to be in accordance with the specific movement task that is specified by ${\varvec{\theta }}^\text {des}$. Moreover, in the simulation tasks examined in Sect. 3.1, assigning one and the same co-contraction parameter value $\zeta _k^u={\zeta ^u}^{*}$ to all MTUs did the job.

*(d) Joint-based co-contraction as an additional criterion.* Above, the two Jacobian matrices ${J}^{{\varvec{\lambda }}{\varvec{\theta }}}$ [see (17, 18)] and ${J}^{{\varvec{u}}{\varvec{\lambda }}}$ [see (26, 27)] that resolve the muscle redundancy have been presented. In composition ${J}^{{\varvec{u}}{\varvec{\theta }}}={J}^{{\varvec{u}}{\varvec{\lambda }}}{J}^{{\varvec{\lambda }}{\varvec{\theta }}}$ [see (25)], they even allow to transform joint angle changes $\partial {\varvec{\theta }}$ immediately to MTU stimulation changes $\partial {\varvec{u}}$. This finding can be further exploited for allowing the system to satisfy criteria beyond fulfilling the primary task. A criterion added to fulfilling a specific movement task may be the level of joint stiffness or speed of execution, while co-contraction has a significant impact on both joint stiffness (Bayer et al. 2017; De Serres and Milner 1991; Gribble et al. 2003; Kistemaker et al. 2007; Milner 2002; Milner et al. 1995) and movement speed (Bayer et al. 2017; Gribble et al. 1998; Kistemaker et al. 2006; McIntyre and Bizzi 1993). It is derived in the following how this architectural integration potential, which is implicit to the muscle redundancy imprinted in ${J}^{{\varvec{\lambda }}{\varvec{\theta }}}$ (and ${J}^{{\varvec{u}}{\varvec{\theta }}}$, respectively), can be used to set a functionally desired joint-related co-contracting contribution to any of MTU stimulations.

In addition to setting, as described in Sect. 2.2.2c, a base contraction level $\zeta ^u_k\in {\mathbb {R}}^{{n_\text {MTU}}}$ for each MTU individually, a second co-contraction parameter $\zeta ^\theta _j\in {\mathbb {R}}$ for each of the $j=1\,\ldots \,{n_\theta }$ muscle-actuated joint DoFs ${\varvec{\theta }}$ of the skeletal system can be deployed. The redundant nature of the embodiment of the ${n_\text {MTU}}$ muscles opens a manifold of dimension (${n_\text {MTU}}-{n_\theta }$) to assign MTU stimulation contributions ${\varvec{u}}^\text {coc}_\theta$ derived from these co-contraction parameters $\zeta ^\theta _j$. To exactly not interfere with the movement task specified by ${\varvec{\theta }}^\text {des}$, the joint co-contraction ${\varvec{u}}^\text {coc}_\theta$ is chosen based on the null-space of the (Moore–Penrose pseudo) inverse ${{J}^{{\varvec{u}}{\varvec{\theta }}}}^\dagger$ of ${J}^{{\varvec{u}}{\varvec{\theta }}}$. The null-space—or kernel, respectively—of ${{J}^{{\varvec{u}}{\varvec{\theta }}}}^\dagger$ is the set of infinitesimal MTU stimulation changes $\partial {\varvec{u}}$ that are mapped via ${{J}^{{\varvec{u}}{\varvec{\theta }}}}^\dagger$ to the null vector of angular changes, i.e. $\partial {\varvec{\theta }}=0$. This means that the discrete addition of any sufficiently small vector ${\varvec{u}}^\text {coc}_\theta$ from the null space of ${{J}^{{\varvec{u}}{\varvec{\theta }}}}^\dagger$ to the MTU stimulation ${\varvec{u}}$ [i.e. (14)] results in the joint angles ${\varvec{\theta }}(t)$ not significantly changing; therefore, adding ${\varvec{u}}^\text {coc}_\theta$ does not interfere with the movement task ${\varvec{\theta }}^\text {des}$.

Mathematically the null-space is obtained by solving

34 $$\begin{aligned} 0\overset{!}{=}\partial {\varvec{\theta }}={{J}^{{\varvec{u}}{\varvec{\theta }}}}^\dagger \partial {\varvec{u}}, \end{aligned}$$

e.g. via Gaussian elimination, where ${{J}^{{\varvec{u}}{\varvec{\theta }}}}^\dagger$ is the Moore–Penrose pseudo-inverse of ${J}^{{\varvec{u}}{\varvec{\theta }}}$ defined by

35 $$\begin{aligned} {{J}^{{\varvec{u}}{\varvec{\theta }}}}^\dagger :=({{J}^{{\varvec{u}}{\varvec{\theta }}}}^T\cdot {J}^{{\varvec{u}}{\varvec{\theta }}})^{-1}\cdot {{J}^{{\varvec{u}}{\varvec{\theta }}}}^T\in {\mathbb {R}}^{{n_\theta }\times {n_\text {MTU}}}. \end{aligned}$$

Note here, that the pseudo-inverse (35) can only be calculated for ${J}^{{\varvec{u}}{\varvec{\theta }}}$ having full column rank ($\text {rk}({J}^{{\varvec{u}}{\varvec{\theta }}})={n_\theta }$), which is not the case, e.g. if the joint DoFs ${\tilde{{\varvec{\theta }}}}$ that are not actuated by muscles are considered in the control architecture. In all feasible cases, the solution of (34) yields a basis of the null-space (kernel) of dimension $\text {dim}(\text {ker}({{J}^{{\varvec{u}}{\varvec{\theta }}}}^\dagger ))=({n_\text {MTU}}-{n_\theta })$, which equals to $\text {dim}(\text {ker}({{J}^{{\varvec{u}}{\varvec{\theta }}}}^\dagger ))={n_\theta }$ in the model that is used in this study. Thus, the basis of the null-space has exactly the same size as the set of MTU actuated joint angles ${\varvec{\theta }}$, allowing ${n_\theta }$ additional DoFs to satisfy further movement criteria (see examples above) along with fulfilling the primary movement task. By this, the transformational layer of the control architecture proposed here facilitates to apply joint-based co-contraction: The linearly independent basis vectors ${\mathbf {e}}^\theta _j$ ($\Vert {\mathbf {e}}^\theta _j\Vert =1$, $j=1\,\ldots \,{n_\theta }$) that build the basis of the null-space obtained from (34) are simply scaled (similar to the base reference stimulation from Sect. 2.2.2c) by the arbitrary co-contraction parameters $\zeta ^\theta _j\in {\mathbb {R}}$ to obtain a joint co-contraction contribution

36 $$\begin{aligned} {\varvec{u}}^\text {coc}_\theta =\sum _{j=1}^{{n_\theta }}\zeta ^\theta _j\cdot {\mathbf {e}}^\theta _j \in {\mathbb {R}}_{01}^{n_\text {MTU}} \end{aligned}$$

that fulfils (34). In this stimulation contribution, the implicit resolution of the muscle redundancy in ${J}^{{\varvec{u}}{\varvec{\theta }}}$ by anatomical knowledge (moment arms, contraction and activation dynamics) is exploited to generate synergistic MTU stimulations that do not interfere with fulfilling the movement task ${\varvec{\theta }}^\text {des}$, with yet allowing to vary simultaneously but in a coordinated way the activity level of all muscles that act on the same joint.

#### High-level control in the conceptional layer

According to the proposed control architecture (Fig. 4), the first step in applying high-level control concepts to drive the musculo-skeletal system is to feed the outputs of the conceptional layer as desired values ${\varvec{\theta }}^\text {des}$ (postural plan) into the two joint controllers in the transformational layer. Depending on the movement task itself, there may be different conceptional approaches to draft a movement plan leading to a valid choice of ${\varvec{\theta }}^\text {des}$.

One approach would be to perform a joint angle-based trajectory planning to determine ${\varvec{\theta }}^\text {des}(t)$. The resulting controlled MTU stimulations ${\varvec{u}}(t)$ then strive to drive the system to follow this trajectory. This may be a suitable method to use motor control concepts on the kinematic level to synthesise musculo-skeletal movements.

As an alternative approach, it is also possible to implement motor control concepts that calculate desired state-dependent joint torques. One example is maintaining upright stance, where a joint angle-based trajectory control may not be sufficient, as the mechanical system (1) is unstable around the upright position. In this case, several (e.g. system-theoretic) concepts exist which propose state-dependent (desired) torques ${\varvec{\tau }}^\text {des}(t, \mathrm{model parameters})$ to stabilise the unstable upright equilibrium pose (Günther and Wagner 2016; Rozendaal and van Soest 2005; Edwards 2007; Alexandrov et al. 2001).

In the following, it is derived how such a conceptional formulation of the movement task in terms of desired torques ${\varvec{\tau }}^\text {des}$ can be transformed to the postural plan ${\varvec{\theta }}^\text {des}$, using anatomical and tissue knowledge (muscle geometry and stiffnesses, respectively). Determined by the scheme of the proposed hierarchical control architecture, the movement plan is re-interpreted in terms of the coordinates of MTU stimulations ${\varvec{u}}(t)$ on the architecture’s output side, which result in muscle forces and corresponding joint torques ${\varvec{\tau }}^\text {MTU}$ that (hopefully) fulfil the movement plan ${\varvec{\tau }}^\text {des}$.

*(a) Fixing the postural plan* ${\varvec{\theta }}^\text {des}$ *by a torque-based strategy.* Given a specific movement task, it is for now assumed that task-specific, state-dependent (desired) torques

37 $$\begin{aligned} {\varvec{\tau }}^\text {des}(t)={\varvec{\tau }}^\text {des}(t, \mathrm{model parameters}), \end{aligned}$$

can be formulated that fulfil the movement task. Such a conceptional formulation of the movement task in terms of joint torques may be dependent on (a subset of) the mechanical state ${\varvec{q}}$ only, forming a closed-loop, conceptional controller of the mechanical system (1). For the example of a conceptional, torque-based strategy to maintain upright stance, refer to Sect. 3.1.

To deploy such a torque-based concept in the presented control architecture, an additional control law on the level of joint torques is formulated, which is subsequently transformed to the level of joint angles. The resulting signal can then be used as an input for the joint controllers (16) and (24). This firstly requires the definition of the error signal of joint torques with the latency times ${\varvec{\delta _\tau }}=\left[ \delta _{\tau ,1},\, \ldots ,\, \delta _{\tau ,{n_\theta }}\right] ^T\in {\mathbb {R}}^{{n_\theta }}$:

38 $$\begin{aligned} {\varvec{\tau }}^\text {err}_{{\varvec{\delta _\tau }}}(t)={\varvec{\tau }}^\text {MTU}(t,{\varvec{\delta _\tau }})-{\varvec{\tau }}^\text {des}(t), \end{aligned}$$

where ${\varvec{\tau }}^\text {MTU}(t,{\varvec{\delta _\tau }}):=[\tau ^\text {MTU}_1(t-\delta _{\tau ,1}),\,\ldots ,\,\tau ^\text {MTU}_{{n_\theta }} (t-\delta _{\tau ,{n_\theta }})]^T$ is the vector of joint torques, delayed by their respective individual delay times and ${\varvec{\tau }}^\text {des}=[\tau ^\text {des}_1,\, \ldots \, \tau ^\text {des}_{{n_\theta }}]\in {\mathbb {R}}^{n_\theta }$ is the input to the system that results from the control concept (37) above. This error is controlled via the following PID control law on joint torques:

39 $$\begin{aligned} {\varvec{\tau }}^\text {PID}:={P}_\tau \cdot {\varvec{\tau }}^\text {err}_{{\varvec{\delta _\tau }}}(t) +{D}_\tau \cdot {\dot{{\varvec{\tau }}}}^\text {err}_{{\varvec{\delta _\tau }}}(t) + {I}_\tau \cdot \int {\varvec{\tau }}^\text {err}_{{\varvec{\delta _\tau }}}(t) \mathrm {d}t, \end{aligned}$$

where the control matrices ${P}_\tau =\text {diag}(p_{\tau ,1},\,\dots ,\,p_{\tau ,{n_\theta }})$, ${D}_\tau =\text {diag}(d_{\tau ,1},\,\dots ,\,d_{\tau ,{n_\theta }})$, and ${I}_\tau =\text {diag}({\textsc {i}}_{\tau ,1},\,\dots ,\,{\textsc {i}}_{\tau ,{n_\theta }})$ are diagonal, with $p_{\tau ,i}>0$, $d_{\tau ,i}>0$ and ${\textsc {i}}_{\tau ,i}>0$. The controlled signal ${\varvec{\tau }}^\text {PID}$ in the conceptional layer is subsequently transformed to the (transformational) layer of joint angles via the torque–angle Jacobian matrix

40 $$\begin{aligned} {J}^{{\varvec{\theta }}{\varvec{\tau }}}:=\frac{\partial {\varvec{\theta }}}{\partial {\varvec{\tau }}}\in {\mathbb {R}}^{{n_\theta }\times {n_\theta }}, \end{aligned}$$

which is the inverse of the joint stiffness matrix $K_\theta =\partial {\varvec{\tau }}/ \partial {\varvec{\theta }}$ (Stanev and Moustakas 2019). Calculated further, for the assumption of small changes in the muscles moment arms, this yields

41 $$\begin{aligned} {J}^{{\varvec{\theta }}{\varvec{\tau }}}=\left( -{{R}}^T\cdot \frac{\partial {\varvec{f}}^\text {MTU}}{\partial {\varvec{l}}^{\mathrm{CE}}}\cdot {J}^{{\varvec{\lambda }}{\varvec{\theta }}}\right) ^{-1}. \end{aligned}$$

Details on the calculations of ${J}^{{\varvec{\theta }}{\varvec{\tau }}}$ are given in Appendix D. In the closed form (41) of this Jacobian, the required anatomical knowledge is again apparent and consists of moment arms, MTU stiffnesses, and MTU-internal stiffness relations [see also (18)].

Similar as in (19), the torque error can be transformed to an error in joint angles using a first-order Taylor approximation (see Appendix D.1), leading to the definition of a signal of desired joint angles, based on the controller output of joint torques:

42 $$\begin{aligned} {\varvec{\theta }}^\text {des}_\tau (t):={\varvec{\theta }}\left( t,\delta _\tau \right) -{J}^{{\varvec{\theta }}{\varvec{\tau }}}\cdot {\varvec{\tau }}^\text {PID}(t). \end{aligned}$$

Here, with the same reasoning as in (22), the use of the PID controller output—i.e. its D-part—in the transformation, brings along a transformation of the desired torque rate ${\dot{{\varvec{\tau }}}}^\text {des}$ to $\dot{{{\varvec{\theta }}}}^\text {des}$. Hence, it is not needed for the conceptional layer to feed a separate signal ${\dot{{\varvec{\theta }}}}^\text {des}$ into the PID controllers (16) and (24) in the transformational layer (${\dot{{\varvec{\theta }}}}^\text {err}={\dot{{\varvec{\theta }}}}$, i.e. ${\dot{{\varvec{\theta }}}}^\text {des}$ is used), yet, it would be possible in a simple way.

Using the signal of desired joint angles (42), the torque control concept (37) can be applied to the musculo-skeletal system, closing the hierarchical control loop.

The complete hierarchical control architecture, including the direct $\theta$-controller, the hierarchical $\theta _\lambda$-controller, and the respective layer transformations, is displayed in Fig. 4. The proposed control architecture is consistent with the structural hybrid control concept (Bayer et al. 2017; Kistemaker et al. 2006). It shifts the control space to a conceptional layer and, by making use of Jacobian-based layer transformations, it is capable of providing the required input signals of desired CE lengths ${\varvec{\lambda }}(t)$ and ‘open-loop’ stimulations ${\varvec{u}}^\text {opn}(t)$ to the hybrid controller. Due to the closed-form of the Jacobian matrices used in the transformations, the anatomical and active tissue knowledge (moment arms and stiffnesses), which is used to resolve the muscle redundancy, becomes apparent. The resolved redundancy furthermore opens a manifold to satisfy additional criteria, such as a joint-based co-contraction, along with the task execution.

## Model simulations

To demonstrate the function of the hierarchical control architecture (Sec. 2.2), it is here used to perform human-like upright stance by implementing a joint-torque-based control concept (Günther and Wagner 2016) on the musculo-skeletal model described in Sect. 2.1. Günther and Wagner (2016) proposed a control concept, which determines joint torques that stabilise the mechanical system (1) around an upright position. This control concept is implemented in the conceptional layer and feeds its output to the hierarchical control architecture to generate MTU stimulations that eventually lead to the desired joint torques demanded by the stabilising torque-based control concept.

This approach is used in a total of four simulations to demonstrate the control behaviour for the task of upright stance; of an unperturbed system (Sect. 3.2); with added noise perturbation (Sect. 3.3); with the additional criteria of joint-based co-contraction (Sect. 3.4); and with the synthesisation of a squat movement (Sect. 3.5), which is basically an additional control objective added to the balancing task. Details on the simulation software and the used solver are presented in Appendix E.

**Table 1:** Initial conditions and control parameters used for the computational synthesisation of quiet upright stance with the hierarchical distributed PID-control laws

	Conceptional control	${\varvec{\tau }}$-Controller			$\theta _\lambda$-Controller			$\theta$-Controller			Initial conditions
Joint											
Lu AA	–	–	–	–	0.01	0.01	0.001	0.1	0.1	0.01	0
Lu FE	–	–	–	–	0.01	0.01	0.001	0.1	0.1	0.01	0
Ce AA	–	–	–	–	0.01	0.01	0.001	0.1	0.1	0.01	0
Ce FE	–	–	–	–	0.01	0.01	0.001	0.1	0.1	0.01	0
Sh AA (l/r)	–	–	–	–	0.01	0.01	0.001	0.1	0.1	0.01	0
Sh FE (l/r)	–	–	–	–	0.01	0.01	0.001	0.1	0.1	0.01	0
Eb FE (l/r)	–	–	–	–	0.01	0.01	0.001	0.1	0.1	0.01	0
Hp AA (l/r)	–	–	–	–	0.01	0.0	0.002	0.05	0.0	0.01	0
Hp FE (l/r)	172	2.0	30	0.0	1.5	1.0	0.1	1.5	1.0	0.1	0
Kn FE (l/r)	467	0.75	15	0.0	0.25	1.0	0.0035	0.25	1.0	0.0035	5
An FE (l/r)	784	3.0	50	0.0	0.5	1.0	0.0035	0.5	1.0	0.0035	$-5$

The conceptional joint-torque controllers are only active for the lower limb DoFs. For the conceptional plan of desired joint torques the additional position-based parameter $g_\text {{Hp},l/r}=-400~\text {N}$ is used [see (43)] with the set-point for the COM of $x_\text {COM,0}=2.86~\text {cm}$ anterior to the mean ankle joint position. For the structural control law, for each of the *k* MTUs, the control parameters $p_{\lambda ,k}={1.0}/{l^{\mathrm {CE}}_{\text {opt},k}}$ and $d_{\lambda ,k}=0$ are chosen. As an additional initial condition, the pelvis body is positioned at $X_P(t=0)=[0\;0\;0.9954]^T$, such that the feet are placed at the ground contacts. The initial activations of the MTUs are in steady state with $a_k=0.1$ for each of the $k=1\ldots 36$ MTUs, corresponding to the co-contraction that is parameterised by $\zeta ^u_k=\zeta ^u=0.1$ and $\zeta ^\theta _j=\zeta ^\theta =0.0$

### Conceptional layer formulation of the desired task

In a nutshell, Günther and Wagner (2016) proposed to approximate human upright stance as a three-segment inverted pendulum model. With this assumption they were able to predict that upright stance could be stabilised by combining joint-level stiffnesses in the An, Kn, and Hp joint with an active positional contribution based on the weighted deviation of the body’s centre of mass (COM) in the sagittal plane. This leads to the following equations for the desired joint torques to be implemented in (37) (Günther and Wagner 2016):

43 $$\begin{aligned} \begin{aligned} {\varvec{\tau }}^\text {des}_\text {l/r}(t)=&\begin{bmatrix} k_\text {Hp,FE,l/r}&{}0&{}0\\ 0&{}k_\text {Kn,l/r}&{}0\\ 0&{}0&{}k_\text {An,l/r} \end{bmatrix}\\&\cdot \begin{bmatrix} ( \theta _\text {Hp,FE,l/r}(t) - \theta _\text {Hp,FE,l/r,0})\\ ( \theta _\text {Kn,l/r}(t) - \theta _\text {Kn,l/r,0})\\ ( \theta _\text {An,l/r}(t) - \theta _\text {An,l/r,0}) \end{bmatrix}\\&+ \begin{bmatrix} g_\text {Hp,l/r}\cdot (x_{\text {COM}}(t)-x_\text {COM}^\text {des}(t))\\ 0\\ 0 \end{bmatrix}, \end{aligned} \end{aligned}$$

with $x_\text {COM}$ and $x_\text {COM}^\text {des}:=\frac{x_\text {An,l}(t)+x_\text {An,r}(t)}{2}+{x}_ \text {COM,0}$ being the actual and desired positions of the body’s COM in the direction of the *x*-axis, respectively, $\theta _{i,0}$ being the nominal angles for the left and right (index $\text {l/r}$) Hp, Kn and An FE joints and $k_i$ being the respective single-joint stiffness gains. These desired torques ${\varvec{\tau }}^\text {des}$ (43) allow to calculate the input to the hierarchical control architecture: ${\varvec{\tau }}^\text {des}$ is compared to the actual joint torques ${\varvec{\tau }}^\text {MTU}_{\delta _\tau }$ generated by the muscles to calculate the torque error ${\varvec{\tau }}^\text {err}_{{\varvec{\delta _\tau }}}$ (38), which is the input to the torque control law (39). The resulting controller output is then transformed via the torque–angle Jacobian matrix ${J}^{{\varvec{\theta }}{\varvec{\tau }}}$ (40, 41) to obtain a vector of desired of joint angles ${\varvec{\theta }}^\text {des}_\tau$ with (42) (postural plan). As this conceptional controller is only used to actuate the lower limb flexion–extension (FE) DoFs, the remaining DoFs of the full-body model—left and right (l/r) cervical joints (Ces), shoulders (Shs), elbows (Ebs), lumbar joints (Lus), and hip (Hp) abduction–adduction (AA) DoFs—are controlled by setting the postural plan directly to ${\varvec{\theta }}^\text {des}_j=0$ ($j\in [\text {{Ce}}_\text {l/r}, \text {{Sh}}_{\text {{FE},l/r}}, \text {{Sh}}_{\text {{AA},l/r}}, \text {{Eb}}_\text {l/r}, \text {{L}}_\text {l/r}, \text {{Hp}}_{\text {{AA},l/r}}]$). The completely filled vector of desired joint angles is then fed into the hierarchical $\theta _\lambda$-controller (16) and the direct $\theta$-controller (24), respectively, to generate the stimulation signal ${\varvec{u}}(t)={\varvec{u}}^{\theta \lambda }(t)+{\varvec{u}}^\theta +{\varvec{u}}^\text {coc}_\text {ref}+{\varvec{u}}^\text {coc}_\theta$ according to equation (14).

### Simulation task: quiet upright stance

In the first simulation study of this paper, the task of upright stance is performed by conceptional planning and structural execution using the presented hierarchical control architecture (see Sect. 2.2) to drive the full-body model (Sect. 2.1) by means of MTU stimulations. To this end, the torque–angle characteristic (43) is straightforwardly used in the conceptional layer (37) (see also Fig. 4). The parameter values are chosen such that the control concept (43) is symmetric on the left (index l) and the right (index r) body side. The joint stiffness gains $k_i$ for the lower-limb FE DoFs in (43) are chosen—in accordance to (Günther and Wagner 2016) (‘TIP(12&23&34)’)—to be the critical single-joint stiffnesses, calculated by $k_i=m_i\cdot g \cdot h_i$, where $m_i$ is the total mass above the respective joint, *g* is the gravitational acceleration and $h_i$ is the distance of the system’s COM above the respective joint (Günther and Wagner 2016). The numerical values of these stiffness gains $k_i$ for the model used in this study, as well as the chosen value for the positional weight $g_\text {{Hp},l/r}$, are listed in Table 1. The resulting joint torques are calculated w.r.t. the nominal joint angle configuration of $\theta _{\text {Hp,l/r},0}=0^\circ$, $\theta _{\text {Kn,l/r},0}=5^\circ$ and $\theta _{\text {An,l/r},0}=-5^\circ$. In accordance to this nominal joint angle configuration, the offset of the desired COM position is set to $x_\text {COM,0}=2.86\text {cm}$.

The PID control parameters for the conceptional ${\varvec{\tau }}$-controller, as well as for the hierarchical $\theta _\lambda$-controller and the direct $\theta$-controller are heuristically obtained and listed in Table 1. The control parameters for the low-level $\lambda$-controller are chosen to $p_{\lambda ,k}={1.0}/{l^{\mathrm {CE}}_{\text {opt},k}}$ and $d_{\lambda ,k}=0$ for all *k* MTUs. The sensor delays $\delta _\lambda$, $\delta _\theta$ and $\delta _\tau$ are all set to 0 to be in accordance with the original concept paper (Günther and Wagner 2016). The co-contraction parameters $\zeta ^u_k$ are chosen to the constant reference value of 0.1 for all muscles, and the parameters of the joint-based co-contraction are set to $\zeta ^\theta _j=0$ for all joints.

The results of this simulation are presented in Sect. 4.1.

### Simulation task: muscle noise perturbation

To test the robustness of the control architecture to noise, in a second simulation, the MTUs’ stimulation signals ${\varvec{u}}(t)$ are perturbed by a random noise signal. The control parameters and the initial conditions are hereby the same as in the unperturbed system (see Sect. 3.2) and are listed in Table 1.

At every tenth full integration time-step—which corresponds to a noise frequency of $100~\text {Hz}$, based on the maximum integration step size of $0.001~\text {s}$ (see Appendix E)—for each of the $k=1\ldots {n_\text {MTU}}$ MTUs, a uniformly-distributed random noise-coefficient $p^\text {rnd}_k$ in the half-open interval $(-0.5,\;0.5]$ is calculated. The total stimulation signal with added noise perturbation $P^\text {rnd}=\text {diag}(p^\text {rnd}_1,\,\ldots ,\,p^\text {rnd}_{n_\text {MTU}}) \in {\mathbb {R}}^{{n_\text {MTU}}\times {n_\text {MTU}}}$ that is applied to the system then follows:

44 $$\begin{aligned} {\varvec{u}}^\text {noise}(t)=(I_{n_\text {MTU}}+P^\text {rnd}(t))\cdot {\varvec{u}}(t), \end{aligned}$$

i.e. the noise addition may perturb the MTUs’ stimulations up to $50\%$ of their current value ($0.5\cdot u_k<u^\text {noise}_k\le 1.5\cdot u_k$ for the *k*-th MTU). This noisy stimulation signal (44) is the input to the activation dynamics (Eq. (56) in Appendix A.2), which inherently act as a low-pass filters before MTU activities are applied to the force-generating CEs. Based on the activation dynamics time-constant of $M_\text {H}=11.3$, the respective magnitude gain corresponds to $-19~\text {dB}$ for the frequency of $100~\text {Hz}$, at which the noise signal is updated.

The resulting control behaviour for this noise addition is presented in Sect. 4.2.

### Simulation task: joint-based co-contraction

Due to the redundant nature of the many MTUs acting on the fewer joints (DoFs), it is possible to utilise the therefrom arising (uncontrolled) manifold of control DoFs to fulfil joint-based co-contraction constraints as described in Sect. 2.2.2. Testing this is the purpose of this third simulation task.

Joint-based co-contraction is achieved by adding contributions ${\varvec{u}}^\text {coc}_\theta$ to the MTUs’ stimulation signals, of all MTUs acting on the same joint, exactly without interfering with the movement task of upright stance. As outlined in Sect. 2.2.2, such a stimulation contribution is obtained from the null-space of the pseudo-inverse of the angle–stimulation Jacobian ${J}^{{\varvec{u}}{\varvec{\theta }}}$.

Two simulations are performed to examine the effects of setting the joint co-contraction parameters $\zeta ^\theta _j$ for the left and right (index $\text {l}/\text {r}$) Hp FE, Hp AA, Kn and An joints ($j\in [\text {Hp}_{\text {{FE}}\text {l}/\text {r}},\, \text {Hp}_{\text {{AA}}\text {l}/\text {r}},\,\text {Kn}_{\text {l}/\text {r}}, \,\text {An}_{\text {l}/\text {r}}]$). One Simulation with an increased joint-based co-contraction value of $\zeta _j^\theta =\zeta ^\theta =0.2$ and one with a reduced value of $\zeta _j^\theta =\zeta ^\theta =-0.05$, for all of the *j* lower limb joints, respectively. All other model and controller parameters remain the same as for the first simulation study from Sect. 3.2 and are listed in Table 1.

The model behaviour for these variations on joint-based co-contraction compared to the default ($\zeta \theta =0.0$) is given in Sect. 4.3.

### Simulation task: squat movement

To demonstrate the flexibility of the presented hierarchical control architecture, a fourth and final simulation study was performed, which adds a squat movement on top of the free-balance controller. To achieve this squat movement, only changes in the conceptional layer are needed. More precisely, instead of having constant values of $\theta _{\text {Hp,l/r},0}$, $\theta _{\text {Kn,l/r},0}$, and $\theta _{\text {An,l/r},0}$ for the nominal Hp, Kn, and An FE angles (43), a linear change over time of these nominal angles is employed. To let the position of the pelvis body in *x*-direction remain approximately constant during the squat movement, the nominal Kn and Hp FE angles were chosen (trigonometrically) dependent on the nominal An angle:

45 $$\begin{aligned} \theta _\text {An,l/r,0}(t)= & {} \left\{ \begin{array}{ll} -5^\circ &{} t\le t^*\\ -5^\circ -\frac{\theta _\text {An}^*}{\varDelta t}\cdot (t-t^*)&{}t> t^*\\ -5^\circ -\theta _\text {An}^*&{}\;\text {for}\; t> t^*+\varDelta t\\ -5^\circ -\theta _\text {An}^*\cdot \left( 1+\frac{t-(t^*+2\varDelta t)}{\varDelta t}\right) &{}t> t^*+2\varDelta t\\ -5^\circ &{} t> t^*+3\varDelta t,\\ \end{array} \right. \nonumber \\ \end{aligned}$$

46 $$\begin{aligned} \theta _\text {Kn,l/r,0}(t)= & {} \sin ^{-1}\left( -\frac{L_\text {S}}{L_\text {T}} \cdot \sin (\theta _\text {An,l/r,0}(t))\right) \nonumber \\&\quad -\theta _\text {An,l/r,0}(t)-\left. \theta _\text {An,l/r,0}\right| _{t=0} \end{aligned}$$

47 $$\begin{aligned} \theta _\text {Hp,l/r,0}(t)= & {} -\theta _\text {Kn,l/r,0}(t)-\theta _\text {An,l/r,0}(t), \end{aligned}$$

where $L_\text {T}$ and $L_\text {S}$ are the segment lengths of the thigh (T) and shank (S) bodies, respectively (see *supplementary material*) , $\theta _\text {An}^*$ is the final An angle offset of the squat, $t^{*}$ is the time instance initiating the squat and $\varDelta t$ is the interval for each of the three squat phases, i.e. the downwards movement, the squat stance and the upwards movement. With this simple linear approach, two squat movements have been synthesised *in-silico*; a slow squat ($t^*=10~\text {s}$, $\varDelta t=5~\text {s}$, $\theta _\text {An}^*=-15^\circ$) and a fast squat ($t^*=16.75~\text {s}$, $\varDelta t=0.5~\text {s}$, $\theta _\text {An}^*=-10^\circ$).

Beside these changes to the nominal angles, the single joint stiffnesses $k_i$ had to be doubled, i.e. $k_i^\text {sqt}=2\cdot k_i$, for the left and right Hp, Kn and An FE joints. The remaining parameters are exactly the same as for the first simulation study from Sect. 3.2 and are listed in Table 1. The results for these parameter manipulations, including joint angle trajectories, MTU force production, and an impression on the state-dependent redundancy solution by the angle–length Jacobian ${J}^{{\varvec{\lambda }}{\varvec{\theta }}}$, are given in Sect. 4.4.

**Fig. 5:**
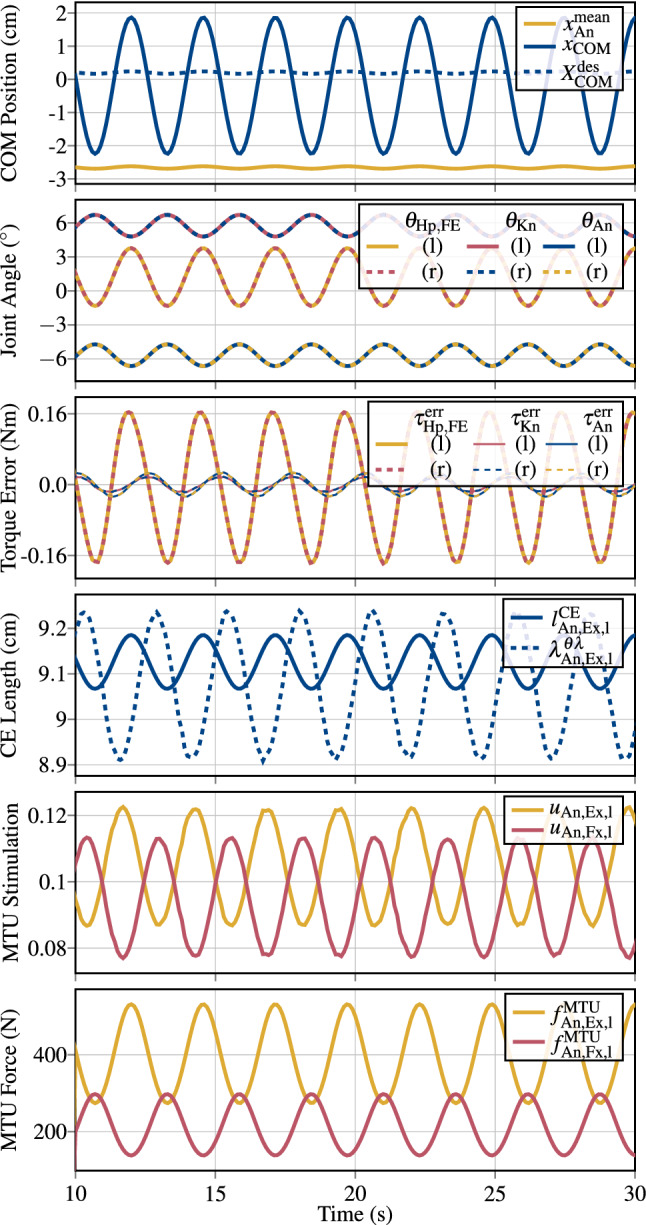
Simulation results of quiet upright stance. From top to bottom the trajectories of the body’s COM, the joint angle sway of left and right (l/r) Hp, Kn and An FE joints, the respective control errors on joint torques, the control error of CE lengths for the An Ex, the resulting MTU stimulations, and forces for the An Ex and Fx MTUs are displayed. The body’s COM position is anterior to the ankle joints at all times (Smith 1957)

## Results

### Simulation results: quiet upright stance

The here proposed control architecture allowed to stabilise free upright stance in a muscle-driven full-body model based on the idealised three-segment torque-driven concept (43) proposed by Günther and Wagner (2016). A stable attractor was found with the parameter values for the conceptional layer taken from the original concept paper (Günther and Wagner 2016) (‘TIP(12&23&34)’) and all other parameters set, as described in Sect. 3.2 above (see also Table 1).

For the time from $10~\text {s}$ to $30~\text {s}$, the COMs positional error, the lower-limb joint angles, the (high-level) control error of joint torques, the actual and desired (as demanded by the transformational layer) CE lengths (of the An Ex), as well as signals of the generated MTU stimulations and MTU forces (of the An Fx and Ex), are shown in Fig. 5. During this time interval, the maximum positional error in the sagittal plane of the body’s COM w.r.t. it’s desired set-point $x^\text {des}_\text {COM}(t)$ (see (43) and Table 1) is about $2.5~\text {cm}$. The maximum joint angle sway is about $5^\circ$ in Hp FE and $1.9^\circ$ in An and Kn FE. Based on the used convention of joint rotations (see Fig. 3), the Kn and the An joints show in-phase behaviour, where the Hp FE joint angle is in anti-phase with the other joints of the lower limbs, with a main oscillation frequency of $0.38~\text {Hz}$. The MTU stimulations vary around the reference co-contraction value of $u^\text {coc}_\text {ref}=0.1$. Compared to this constant co-contraction value, the An Ex stimulation is inhibited with a maximum reduction of about $0.13\%$ and the An Ex is stimulated up to an additional $22.5\%s$. These stimulation signals stabilise the mechanical system of the triple inverted pendulum and are based on the conceptional layer controller for joint torques. The absolute control error on joint torques has a maximal value of $\pm 0.17~\text {Nm}$ in the Hp joint, and $\pm 0.017~\text {Nm}$, and $\pm 0.027~\text {Nm}$ in the Kn and An joints, respectively. Compared to the maximal absolute values of MTU generated torques of $\vert {\varvec{\tau }}^\text {MTU}_\text {max, Hp}\vert =5.84~\text {Nm}$, $\vert {\varvec{\tau }}^\text {MTU}_\text {max, Kn}\vert =13.9~\text {Nm}$ and $\vert {\varvec{\tau }}^\text {MTU}_\text {max, An}\vert =22.3~\text {Nm}$, the relative control errors correspond to an estimated maximal deviation of about $2.9\%$ for the Hp, $0.2\%$ for the Kn and $<0.1\%$ for the An joint. The hierarchical control architecture transforms this error signal firstly to the transformational (joint) layer and subsequently to the structural (muscle) layer and, thus, produces desired muscle lengths ${\varvec{\lambda }}^\theta$, which are controlled by the $\lambda$-controller (11). The maximal error of the actual CE length $l^{\mathrm{CE}}_{\text {{An}}, \text {{Ex}}}$ of the Hp Fx muscle An Ex MTU and the desired value $\lambda ^\theta _{\text {{An}}, \text {{Ex}}}$ occurs shortly before the maximal positive body sway is reached. The shape of the MTU forces of the An FE joint is in accordance with the variations in MTU stimulation and CE length error. The An Ex oscillates around $369~\text {N}$ with a peak-to-peak amplitude of $256~\text {N}$, the An Fx around $213~\text {N}$ with peak-to-peak amplitude of $160~\text {N}$.

**Fig. 6:**
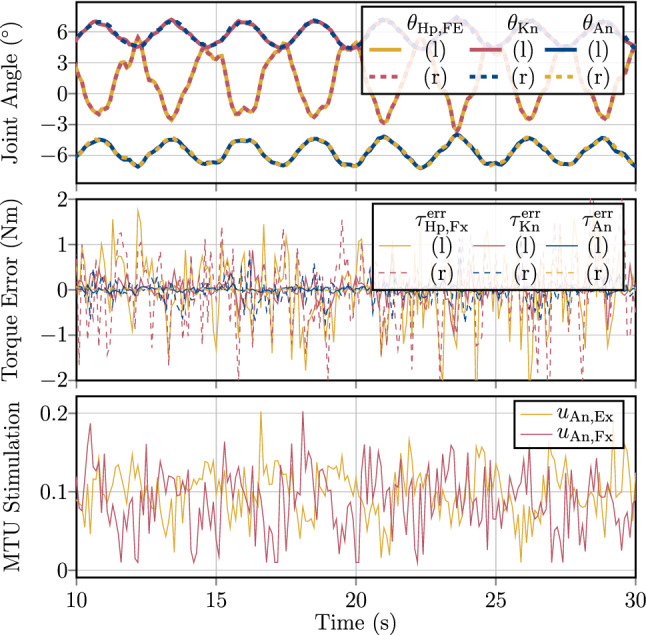
Simulation results of quiet upright stance with noise perturbation. The noise addition may perturb the individual MTU stimulation signal by up to $50\%$ of their current value (44). Due to this, the generated joint torques eventually exhibit noisy behaviour as well, leading to a ‘re-amplification’ of the noise in the MTU stimulations, mainly by ${P}_\tau$ and ${D}_\tau$ in the closed-loop, torque-controlled system. In spite of the huge noise in the MTU stimulations and joint torques, the joint angle trajectories are barely affected by it

**Fig. 7:**
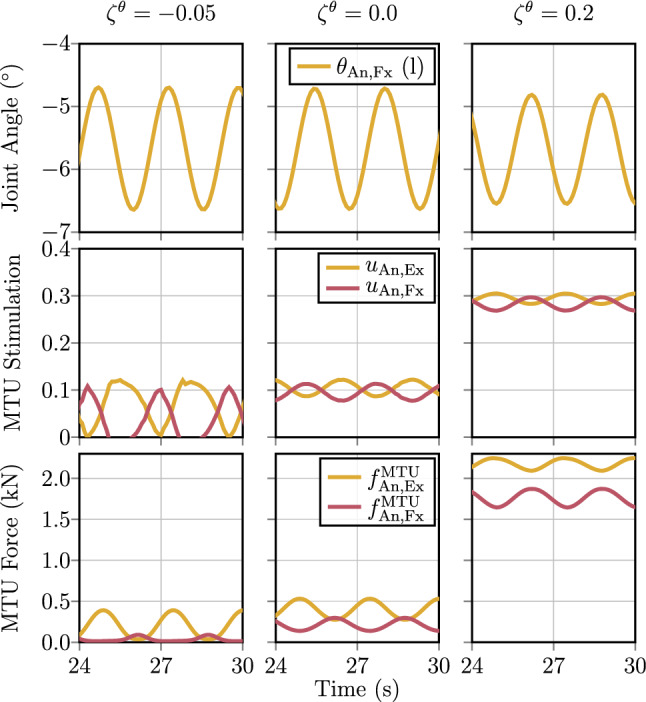
Simulation results of quiet upright stance with joint-based co-contraction variations (see Sects. 3.4 and 4.3 ). The middle graphs show the default ($\zeta ^\theta _j=\zeta ^\theta =0.0$), where in the left column a joint-based co-contraction of $\zeta ^\theta _j=\zeta ^\theta =-0.05$ in all leg joints ($j\in [\text {{Hp}}_\text {{FE},l/r},\,\text {{Hp}}_\text {{AA},l/r},\,\text {{Kn}}_\text {l/r},\,\text {{An}}_\text {l/r}]$) is used, and $\zeta ^\theta _j=\zeta ^\theta =0.2$ in the right column. In both variations, the MTU stimulations are adjusted according to $\zeta ^\theta$ and so are the respective MTU forces. The joint-based co-contraction is hereby based on the null-space of the Jacobian transformations. Due to the approximations of the associated angle–stimulation Jacobian ${J}^{{\varvec{u}}{\varvec{\theta }}}$ (25), the resulting joint trajectories are slightly different

### Simulation results: muscle noise perturbation

With the added noise perturbation described in Sect. 3.3, the closed-loop control architecture is still able to maintain upright stance. For the time interval from $10~\text {s}$ to $30~\text {s}$, the resulting joint angle trajectories, the control error of joint torques, and the stimulation signals of the Hp Fx and Ex muscles are shown in Fig. 6. In comparison to the large noise addition to the MTUs’ stimulations (up to $50\%$), the resulting influence on the joint angle trajectories is very small; more precisely (and after signal phase shifting), in the time interval $t\in [10~\text {s}\;30~\text {s}]$, the root-mean-square of the difference between the unperturbed and the noise-perturbed trajectory of the Hp FE angle is $1.28^\circ$. Even with the low-pass properties of the activation dynamics ($-19~\text {dB}$, see Sect. 3.3), the added noise perturbation influences the generated torques and the respective control error of joint torques. Compared—in the time interval $t\in [10~\text {s}\;30~\text {s}]$—to the unperturbed system that shows a root-mean-square of the Hp FE joint-torque control-error ${\varvec{\tau }}^\text {err}_\text {Hp,FE,l/r}$ of $0.148~\text {Nm}$, the noise perturbation increases this value to $0.747~\text {Nm}$, which is about 5 times higher.

### Simulation results: joint-based co-contraction

The results of the third simulation study substantiate the idea that the same task can be fulfilled under additional criteria of variations in joint-based co-contractions. More concretely, by exploiting the non-uniqueness of the Jacobian-based redundancy solution, a (uncontrolled) manifold of open DoFs can be found to adjust the stimulation level of all muscles acting on the same joint, without interfering with the execution of the task (see also Sects. 2.2.2 and 3.4 ). The results of variations in leg-joint co-contraction of $\zeta ^\theta _j=-0.05$ (left picture) and $\zeta ^\theta _j=0.2$ (right picture) are compared to a simulation without joint-based co-contraction ($\zeta ^\theta _j=0$) in Fig. 7 for the time interval from $10~\text {s}$ to $30~\text {s}$.

It can be seen that the MTUs’ stimulations of the Hp Fx and Ex are adapted accordingly by the joint co-contraction parameter, while upright stance is maintained. The An FE joint angle trajectories are similar for the different simulations, but slightly vary in amplitude and phase. This suggests that the null-space projection $0\overset{!}{=}\partial {\varvec{\theta }}={{J}^{{\varvec{u}}{\varvec{\theta }}}}^\dagger \partial {\varvec{u}}$ (34) behaves as intended within the presented hierarchical control architecture.

### Simulation results: squat movement

**Fig. 8:**
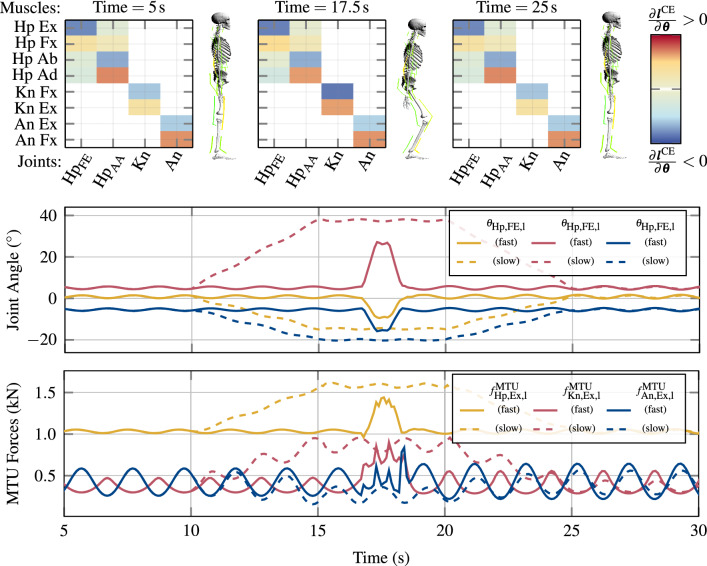
Simulation results of the fast ($\varDelta t=0.5~\text {s}$, solid lines) and slow ($\varDelta t=5~\text {s}$, dashed lines) squat movements according to the simulation task formulated in Sect. 3.5. The joint angles (upper line-plot) mostly follow their nominal trajectories (45–47) to execute the squat. The MTU forces of the Hp, Fx, and Kn Fx MTUs increase during the squat, while the An and Fx MTU force slightly decreases. This may be explained by the facts that the Hp and Kn joints swing further away from their unstable upright equilibria and the overall distance in *z*-direction of the body’s COM to the An joint decreases. In the box-plots, excerpts of the angle–length Jacobian matrix (18) for the left leg for the time instances $t=5~\text {s}$, $t=17.5~\text {s}$ and $t=25~\text {s}$ are displayed as heat-maps of MTU contribution to Fx and Ex movements

By changing the nominal angles and adjusting the single-joint stiffness gains $k_i$ in the conceptional joint-torque characteristic (43), the model was capable of executing a stable, unsupported squat movement.

The joint angle trajectories mostly follow their linear set values from (47, 46, 45) (see Fig. 8) and stable stance is maintained throughout the downwards movement, the squat stand and the stand-up phase for both, the slow and the fast squat. Additionally, in Fig. 8, excerpts of the angle–length Jacobian ${J}^{{\varvec{\lambda }}{\varvec{\theta }}}$ (17,18) are shown. This state-dependent matrix maps joint angle changes to CE length changes and thereby resolves the redundancy of the musculo-skeletal system. Based on the sign of the respective matrix entry, the kinesiologic effect of a certain muscle on a specific joint (flexor/extensor) can be identified (see colour coding on the right and joint angle notation in Fig. 3). As expected, the angle–length Jacobian is similar in all three states as it captures the kinesiological effect of the MTUs, which is a morphological feature and does not change. However, in the squat-position ($t=17.5$ s), Kn Ex and Fx entries for the Kn joint have higher magnitudes, which indicates a higher sensitivity of changes in the Kn MTUs’ CE lengths w.r.t. Kn joint angle changes.

## Discussion

All in all, the presented hierarchical control architecture showed to be a practical approach to actuate the used full-body model in the example of quiet upright stance. Moreover, due to the hierarchical architecture, only a few intuitive changes in the conceptional layer led to the synthesisation of a muscle-actuated, stable squat movement.

Due to the separation of the control system into a low-dimensional planning space (conceptional layer) and a high-dimensional actuation space (structural layer), the problem of movement planning was eased. That is, movement planning can occur in a conceptional context only, namely, by calculating required joint torques that are applicable to execute the movement for the mechanical system (1), without having to consider the properties of the biological actuators (muscles). The muscle actuation is achieved subsequently by Jacobian-based layer transformations that translate the conceptionally obtained movement plan to muscle stimulations, by using anatomical and tissue knowledge (muscle geometry and stiffnesses, respectively) to resolve the muscle redundancy. The Jacobian matrices that are used for this transformation are moreover presented here in closed-form, (based on the muscle model used). This gives structural insights into the calculations and the respective sensor signals that are needed for resolving the redundancy. Precisely since the redundancy resolution—which is carried out by Jacobian-based transformation from a high-dimensional into a lower-dimensional space—is not unique, additional DoFs (an uncontrolled manifold) remain that allow to fulfil additional criteria, such as joint-wide co-contraction, along—and not interfering—with the execution of the planned movement.

As the approach of conceptional (torque-based) planning is a common technique in the field of computational motor control (Günther and Wagner 2016; Rozendaal and van Soest 2005; Edwards 2007; Wolpert 1997), the presented control architecture forms a practical tool to validate such (torque-based) hypotheses on *in-silico*, muscle-actuated systems and therefore, to incorporate the effects of active and passive properties of the actuators. The results are promising regarding the application of the architecture to more dynamic and complex movements, with also the mechanical and muscular dimensionality enhanced to, for example, implementing bi-articular muscles or examining diverse body plans (animal morphologies). It seems conceivable to deploy the architecture as part of a movement system that also incorporates nonlinear dynamics of biological sensors, or let it be part of learning processes.

### Layered, hierarchical (biological) motor control

The present contribution assumes biological motor control as a layered, hierarchically structured system, as was already proposed by others (Wolpert 1997; Prescott et al. 1999; Todorov et al. 2005; DeWolf and Eliasmith 2011; Merel et al. 2019). Several observations hint in favour of this assumption. From our perspective, two layers are present, at least (Fig. 1): one on the lowest level—we call this ‘structural layer’—and one on the highest level—we call this ‘conceptual layer’.

The structural layer represents the direct wiring of both the efferent motor neurons from the spinal cord to the neuronal endplate and the afferent sensory neurons from the muscle receptor organs to the spinal cord, as well as the interconnections of these using one or very few synaptic connections (i. e. monosynaptic reflex loop). To date, no other mechanism seems accepted to transfer central nervous stimulations down to the active muscle tissue and, vice versa, from the muscle spindles and Golgi tendon organs up to the central nervous system (CNS).

The conceptual layer is an assumed, lumped layer representing the whole brain and complex spinal cord functions, including the crucial building blocks of motion planning. Among them are motor prediction (Wolpert and Flanagan 2001), haptic perceptions (Blakemore et al. 1999), motor learning (Tseng et al. 2007), movement intention (Ganesh et al. 2018), task and environment context (Wolpert et al. 2003), body representation (Naito et al. 2016), and sensory predictions (Gentili et al. 2010). In our example (Sect. 3), we assume the output of this layer to be a conceptual plan in the form of *local body representations*, i. e., a desired angle per each body joint plus *context*, i. e., co-contraction per each body joint (and reference stimulation levels). Any more sophisticated idea or model with the same output could be plugged in here, to replace the conceptual layer assumed by us.

In between the conceptional layer and the structural layer, we see the need to define one additional layer, the transformational layer. It serves one main function: reduction of cognitive load on higher CNS centres (brain and higher spinal cord). Cognitive load in this sense is, for example, total neuronal information communicated between the periphery and the brain (signal bandwidth), practical neuronal information necessary to complete motor tasks [control effort (Haeufle et al. 2014b)], and storage of knowledge about local material characteristics (e. g., tissue stiffness or membrane conductivity) and geometry (e.g. moment arms, limb dimensions). The transformation, as presented in this contribution, is done purely by exploiting the geometry and actuator characteristics, the morphology. That is, adding this layer reduces computational costs of higher CNS parts by increasing the morphological intelligence (Ghazi-Zahedi 2019) on lower to mid-levels of the control structure.

The transformational layer is separate from the structural layer: It synergises several distributed local control parts by using a more centralised knowledge about these parts. In turn, this centralisation enables time- and signal-efficient processing of sensory information close to the lowest level (structural layer). An additional argument in favour of this new layer is that common properties of the actual biological material characteristics have to be adjusted/learned only once, and can be stored in this layer and accessed from all other layers. In our presented approach, parameterisation of the transformational layer represents learning and storage of structural and material properties of the biological system.

### Biomechanical properties: towards a range of animals and movements

In the work of this paper, we aimed at formulating a model of a control architecture that can explain the synthesis of biological movement on the grounds of basic biomechanical and physiological properties of the biological structures involved, muscles in particular. We proceeded from the assumption that this architecture, which is rooted in and closely interacts with the biophysical properties of muscle-driven animals, may create a quite general, theoretical basis for explaining biological movement synthesis. This is, as our ansatz for the proposed biological architecture implies that it has evolved with some fundamental mechanical and physiological characteristics of the generic biological actuator (muscle) and its structural, lowest-level connection (mono-synaptic reflex pathway) to the controlling nervous system given, which are all inherent to at least the range of vertebrate animals. Our results provide first evidence that, accepting this ansatz as a starting point (evolutionary and as point of research departure), the very general process of planning and selecting any desired movement, and its control during realisation, may be facilitated by relying on such a given structural basis of the biological body moving.

We used the synthesis of three-dimensional human upright stance as a concrete example of a biological movement task, partly because it is a grand control challenge due to its inherent mechanical instability. This does not mean that this paper is about significantly advancing the detailed and precise comprehension of how this *particular* movement task is fulfilled. Yet, there is indeed a specific finding about human stance in the results presented in this paper. Namely, the whole control strategy to balance a human-like model in upright position is derived from another model (Günther and Wagner 2016) that is both remarkably more abstract and artless: a two-dimensional (sagittal plane) triple-inverted pendulum (TIP) model that is driven by solely elastic rotational springs in the three major (lumped) leg joints, with the only non-elastic addition of an active torque contribution in the hip joint [Eq. (43)]. In that model, therefore, the whole structural basis of biological movement generation, that is, muscles driven by stimulation signals, was not part of the strategy to find stable movement solutions. Thus, three major conclusions can be drawn at least: (i) One possible control strategy for balancing human upright stance is indeed well and concisely characterised by the ‘torque law’ Eq. (43) of a TIP. (ii) This seems to be a robust law as it formulates desired torques for only part of the body’s joints—although the crucial ones, the legs’—with neither knowledge on muscle, sensor, or neural properties, nor on the state of trunk or arms, nor on even the state in transversal direction or any torsions around the body’s longitudinal direction. Even more, our present implementation of a human-like neuro-musculo-skeletal model is in some respects still a pretty sloppy representation of human properties (see below). (iii) The points (i) and (ii) put together strongly indicate that a TIP’s Eq. (43) may be seen a ‘template’ (Full and Koditschek 1999), that is, a low-dimensional dynamics abstraction or condensation, respectively, of a movement system that may be made of many more degrees of freedom [higher-dimensional: an ‘anchor’ (Full and Koditschek 1999)], be the ‘anchor’ a model formulation or a system in the real, physical world. With the ‘template’ having imprinted the essence of the organisation of a dynamic movement task, the movement planning entities of an ‘anchor’ can then use the ‘template’ to suggest promising desired motor inputs (here: muscle stimulations) on the basis of predictions by the ‘template’.

Some parts of the present implementation of a human body model need either enhancement or upgrading, to make it appropriate for seriously examining the dynamic movement organisation of human stance, and quiet stance in particular. For example, the lever arms of the ankle muscles are 6 cm and 6.8 cm for the extensors and flexors, respectively, with particularly the latter being clearly higher than in real anatomy (about 4 cm). Also, whereas the (Hill) parameters $A_{\mathrm {rel}}=0.2$ and $B_{\mathrm {rel}} = 2$ $\hbox {s}^{-1}$ of the force-velocity relation are chosen well in the physiological range for all model muscles, the maximum isometric force values of the ankle flexors (3000 N) are over-estimated by about a factor of three [m. tibialis anterior: about 1000 N (Günther and Ruder 2003)], and the extensor’s value (likewise 3000 N) under-estimated (m. soleus plus m. gastrocnemius: about 6000 N). So far, bi-articular muscles have not been implemented, as reflex time delays have been neglected—albeit the present control architecture invites to model both biomechanically essential features. Furthermore, physiologically characteristic properties like the dynamics inherent to proprioceptive sensors, the muscle spindle and Golgi tendon organs, most sources of noise, and, just as a less prominent yet potentially interesting example, the distinction of long-range (CE force–length relation) stiffness from short-range stiffness contributions of the cross-bridges and filaments (Ford et al. 1977; Günther et al. 2018; Piazzesi and Lombardi 1995) have not been taken into account so far. It will be exciting to investigate how the present control architecture deals with them.

The specific simulation results of ‘quiet’ human stance presented here show fluctuation amplitudes of joint angles and horizontal COM position that are more than an order of magnitude higher than in measured data on quiet stance: compare Figs. 5,6,7 to (Günther et al. 2011, Tables 1,2) and (Günther et al. 2012, Table 1). In any case, an already realistically chosen value of the static coefficient of ground friction ($\mu _{c} = 0.8$) has guaranteed proper limitations of applicable joint torque peaks—and, therefore, horizontal ground reaction force fluctuations—in the current version of the human-like model. Together with the possible forces and their time rates due to viscoelastic fibre-tendon interaction already implemented in a physiologically realistic way, just like the muscle activation dynamics, the present model, even in the current state of development, rests well on sound biomechanical grounds. The control architecture laid out here is not restricted in any way to human movement. It rather waits for being applied to any vertebrate movement, with this, probed for scalability and validity, and certainly further moulded when exposed to interactions with other concepts or properties like, for example, biological sensor dynamics.

### Model and control considerations and limitations

We tested the control architecture for a full-body—but simplified—musculo-skeletal model. There are two important simplifications in the context of this study. The first simplification is the reduced number of muscles with only one antagonistic pair per mechanical degree of freedom and no bi-articular muscles. The second simplification is that each muscle is represented by only one motor unit (Haeufle et al. 2014a). Both simplifications reduce the redundancy in the model, i.e. we require only thirty-six muscle stimulation signals to control the full-body model instead of several thousands of $\alpha$-motoneuron signals in the real human body (de Luca and Contessa 2012). While the control architecture would allow to include more redundant and also bi-articular muscles, the possible benefit, e.g. for decoupling of parallel tasks (Latash 2012; Hsu and Scholz 2012), cannot be evaluated in the current model.

On the other hand, the model considers critical muscular nonlinearities and elasticities, which are expected to be difficult properties in the sense of control (Brändle et al. 2020) but central for understanding human movement control (van Soest and Bobbert 1993; Pinter et al. 2012; Stollenmaier et al. 2020). It is remarkable that, despite all the linear (Taylor) approximations in the Jacobians (17,26, 40) and the quasi-static assumptions for the relation between muscle-internal stiffnesses (76), the control architecture is able to handle these nonlinear elastic characteristics. For the tasks investigated here, upright stance and squat movements, also one set of PID control parameters was sufficient. While the heuristic tuning of these parameters was possible in this model, it may become difficult to infeasible for models with more muscles or for more complex or dynamic movements. This would potentially require a systematic optimisation of the control parameters. Furthermore, the stabilising effect of the control architecture on such a nonlinear model system cannot be guaranteed (yet) and therefore still relies on tests via simulations. However, stability has been mathematically proven in a simplified model (Brändle et al. 2020) for the structural layer and—from all simulations investigated here—seems practically achievable even in the hierarchical architecture.

### Supplementary Information

Below is the link to the electronic supplementary material.

Supplementary material 1 (pdf 1387 KB)

## Figures and Tables

**Fig. 9:**
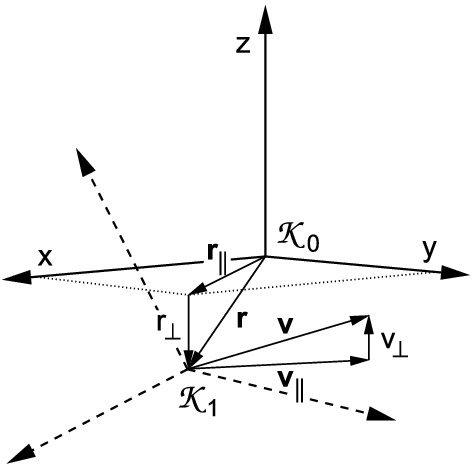
The symbol definitions for the contact situation. The coordinate system ${\mathcal {K}}_{0}$ (fixed to the ‘from’-body) represents the contact plane, and the origin of ${\mathcal {K}}_{1}$ (fixed to the ‘to’-body) the potentially contacting point. $\mathbf {r}$ and $\mathbf {v}$ symbolise position and velocity vectors, respectively. Indices ‘$\parallel$’ and ‘$\perp$’ symbolise a vector projection onto ${\mathcal {K}}_{0}$’s ${\mathsf {x}}$-${\mathsf {y}}$-plane and perpendicular to it (onto its ${\mathsf {z}}$-axis), respectively. Here, the ‘from’- and the ‘to’-body are indeed in contact with each other, as the position of the ${\mathcal {K}}_{1}$ origin is at ${\mathsf {z}}<0$ measured in the ${\mathcal {K}}_{0}$ system, i.e. below ${\mathcal {K}}_{0}$’s ${\mathsf {x}}$-${\mathsf {y}}$-plane intersecting at ${\mathsf {z}}$ = 0 (the contact plane). According to the principle of *actio* = *reactio*, the contact force vector ${\varvec{f}}$ (not shown here) is calculated as acting on the ‘from’-body (with ${\mathcal {K}}_{0}$) at the position of the point—the origin of ${\mathcal {K}}_{1}$—that contacts the plane, and the inverted force vector ($-{\varvec{f}}$) is set to act on the ‘to’-body (with ${\mathcal {K}}_{1}$) at the exact same position

**Fig. 10:**
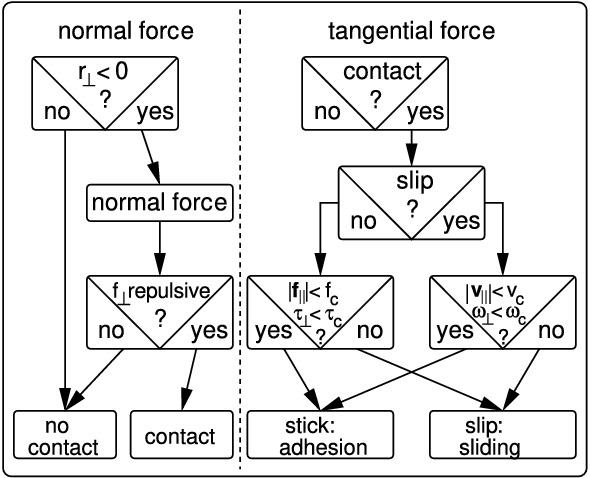
The states of a modelled point-to-plane (${\mathcal {K}}_{1}$–${\mathcal {K}}_{0}$) contact force interaction according to Fig. 9, and the transition criteria (decision scheme) for calculating the contact force vector ${\varvec{f}}$ (i.e. normal component $f_{\perp }$ > 0 and tangential components ${\varvec{f}}_{\parallel }$) in this ‘to’-body–‘from’-body interaction. The critical values of tangential (linear) and torsional (angular) velocities for slip-to-stick transitions, $\text {v}_{c}$ = 0.01 m $\hbox {s}^{-1}$ and $\omega _{c}$ = $\infty$ (with this, stick-to-slip disabled here), respectively, as well as for stick-to-slip transitions, tangential force and torsional (indicated by ‘$\perp$’) torque magnitudes, $f_{c}$ = $\mu _{c}$ $\cdot$ $f_{\perp }$ and $\tau _{c}$ = $\mu _{c \phi }$ $\cdot$ $f_{\perp }$, respectively, with the coefficient of static friction $\mu _{c}$ = 0.8 (stick-to-slip disabled, thus, $\mu _{c \phi }$ > 0 arbitrary), are parameters for modelling reversible transitions. The state of a contact interaction changes from ‘stick’ to ‘slip’ (based on $f_{c}$ and $\tau _{c}$), the ‘slip’ event, if *either* of the depicted criteria is *broken*, i.e. the transition to ‘slip’ occurs through a logical *or* connective of the ‘stick’ requirement. In contrast, the slip-to-stick transition (based on $\text {v}_{c}$ and $\omega _{c}$), the ‘stick’ event, occurs through a logical *and* connective of ‘back to stick’ requirement, i.e. *both* depicted criteria of approaching low velocities must be *fulfilled*. If there is no contact (anymore), the stick–slip state of a contact interaction is initialised to ‘slip’

**Fig. 11:**
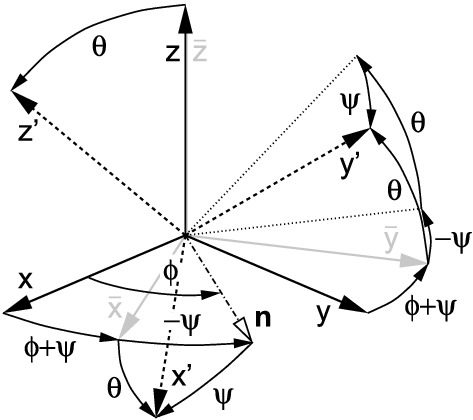
The calculation of the torsion angle $\xi = \phi + \psi$ and the stick coordinate system $\overline{{\mathcal {K}}}_1$ ($\overline{{\mathsf {x}}}$-$\overline{{\mathsf {y}}}$-$\overline{{\mathsf {z}}}$) at the instant of a ‘stick’ event: separating the tilt from the torsion contribution to an overall rotation of the primed coordinate system ${\mathcal {K}}_{1}$ (dashed axes) relative to the unprimed system ${\mathcal {K}}_{0}$ (solid axes). The rotations are expressed in terms of Euler angles executed in the order ${\phi ,\,\theta ,\,\psi }$. The tilt is the (second) angular rotation by $\theta$ around the ${\mathsf {n}}$-axis, the latter being the intermediate ${\mathsf {x}}$-axis after the first rotation around the initial (unprimed) ${\mathsf {z}}$-axis by $\phi$. The last (third) rotation by $\psi$ is then executed around the already finally fixed (primed) ${\mathsf {z}}'$-axis. The ${\mathsf {x}}$-${\mathsf {y}}$-plane of the (solid, unprimed) ${\mathcal {K}}_{0}$ system is the contact plane, in which to lie the ${\mathsf {n}}$-axis of tilting can be assumed and used for parameterising stick–slip interaction when applying the Euler angle description of three-dimensional rotations. Regardless of the angular orientation of ${\mathcal {K}}_{1}$ relative to ${\mathcal {K}}_{0}$, the position of the origin of ${\mathcal {K}}_{1}$ in relation to the contact plane determines (a) whether both coordinate systems (bodies) are in contact at all and, therefore, mechanically interact by a force $\mathbf {f}$ and a torque $\varvec{\tau }$, as well as (b) where in the contact plane a unique stick coordinate system $\overline{{\mathcal {K}}}_1$ is located for modelling a restoring viscoelastic tangential force and torsional torque in the ‘stick’ state. In fact, $\overline{{\mathcal {K}}}_1$ is fixed at the instant when the ‘stick’ event occurs: its origin is chosen to be located in the contact plane at the vector $\overline{\mathbf {r}}_{\parallel }$ of the projection of ${\mathcal {K}}_{1}$’s origin onto the contact plane—here, for simplicity of the illustration at ${\mathcal {K}}_{0}$’s origin—, the orientation of $\overline{{\mathcal {K}}}_1$’s $\overline{{\mathsf {z}}}$-axis is chosen to always align with the normal vector of the contact plane (${\mathcal {K}}_{0}$’s ${\mathsf {z}}$-axis), and the Euler angle sum ${\overline{\xi }} = {\overline{\phi }} + {\overline{\psi }}$ is calculated for determining later torsional angular excursions $\varDelta \xi = \xi - {\overline{\xi }}$ relative to the ‘stick’ condition

## Data Availability

The underlying data from the plots in Figs. 5–8 are available on request to the authors.

## Mathematical models

### Force characteristics of the Hill-type muscle model

The Hill-Type muscle model used in this study describes each of the $k=1\ldots {n_\text {MTU}}$ muscles as a deflected, one-dimensional, massless, string-like muscle–tendon unit (MTU) with total length $l^\text {MTU}_k$ [see also (3) and (Haeufle et al. 2014a)]. As depicted in Fig. 2 in Sect. 2.1b, each MTU consists of an active contractile element (CE) with length $l^{\mathrm{CE}}_k$, a parallel elastic element (PEE) (with CE length), a serial elastic element (SEE) with length $l^{\mathrm{SEE}}_k$ and a serial damping element (SDE) (with SEE length). With respect to the force equilibrium (4), each of those force producing elements is capable of generating a contribution to the pulling force $f^\text {MTU}_k$ of the *k*-th MTU that acts on the respective attachment and deflection points (Hammer et al. 2019). The exerted force of each element has nonlinear parametric dependencies, including one or more, of the CE length $l^{\mathrm{CE}}_k$, the SEE length $l^{\mathrm{SEE}}_k$ $(=l^\text {MTU}_k-l^{\mathrm{CE}}_k)$, the respective velocities ${\dot{l}}^{\mathrm{CE}}_k$, ${\dot{l}}^\text {SEE}_k$ and the CE’s activity $a_k$. It is notable that there are no parametric cross-dependencies between the single MTUs and, thus, each muscle can be modelled individually.

In the following subsections, a description of the force equations of the single elements are given, as well as of further calculations, which are, for example, part of the calculations of the Jacobian transformations.

#### Contractile element (CE)

The force–length relation a *k*-th CE follows a bell-shaped curve with an ascending branch (index $\text {limb}=\text {asc}$) and a descending branch (index $\text {limb}=\text {desc}$). The total force exerted by this *k*-th CE is dependent on the length $l^{\mathrm{CE}}_k$, the contraction velocity ${\dot{l}}^{\mathrm{CE}}_k$ and the activity $a_k$. It follows:

48 $$\begin{aligned} \begin{aligned}&f^{\mathrm{CE}}_k(l^{\mathrm{CE}}_k, {\dot{l}}^{\mathrm{CE}}_k,a_k)=f^\text {CE}_{\text {max},k}\\ {}&\quad \cdot \left( \frac{a_k\cdot f^\text {isom}_k(l^{\mathrm{CE}}_k)+A_{\text {rel},k}(l^{\mathrm{CE}}_k,a_k)}{1-\frac{{\dot{l}}^{\mathrm{CE}}_k}{B_{\text {rel},k}(l^{\mathrm{CE}}_k, a_k)\cdot l^{\mathrm {CE}}_{\text {opt},k}}}-A_{\text {rel},k}(l^{\mathrm{CE}}_k,a_k)\right) , \end{aligned}\nonumber \\ \end{aligned}$$

where the isometric force $f^\text {isom}_k(l^{\mathrm{CE}}_k)$ is given by an exponential function of the form

49 $$\begin{aligned} f^\text {isom}_k(l^{\mathrm{CE}}_k)=e^{\left\{ - \left| \frac{\frac{l^{\mathrm{CE}}_k}{l^{\mathrm {CE}}_{\mathrm {opt},k} }-1}{\varDelta W_{\mathrm {limb},k}}\right| ^{\nu ^{\mathrm {CE}}_{\mathrm {limb},k}} \right\} }. \end{aligned}$$

Details and physiological background of the used parameters (including the Hill parameters $A_{\text {rel},k}$ and $B_{\text {rel},k}$) are well described in Haeufle et al. (2014a).

Using these force equations, the change of the isometric force $f^\text {isom}_k$ with respect to a change of the CE length $l^{\mathrm{CE}}_k$ of the *k*-th MTU can be calculated analytically as

50 $$\begin{aligned} \frac{\partial f^\text {isom}_k}{\partial l^{\mathrm{CE}}_k}=\frac{\nu ^{\mathrm {CE}}_{\text {limb},k} \cdot f^\text {isom}_k\cdot (l^{\mathrm{CE}}_{\text {opt},k}-l^{\mathrm{CE}}_k)\left| \frac{(l^{\mathrm{CE}}_k/l^{\mathrm{CE}}_{\text {opt},k})-1}{\varDelta W^{\mathrm {CE}}_{\text {limb},k}}\right| ^{\nu ^{\mathrm {CE}}_{\text {limb},k}-2}}{{l^{\mathrm{CE}}_k}^2 \cdot \varDelta {W^{\mathrm {CE}}_{\text {limb},k}}^2}.\nonumber \\ \end{aligned}$$

#### Parallel elastic element (PEE)

The force of the PEE of the *k*-th MTU vanishes, as long as the CE length $l^{\mathrm{CE}}_k$ is below the threshold length $l^\text {PEE}_{0,k}$. When the MTU is stretched above this length, the exponential increasing force

51 $$\begin{aligned} f^{\mathrm{PEE}}_k(l^{\mathrm{CE}}_k)=\left\{ \begin{array}{ll} 0, &{} l^{\mathrm{CE}}_k<l^{\mathrm {PEE}}_{0,k} \\ k^\mathrm {PEE}_k(l^{\mathrm{CE}}_k-l^{\mathrm {PEE}}_{0,k})^{\nu ^\mathrm {PEE}_k}, &{} l^{\mathrm{CE}}_k\ge l^{\mathrm {PEE}}_{0,k} \end{array} \right. , \end{aligned}$$

with $k^\mathrm {PEE}_k=f^{\mathrm{PEE}}_k\frac{f^\text {CE}_{\mathrm {max},k}}{(l^{\mathrm{CE}}_{\text {opt},k}(\varDelta W_{\text {des},k}+1-{l}^{\mathrm {PEE}}_{0,k}))^{\nu ^\mathrm {PEE}_k}}$ arises. Its derivative with respect to $l^{\mathrm{CE}}_k$ is given by

52 $$\begin{aligned} \frac{\partial f^{\mathrm{PEE}}_k}{\partial l^{\mathrm{CE}}_k}=\left\{ \begin{array}{ll} 0, &{} l^{\mathrm{CE}}_k<l^{\mathrm {PEE}}_{0,k} \\ k^\mathrm {PEE}_k\cdot \nu ^\mathrm {PEE}_k \cdot (l^{\mathrm{CE}}_k-l^{\mathrm {PEE}}_{0,k})^{(\nu ^\mathrm {PEE}_k-1)}, &{} l^{\mathrm{CE}}_k\ge l^{\mathrm {PEE}}_{0,k} \end{array} \right. . \nonumber \\ \end{aligned}$$

#### Serial elastic element (SEE)

The SEE of the *k*-th MTU describes the force of the aponeuroses and the tendons that connect the muscle belly (CE) to the bones. Below its tendon slack length $l^{\mathrm{SEE}}_{0,k}$, the SEE force is zero ($f^{\mathrm{SEE}}_k=0$). Shortly after the transition ($l^{\mathrm{SEE}}_{0,k}<l^{\mathrm{SEE}}_k<l^{\mathrm{SEE}}_{\text {nll},k}$), the tendon force rises nonlinearly until the tendon length reaches $l^{\mathrm{SEE}}_{\text {nll},k}$. After that the force of the *k*-th SEE is linearly dependent on its length $l^{\mathrm{SEE}}_k$:

53 $$\begin{aligned} f^{\mathrm{SEE}}_k(l^{\mathrm{SEE}}_k)=\left\{ \begin{array}{ll} 0, &{} l^{\mathrm{SEE}}_k\le l^{\mathrm{SEE}}_{0,k} \\ k^{\mathrm {SEE}}_{\text {nl},k}(l^{\mathrm{SEE}}_k-l^{\mathrm{SEE}}_{0,k})^{\nu ^\mathrm {SEE}_k}, &{} l^{\mathrm{SEE}}_k<l^{\mathrm{SEE}}_{\text {nll},k} \\ \varDelta f^{\mathrm {SEE}}_{0,k}+k^{\mathrm {SEE}}_{l,k}(l^{\mathrm{SEE}}_k-l^{\mathrm{SEE}}_{\text {nll},k}), &{} l^{\mathrm{SEE}}_k\ge l^{\mathrm{SEE}}_{\text {nll},k} \end{array} \right. . \nonumber \\ \end{aligned}$$

The partial derivative of $f^{\mathrm{SEE}}_k$ with respect to $l^{\mathrm{SEE}}_k$ can be calculated to

54 $$\begin{aligned} \frac{\partial f^{\mathrm{SEE}}_k}{\partial l^{\mathrm{SEE}}_k}=\left\{ \begin{array}{ll} 0, &{} l^{\mathrm{SEE}}_k<l^{\mathrm{SEE}}_{0,k} \\ k^{\mathrm {SEE}}_{\text {nl},k}\cdot \nu ^\mathrm {SEE}_k\cdot (l^{\mathrm{SEE}}_k -l^{\mathrm{SEE}}_{0,k})^{\nu ^\mathrm {SEE}_k-1}, &{} l^{\mathrm{SEE}}_k<l^{\mathrm{SEE}}_{\text {nll},k} \\ k^{\mathrm {SEE}}_{\text {l},k} ,&{} l^{\mathrm{SEE}}_k\ge l^{\mathrm{SEE}}_{\text {nll},k} \end{array} \right. , \nonumber \\ \end{aligned}$$

#### Serial damping element (SDE)

In parallel to the SEE, a nonlinear damping element is part of the MTU model. The velocity-dependent force of the *k*-th SDE is given by

55 $$\begin{aligned}&f^\text {SDE}_k(l^{\mathrm{CE}}_k, {\dot{l}}^{\mathrm{CE}}_k, {\dot{l}}^{\mathrm{MTU}}_k)= \nonumber \\&\quad D^{\mathrm {SDE}}_{\mathrm {max}_k}\cdot \left( (1-R^\mathrm {SDE}_k) \frac{f^{\mathrm{CE}}_k(l^{\mathrm{CE}}_k, {\dot{l}}^{\mathrm{CE}}_k, a_k)+f^{\mathrm{PEE}}_k(l^{\mathrm{CE}}_k)}{f^\text {CE}_{\mathrm {max},k}} +R^\mathrm {SDE}_k\right) \nonumber \\&\quad \cdot \left( {\dot{l}}^{\mathrm{MTU}}_k-{\dot{l}}^{\mathrm{CE}}_k\right) , \end{aligned}$$

with $$D^\mathrm {SDE}_{\mathrm {max},k}=D^\mathrm {SDE}_k\cdot \frac{f^{\mathrm{CE}}_{\mathrm {max},k}\cdot A_{\mathrm {rel},k}}{l^{\mathrm{CE}}_{\text {opt},k}\cdot B_{\mathrm {rel},k}}$$.

### Activation dynamics

The electrochemical process of transforming the electrical neural signal to the chemical $\hbox {Ca}^{2+}$ ion concentration in the sarcoplasma of the muscle is described by a model of activation dynamics (Rockenfeller and Günther 2018), which is an enhanced version of what had originally been developed by Hatze (1977). All used parameter values are listed in Table 5 in the *supplementary material*.

Firstly, for the *k*-th MTU, the neural stimulation signal $u_k$ serves as input for a first-order differential equation with the state variable of the normalised $\hbox {Ca}^{2+}$ ion concentration $\gamma _k$.

56 $$\begin{aligned} {\dot{\gamma }}_k=M_{\mathrm {H}}(u_k-\gamma _k). \end{aligned}$$

In a second step, the state $a_k$ (activity) of the MTU is obtained by the nonlinear relationship

57 $$\begin{aligned} a_k(\gamma _k,l^{\mathrm{CE}}_k)=\frac{a_0+\vartheta _k(\gamma _k,l^{\mathrm{CE}}_k)}{1+\vartheta _k(\gamma _k,l^{\mathrm{CE}}_k)}, \end{aligned}$$

with $\vartheta _k=(\gamma _k\cdot \varpi _k(l^{\mathrm{CE}}_k))^\nu$, and $\varpi _k(l^{\mathrm{CE}}_k)=\varpi _{\text {opt},k}\cdot \frac{l^{\mathrm{CE}}_k}{{l_\text {opt}^\mathrm {CE}}_k} =\gamma _\mathrm {max}\cdot \rho _0\cdot \frac{l^{\mathrm{CE}}_k}{{l_\text {opt}^\mathrm {CE}}_k}$. The partial derivative of $a_k$ w.r.t $l^{\mathrm{CE}}_k$ therefore follows

58 $$\begin{aligned} \frac{\partial a_k}{\partial l^{\mathrm{CE}}_k}= & {} \frac{(1-a_0)\cdot \dfrac{\partial \vartheta _k}{\partial l^{\mathrm{CE}}_k}}{(1+\vartheta _k)^2}, \quad \text {with} \end{aligned}$$

59 $$\begin{aligned} \frac{\partial \vartheta _k}{\partial l^{\mathrm{CE}}_k}= & {} \nu \cdot \left( \frac{\gamma _k(t)\cdot \varpi _{\text {opt},k}}{{l_\text {opt}^\text {CE}}_k}\right) ^\nu \cdot {l^{\mathrm {CE} \;(\nu -1)}_k}=\frac{\nu \cdot \vartheta _k}{l^{\mathrm{CE}}_k}. \end{aligned}$$

For the derivative of $a_k$ w.r.t $\gamma _k$ the same steps are taken, leading to

60 $$\begin{aligned} \frac{\partial a_k}{\partial \gamma _k}=\frac{(1-a_0)\cdot \dfrac{\partial \vartheta _k}{\partial \gamma _k}}{(1+\vartheta _k)^2}, \quad \text {with}\quad \frac{\partial \vartheta _k}{\partial \gamma _k}=\frac{\nu \cdot \vartheta _k}{\gamma _k}. \end{aligned}$$

### Joint-limitation forces and viscoelastic forces

The (generalised) joint-limitation forces (torques) ${\varvec{\tau }}^\text {lmt}_j$ of the *j*-th rotational DoF ${q}_j$ are modelled as linear, one-sided, spring-damper elements that act directly on the respective DoF:

61 $$\begin{aligned} {\varvec{\tau }}^\text {lmt}_j=\left\{ \begin{matrix} k^\text {lmt}_{\text {l},j}\cdot ({q}_j-{q}_{\text {l},j})+d^\text {lmt}_{\text {l},j}\cdot {\dot{{q}}}_j&{} ,\,{q}_j < {q}_{\text {l},j}\\ 0 &{} ,\,{q}_{\text {l},j} \le {q}_j \le {q}_{\text {u},j} \\ k^\text {lmt}_{\text {u},j}\cdot ({q}_j-{q}_{\text {u},j})+d^\text {lmt}_{\text {u},j}\cdot {\dot{{q}}}_j &{} ,\,{q}_j> {q}_{\text {u},j} \end{matrix} \right. \nonumber \\ \end{aligned}$$

with the lower and upper (index $\text {l}/\text {u}$) threshold angles $q_{\text {l}/\text {u},j}$ and the respective linear spring and damping parameters $k^\text {lmt}_{\text {l}/\text {u},j}$ and $d^\text {lmt}_{\text {l}/\text {u},j}$.

The viscoelastic (bushing) forces $\tau ^\text {bsh}_j$, which are used as a description for passive viscoelastic tissue around the joint (e.g. ligaments), are modelled with the same force law in the special case of ${q}_{\text {l},j}={q}_{\text {u},j}=0$, $k^\text {bsh}_{\text {l},j}=k^\text {bsh}_{\text {u},j}=k^\text {bsh}_j$, and $d^\text {bsh}_{\text {l},j}=d^\text {bsh}_{\text {u},j}=d^\text {bsh}_{j}$. With this the viscoelastic force model for the *j*-th DoF contracts to

62 $$\begin{aligned} \tau ^\text {bsh}_j=k^\text {bsh}_{j}\cdot {q}_j+d^\text {bsh}_j \cdot {\dot{{q}}}_j. \end{aligned}$$

The parameters values for the joint limitations and the viscoelastic force element parameters are given in the *supplementary material*.

### Contact forces

In three-dimensional computer simulations, a common and most simple mathematical description of a contact between a body and a surface is to define a point fixed on the body, and approximate the surface by a plane. By then calculating, at any mechanical state of the body, the instantaneous distance between the point and the plane, a contact event between the point and the plane can be detected. In case of such an event, and with likewise knowing the movement relative to each other from the mechanical state, the force ${\varvec{f}}$ and torque $\varvec{\tau }$ acting on the body (here indicated as the ‘from’-body) can be modelled and calculated in terms of the body’s and the plane’s coordinates and velocities, as well as parameters describing the mechanical properties of the contacting and deforming materials. If the plane is fixed to another body (the ‘to’-body) , the parameters of point-to-plane contacts reflect lumped properties of both bodies, and the contact force and torque on the ‘from’-body simply act with inverted signs ($-{\varvec{f}}$ and $-\varvec{\tau }$) on the ‘to’-body, according to the principle of *actio* = *reactio*. Figure 9 illustrates such a contact situation: The contact plane is in the *demoa* (See Appendix E) implementation the ${\mathsf {x}}$-${\mathsf {y}}$-plane at ${\mathsf {z}}$ = 0 of the coordinate system ${\mathcal {K}}_{0}$ (on the ‘from’-body), and the origin of the coordinate system ${\mathcal {K}}_{1}$ (on the ‘to’-body) is the point continuously checked for contact with ${\mathcal {K}}_{0}$’s ${\mathsf {x}}$-${\mathsf {y}}$-plane. The vectors $\mathbf {r}$ and $\mathbf {v}$ of relative displacement and velocity, respectively, are depicted along with their respective projections onto ${\mathcal {K}}_{0}$’s ${\mathsf {x}}$-${\mathsf {y}}$-plane (indicated by ‘$\parallel$’) and ${\mathsf {z}}$-axis (indicated by ‘$\perp$’).

A two-dimensional (point-to-line) contact model approach formulated earlier (Günther 1997; Günther and Ruder 2003) has been used as a starting point for the three-dimensional contact model implementation in *demoa*. As body-body contacts in the real world always occur at finite surfaces, the three-dimensional *demoa* implementation gained a new part that takes, beyond the relative linear movement of the origins of ${\mathcal {K}}_{1}$ and ${\mathcal {K}}_{0}$, the angular movement of both contacting systems relative to each other into account. Accordingly, a contact torque $\varvec{\tau }$ is modelled in addition to the contact force ${\varvec{f}}$. The contact model in *demoa* discriminates, firstly, the conjugate contact model states ‘contact’ from ‘no contact’ by continuously identifying the position of ${\mathcal {K}}_{1}$’s origin along ${\mathcal {K}}_{0}$’s ${\mathsf {z}}$-axis (normal to the contact plane). Figure 10 gives a survey of the decision scheme to determine the overall state of a contact interaction. Secondly, it discriminates the conjugate states ‘stick’ from ‘slip’, for linear and angular movements separately, based on the tangential (linear) velocity of ${\mathcal {K}}_{1}$’s origin relative to ${\mathcal {K}}_{0}$’s origin and the torsional (angular) velocity of ${\mathcal {K}}_{1}$ relative to ${\mathcal {K}}_{0}$’s ${\mathsf {x}}$-${\mathsf {y}}$-plane (see Fig.11), respectively, as well as the modelled force acting tangentially to ${\mathcal {K}}_{0}$’s ${\mathsf {x}}$-${\mathsf {y}}$-plane and the torsional torque in this plane, respectively, with all state transitions being reversible.

To maximise the physical and mathematical consistency of detecting and handling both contact and stick–slip events while modelling multiple contact interactions, *demoa* offers deploying the well-tried *Shampine–Gordon* predictor-corrector algorithm ‘*de*’ (Shampine and Gordon 1975) for integrating a system of ordinary differential equations in a modified (Henze 2002) version. The modified ‘*de*’ algorithm identifies, by quadratic polynomial interpolation backwards in time for each event detected, the time order of such (contact and stick–slip) events that occur between the instant $t_{in}$ when ‘*de*’ is called with an initial state vector of the neuro-musculo-mechanical system, including all discrete contact and stick–slip states, and a user-fixed instant $t_{out}$ when ‘*de*’ is required to return an updated state vector that fulfils the likewise user-fixed absolute and relative accuracies. Events of reversible transitions between ‘contact’ and ‘no contact’ as well as between ‘stick’ and ‘slip’ are detected, and the discrete state values accordingly and properly switched, as illustrated in Fig. 10, by a root-finding function that is called by the modified version of ‘*de*’ before any ‘*de*’-internal (predictor or corrector) step. Additionally, the origin of a stick coordinate system $\overline{{\mathcal {K}}}_1$ (Fig. 11) is fixed in ${\mathcal {K}}_{0}$’s ${\mathsf {x}}$-${\mathsf {y}}$-plane at the position where contact of the origin of ${\mathcal {K}}_{1}$ has been detected by interpolation. The event detection, ordering, and handling part of the modified ‘*de*’ algorithm guarantees that, if an event earlier than those already found is newly detected, the integration is always restarted at the instant of the earliest event found so far.

**Table 2:** The model used in this study has in total eight foot–ground contact points defined (four on each foot), with the positions of the origins of the reference coordinate systems ${\mathcal {K}}_0^{\text {a-d,l/r}}$ given below

	$\rho _{\perp }^{01}[\;]$	$\rho _{\perp }^{11}\left[ \frac{\text {N}\cdot \text {s}}{\text {m}^2}\right]$	$\sigma [\;]$	$\mu ~[\;]$		$\mu _{\phi }[\;]$
100000.0	100.0	100000.0	0.0	0.7	0.001	0.0

		$\rho _{\parallel }\left[ \frac{\text {N}\cdot \text {s}}{\text {m}}\right]$	$\mu _{c}[\;]$		$\rho _{\phi }\left[ \frac{\text {N}\cdot \text {m}}{\text {rad}}\right]$	$\mu _{c \phi }\left[ \text {m}\right]$
99.0	50000.0	50.0	0.8	200.0	20.0	99.0

${\mathcal {K}}_0$	$x~[\text {m}]$	$y~[\text {m}]$	$z~[\text {m}]$			
${\mathcal {K}}_0^{\text {a,l/r}}$	$-0.1085$	$-0.0391$	$-0.0361$			
${\mathcal {K}}_0^{\text {b,l/r}}$	$-0.1085$	0.0391	$-0.0361$			
${\mathcal {K}}_0^{\text {c,l/r}}$	0.0678	$-0.0391$	$-0.0361$			
${\mathcal {K}}_0^{\text {d,l/r}}$	0.0678	0.0391	$-0.0361$			

All contacts use the same set of parameters (listed below) and produce forces w.r.t the ‘from’ body’s coordinate frame ${\mathcal {K}}_1^\text {w}$ that is fixed to the world at the origin $[0,\,0,\,0]$

Elastic stick forces and torques are then calculated depending on the linear and angular displacements of ${\mathcal {K}}_{1}$ relative to $\overline{{\mathcal {K}}}_1$, and friction contributions depending on their relative linear and angular velocities. The ‘slip’ and ‘stick’ force laws for modelling the contact interaction force $\mathbf {f}$ and torque $\varvec{\tau }$ as acting on the ‘from’-body are explained in the following. The (tangential force and torsional torque) limit values of maximum static friction, which determine the stick–slip transition, are parameterised by the coefficients of static friction, $\mu _{c}$ and $\mu _{c \phi }$:

63 $$\begin{aligned} f_{c} = \mu _{c} \cdot |f_{\perp }| \; \text {and} \; \tau _{c} = \mu _{c \phi } \cdot |f_{\perp }| \quad . \end{aligned}$$

In case of contact, the normal (to ${\mathcal {K}}_{0}$’s ${\mathsf {x}}$-${\mathsf {y}}$-plane) component $f_{\perp }$ (i.e. the projection onto ${\mathcal {K}}_{0}$’s ${\mathsf {z}}$-axis) of the contact force $\mathbf {f}$ is always calculated from the normal component $\text {r}_{\perp }$ of the distance vector of the origins of the contact coordinate systems ${\mathcal {K}}_{0}$ and ${\mathcal {K}}_{1}$, and the time derivative $\text {v}_{\perp }$ of $\text {r}_{\perp }$, that is, the projection of the relative velocity onto ${\mathcal {K}}_{0}$’s ${\mathsf {z}}$-axis:

64 $$\begin{aligned} f_{\perp } = \kappa _{\perp } \cdot \text {r}_{\perp } + \rho _{\perp }^{01} \cdot \text {v}_{\perp } - \rho _{\perp }^{11} \cdot \text {r}_{\perp } \cdot \text {v}_{\perp } \quad . \end{aligned}$$

Here, $\kappa _{\perp }$ is the normal stiffness of the contact interaction, and $\rho _{\perp }^{01}$ and $\rho _{\perp }^{11}$ are the damping coefficient and the nonlinear damping strength, respectively, of normal deformation rates. If, according to the decision scheme based on Eq. (63) as shown in Fig. 10, the contact has switched to the ‘slip’ state, the corresponding tangential (projection onto ${\mathcal {K}}_{0}$’s ${\mathsf {x}}$-${\mathsf {y}}$-plane of contact) force vector ${\varvec{f}}_{\parallel }$ of dynamic friction and the normal torque component $\tau _{\perp }$ modelling torsional friction are determined by (i) the normal force component $f_{\perp }$, (ii) the projection vector $\mathbf {v}_{\parallel }$ onto the contact plane (i.e. components tangential to it) of the difference $\mathbf {v} = \mathbf {v}_{{\mathcal {K}}_{1}} - \mathbf {v}_{{\mathcal {K}}_{0}}$ between the linear velocity vectors of the origins of ${\mathcal {K}}_{1}$ ($\mathbf {v}_{{\mathcal {K}}_{1}} = \dot{\mathbf {r}}_{{\mathcal {K}}_{1}}$) and ${\mathcal {K}}_{0}$ ($\mathbf {v}_{{\mathcal {K}}_{0}} = \dot{\mathbf {r}}_{{\mathcal {K}}_{0}}$), and (iii) the projection $\omega _{\perp }$ onto ${\mathcal {K}}_{0}$’s ${\mathsf {z}}$-axis of the angular velocity $\varvec{\omega }$ of ${\mathcal {K}}_{1}$ relative to ${\mathcal {K}}_{0}$, respectively. In that, the (tangential) dynamic friction force can be modelled as consisting of both a contribution linearly proportional to velocity and a *Coulomb* component, whereas the torsional torque is solely made of a *Coulomb* component:

65 $$\begin{aligned} {\varvec{f}}_{\parallel } = \sigma \cdot \mathbf {v}_{\parallel } + \mu \cdot |f_{\perp }| \cdot \frac{\mathbf {v}_{\parallel }}{|\mathbf {v}_{\parallel }|} \; \text {and} \; \tau _{\perp } = \mu _{\phi } \cdot |f_{\perp }| \cdot \frac{\omega _{\perp }}{|\omega _{\perp }|}\nonumber \\ \quad . \end{aligned}$$

Here, $\mu$ and $\mu _{\phi }$ are the coefficients of dynamic friction, and $\sigma$ is the frictional damping coefficient. If, in contrast, the contact has switched to the ‘stick’ state, the tangential force ${\varvec{f}}_{\parallel }$ always acts to drive ${\mathcal {K}}_{1}$—with the ‘to’-body attached—back to the stick contact point (origin of $\overline{{\mathcal {K}}}_1$)—with the ‘from’-body attached—which is fixed to ${\mathcal {K}}_{0}$’s ${\mathsf {x}}$-${\mathsf {y}}$-plane of contact in the ‘stick’ state, at the position where ${\mathcal {K}}_{1}$’s origin projected onto the contact plane at the instant when the ‘stick’ event occurred. Then, ${\varvec{f}}_{\parallel }$ is determined by the displacement $\mathbf {r}_{\parallel } - \overline{\mathbf {r}}_{\parallel }$ of the projection vector $\mathbf {r}_{\parallel }$ of the contact point (${\mathcal {K}}_{1}$’s origin) onto the contact plane from the respective projection vector $\overline{\mathbf {r}}_{\parallel }$ of the stick coordinate system $\overline{{\mathcal {K}}}_1$ (Fig. 11), which is fixed in the contact plane, and the vector of the tangential linear velocity vector $\overline{\mathbf {v}}_{\parallel } = \dot{\mathbf {r}}_{\parallel } - \dot{\overline{\mathbf {r}}}_{\parallel }$ (note: *different* from $\mathbf {v}_{\parallel }$ in (ii) above) of the contact point within the contact plane:

66 $$\begin{aligned} {\varvec{f}}_{\parallel } = \kappa _{\parallel } \cdot ( \mathbf {r}_{\parallel } - \overline{\mathbf {r}}_{\parallel } ) + \rho _{\parallel } \cdot \overline{\mathbf {v}}_{\parallel } \quad . \end{aligned}$$

Here, $\kappa _{\parallel }$ is the tangential stiffness of the contact interaction, and $\rho _{\parallel }$ is the damping coefficient of the tangential deformation rate. Moreover, if ${\mathcal {K}}_{1}$ rotates in the ‘stick’ state relative to $\overline{{\mathcal {K}}}_1$, a restoring torsional torque around the axis normal to the contact plane ($\overline{{\mathcal {K}}}_1$’s $\overline{{\mathsf {z}}}$-axis and ${\mathcal {K}}_{0}$’s ${\mathsf {z}}$-axis aligning) acts between ${\mathcal {K}}_{1}$ and ${\mathcal {K}}_{0}$:

67 $$\begin{aligned} \tau _{\perp } = \kappa _{\phi } \cdot \varDelta \xi + \rho _{\phi } \cdot \omega _{\perp } \quad . \end{aligned}$$

Here, $\omega _{\perp }$ is the component perpendicular to the contact plane of the angular velocity $\varvec{\omega }$ of ${\mathcal {K}}_{1}$ relative to ${\mathcal {K}}_{0}$, $\rho _{\phi }$ the corresponding angular damping coefficient, and $\kappa _{\phi }$ the angular stiffness of torsion. The symbol $\varDelta \xi = \xi - {\overline{\xi }}$ is the torsional angular excursion of ${\mathcal {K}}_{1}$ (relative to ${\mathcal {K}}_{0}$) from its (torsion) angle ${\overline{\xi }} = {\overline{\phi }} + {\overline{\psi }}$ determined at the instant of the ‘stick’ event, that is, ${\mathcal {K}}_{1}$’s instantaneous torsional excursion relative to ${\mathcal {K}}_{1}$’s orientation $\overline{{\mathcal {K}}}_1$ as fixed at the ‘stick’ event. The calculation of the instantaneous torsion angle $\xi = \phi + \psi$ is illustrated in Fig. 11. The used parameters for this contact model and the position of the contact points on the model’s foot bodies are listed in Table 2.

## Details on the calculations of the angle–length Jacobian ${J}^{{\varvec{\lambda }}{\varvec{\theta }}}$

The angle–length Jacobian matrix ${J}^{{\varvec{\lambda }}{\varvec{\theta }}}=\frac{\partial {\varvec{l}}^{\mathrm{CE}}}{\partial {\varvec{\theta }}}\in {\mathbb {R}}^{{n_\text {MTU}}\times {n_\theta }}$ relates the change of the MTU’s CE length ${\varvec{l}}^{\mathrm{CE}}\in {\mathbb {R}}^{{n_\text {MTU}}}$ to a change of the joint angles ${\varvec{\theta }}\in {\mathbb {R}}^{{n_\theta }}$. It is used to transform a control signal from the transformational layer to the structural layer. The definition of ${J}^{{\varvec{\lambda }}{\varvec{\theta }}}$ is similar to the definition of the matrix of moment arms (8), which is also the basis of the calculation of ${J}^{{\varvec{\lambda }}{\varvec{\theta }}}$ when combined with the MTU length equation (3):

68 $$\begin{aligned} {{R}}({\varvec{\theta }})= & {} \frac{\partial {\varvec{l}}^{\mathrm{MTU}}}{\partial {\varvec{\theta }}} = \frac{\partial \left( {\varvec{l}}^{\mathrm{CE}}+{\varvec{l}}^{\mathrm{SEE}}\right) }{\partial {\varvec{\theta }}} =\frac{\partial {\varvec{l}}^{\mathrm{CE}}}{\partial {\varvec{\theta }}}+ \frac{\partial {\varvec{l}}^{\mathrm{SEE}}}{\partial {\varvec{\theta }}} \end{aligned}$$

69 $$\begin{aligned}= & {} \frac{\partial {\varvec{l}}^{\mathrm{CE}}}{\partial {\varvec{\theta }}}+ \frac{\partial {\varvec{l}}^{\mathrm{SEE}}}{\partial {\varvec{l}}^{\mathrm{CE}}}\cdot \frac{\partial {\varvec{l}}^{\mathrm{CE}}}{\partial {\varvec{\theta }}} \end{aligned}$$

70 $$\begin{aligned}= & {} \left( I_{n_\text {MTU}}+\frac{\partial {\varvec{l}}^{\mathrm{SEE}}}{\partial {\varvec{l}}^{\mathrm{CE}}}\right) \cdot \frac{\partial {\varvec{l}}^{\mathrm{CE}}}{\partial {\varvec{\theta }}}, \end{aligned}$$

with $I_{n_\text {MTU}}$ being the identity matrix of dimension ${n_\text {MTU}}$. By substituting ${J}^{{\varvec{\lambda }}{\varvec{\theta }}}:=\frac{\partial {\varvec{l}}^{\mathrm{CE}}}{\partial {\varvec{\theta }}}$ and rearranging (70) to have ${J}^{{\varvec{\lambda }}{\varvec{\theta }}}$ on the left-hand side of the equation, the angle–length Jacobian matrix is expressed as

71 $$\begin{aligned} {J}^{{\varvec{\lambda }}{\varvec{\theta }}}=\left( {I}_{{n_\text {MTU}}}+\frac{\partial {\varvec{l}}^{\mathrm{SEE}}}{\partial {\varvec{l}}^{\mathrm{CE}}}\right) ^{-1}\cdot {{R}}({\varvec{\theta }}). \end{aligned}$$

The partial derivative $\frac{\partial {\varvec{l}}^{\mathrm{SEE}}}{\partial {\varvec{l}}^{\mathrm{CE}}}=\mathrm {diag\left( {\partial l^{\mathrm{SEE}}_i}/{\partial l^{\mathrm{CE}}_i}\right) }$ with $i=1\,\ldots ,\,{n_\text {MTU}}$, describes the change of the length of the SEE w.r.t. the change of the length of the CE. This term can be obtained by calculating the stiffness relation of the elements of the MTU under quasi-static assumptions.

### Calculation of ${\partial {\varvec{l}}^{\mathrm{SEE}}}/{\partial {\varvec{l}}^{\mathrm{CE}}}$

To calculate this term, the partial derivative of the MTU forces ${\varvec{f}}^\text {MTU}$ w.r.t. the CE lengths ${\varvec{l}}^{\mathrm{CE}}$ is evaluated in a matrix equation, while considering the force equilibrium (4):

72 $$\begin{aligned} \frac{\partial {\varvec{f}}^\text {MTU}}{\partial {\varvec{l}}^{\mathrm{CE}}}= \frac{\partial {\varvec{f}}^{\mathrm{CE}}}{\partial {\varvec{l}}^{\mathrm{CE}}} + \frac{\partial {\varvec{f}}^{\mathrm{PEE}}}{\partial {\varvec{l}}^{\mathrm{CE}}} = \frac{\partial {\varvec{f}}^\text {SDE}}{\partial {\varvec{l}}^{\mathrm{CE}}} + \frac{\partial {\varvec{f}}^\text {SEE}}{\partial {\varvec{l}}^{\mathrm{CE}}} \end{aligned}$$

Here, the partial derivative of the SEE force vector ${\varvec{f}}^\text {SEE}$ w.r.t. the CE length vector ${\varvec{l}}^{\mathrm{CE}}$ can be expressed as $\frac{\partial {\varvec{f}}^\text {SEE}}{\partial {\varvec{l}}^{\mathrm{CE}}}=\frac{\partial {\varvec{f}}^\text {SEE}}{\partial {\varvec{l}}^{\mathrm{SEE}}}\cdot \frac{\partial {\varvec{l}}^{\mathrm{SEE}}}{\partial {\varvec{l}}^{\mathrm{CE}}}$ and is assumed to be nonzero, i.e. the tendon is not slack. With this equation (72) is rearranged to:

73 $$\begin{aligned} \frac{\partial {\varvec{l}}^{\mathrm{SEE}}}{\partial {\varvec{l}}^{\mathrm{CE}}}= \left( \frac{\partial {\varvec{f}}^{\mathrm{CE}}}{\partial {\varvec{l}}^{\mathrm{CE}}} + \frac{\partial {\varvec{f}}^{\mathrm{PEE}}}{\partial {\varvec{l}}^{\mathrm{CE}}} - \frac{\partial {\varvec{f}}^\text {SDE}}{\partial {\varvec{l}}^{\mathrm{CE}}}\right) \cdot \left( \frac{\partial {\varvec{f}}^\text {SEE}}{\partial {\varvec{l}}^{\mathrm{SEE}}}\right) ^{-1}. \end{aligned}$$

According to equations (48, 51, 53), the functions ${\varvec{f}}^{\mathrm{CE}}$ and ${\varvec{f}}^{\mathrm{PEE}}$ depend on ${\varvec{l}}^{\mathrm{CE}}$, while ${\varvec{f}}^\text {SEE}$ depends on ${\varvec{l}}^{\mathrm{SEE}}$. By assuming quasi-static conditions ($\dot{{\varvec{l}}}^\text {MTU}\approxeq {\dot{l}}^{\mathrm{CE}}\approxeq 0$), the SDE force vanishes (${\varvec{f}}^\text {SDE}\approx 0$) and the CE force (48) of a *k*-th MTU simplifies to $f^{\mathrm{CE}}_k(l^{\mathrm{CE}}_k)=f^{\mathrm{CE}}_{\mathrm {max},k}\cdot a_k({\varvec{l}}^{\mathrm{CE}}_k)\cdot f_{\mathrm {isom},k}(l^{\mathrm{CE}},k)$.

Subsequently, equation (73) can be written to the matrix equation

74 $$\begin{aligned} \frac{\partial {\varvec{l}}^{\mathrm{SEE}}}{\partial {\varvec{l}}^{\mathrm{CE}}}= & {} \left( \frac{\partial {\varvec{f}}^{\mathrm{CE}}}{\partial {\varvec{l}}^{\mathrm{CE}}} + \frac{\partial {\varvec{f}}^{\mathrm{PEE}}}{\partial {\varvec{l}}^{\mathrm{CE}}} \right) \cdot \left( \frac{\partial {\varvec{f}}^\text {SEE}}{\partial {\varvec{l}}^{\mathrm{SEE}}}\right) ^{-1}\nonumber \\= & {} \left( F_\mathrm {max}\cdot \left( \frac{\partial {\varvec{a}}}{\partial {\varvec{l}}^{\mathrm{CE}}}\cdot {\varvec{f}}^{\mathrm{CE}}_\mathrm {isom} + {\varvec{a}}\cdot \frac{\partial {\varvec{f}}^{\mathrm{CE}}_\mathrm {isom}}{\partial {\varvec{l}}^{\mathrm{CE}}}\right) +\frac{\partial {\varvec{f}}^{\mathrm{PEE}}}{\partial {\varvec{l}}^{\mathrm{CE}}}\right) \nonumber \\&\cdot \left( {\frac{\partial {\varvec{f}}^\text {SEE}}{\partial {\varvec{l}}^{\mathrm{SEE}}}}\right) ^{-1}. \end{aligned}$$

and thus, the partial derivatives of ${\varvec{a}}({\varvec{l}}^{\mathrm{CE}})$, ${\varvec{f}}^\text {isom}({\varvec{l}}^{\mathrm{CE}})$, ${\varvec{f}}^{\mathrm{PEE}}({\varvec{l}}^{\mathrm{CE}})$ and ${\varvec{f}}^\text {SEE}({\varvec{l}}^{\mathrm{SEE}})$ remain to be calculated. Given the PEE force equation (51), its derivative w.r.t ${\varvec{l}}^{\mathrm{CE}}$ is given in equation (52) in Appendix A.1.2. With the serial elastic force (53), its derivative w.r.t. to ${\varvec{l}}^{\mathrm{SEE}}$ can be calculated and the result is stated in (54) in Appendix A.1.3. The derivative of the isometric force (49) is given in (50) in Appendix A.1.1. The nonlinear activation ${\varvec{a}}({\varvec{l}}^{\mathrm{CE}}, {\varvec{\gamma }})$ is given together with its derivative w.r.t ${\varvec{l}}^{\mathrm{CE}}$ in Appendix A.2 in the equations (56-59). With those equations all terms of equation (74) are sufficiently resolved and $\frac{\partial {\varvec{l}}^{\mathrm{SEE}}}{\partial {\varvec{l}}^{\mathrm{CE}}}$ can be calculated during runtime at each time-step of the simulation, using the respective parameters. The above calculation (74) is used in this contribution for the calculation of the angle–length Jacobian (71).

To estimate the impact of the quasi-static assumption from above (i.e. $\dot{{\varvec{l}}}^\text {MTU}\approxeq \dot{{\varvec{l}}}^\text {CE}\approxeq 0$), a brief estimation without it follows. Calculating the partial derivative $\frac{\partial {\varvec{f}}^\text {SDE}}{\partial {\varvec{l}}^{\mathrm{CE}}}$ in (73) from (55) with $R^\mathrm {SDE}\approx 0$ (Bayer et al. 2017), (73) can be rewritten in the scalar case for the *k*-th MTU as

75 $$\begin{aligned} \frac{\partial l^{\mathrm{SEE}}_k}{\partial l^{\mathrm{CE}}_k}= \left( 1-\left( \frac{D^{\mathrm{SDE}}_{{\mathrm{max}},k}\cdot ({\dot{l}}^{\mathrm{MTU}}_k-{\dot{l}}^{\mathrm{CE}}_k)}{f^{\mathrm{CE}}_{{\mathrm{max}},k}}\right) \right) \nonumber \\ \cdot \left( \frac{\partial f^{\mathrm{CE}}_k}{\partial l^{\mathrm{CE}}_k} + \frac{\partial f^{\mathrm{PEE}}_k}{\partial l^{\mathrm{CE}}_k} \right) \big /\frac{\partial f^{\mathrm{SEE}}_k}{\partial l^{\mathrm{SEE}}_k}. \end{aligned}$$

Additionally estimating the SEE contraction velocity to be as high as the maximum concentric contraction velocity of the CE for the *k*-th MTU, i.e. ${\dot{l}}^\mathrm {SEE}_k=({\dot{l}}^{\mathrm{MTU}}_k-{\dot{l}}^{\mathrm{CE}}_k)=v_{\mathrm {max},k} =\frac{B_{\mathrm {rel},k}}{A_{\mathrm {rel},k}}\cdot l^{\mathrm{CE}}_{\text {opt},k}$, (75) contracts to (for substituting $D^\mathrm {SDE}_{\mathrm {max},k}$, see Appendix A.1.4)

76 $$\begin{aligned} \frac{\partial l^{\mathrm{SEE}}_k}{\partial l^{\mathrm{CE}}_k}= (1-D^{{\mathrm{SDE}}}_k)\cdot \left( \frac{\partial f^{\mathrm{CE}}_k}{\partial l^{\mathrm{CE}}_k} + \frac{\partial f^{\mathrm{PEE}}_k}{\partial l^{\mathrm{CE}}_k} \right) \big /\frac{\partial f^{\mathrm{SEE}}_k}{\partial l^{\mathrm{SEE}}_k}. \end{aligned}$$

As the relative damping coefficient is about $D^\mathrm {SDE}_k=0.3$ for each of the $k=1\ldots {n_\text {MTU}}$ MTUs (Bayer et al. 2017), it is seen that (74) is a reasonable approximation, at least in all non-explosive movements.

### First-order Taylor approximation

The transformation of the discrete error signal ${\varvec{\theta }}^\text {err}$ with the Jacobian ${J}^{{\varvec{\lambda }}{\varvec{\theta }}}:=\frac{\partial {\varvec{l}}^{\mathrm{CE}}}{{\varvec{\theta }}}$, which relates infinitesimal changes, is achieved by integrating $\partial {\varvec{l}}^{\mathrm{CE}}={J}^{{\varvec{\lambda }}{\varvec{\theta }}}({\varvec{\theta }})\cdot \partial {\varvec{\theta }}$ with the limits of the respective error signals (i.e. ${\varvec{l}}^{\mathrm{CE}}_{\delta _\theta }(t)$ and ${\varvec{\lambda }}^\theta (t)$, and ${\varvec{\theta }}(t)$ and ${\varvec{\theta }}^\text {des}(t)$):

77 $$\begin{aligned} \int _{{\varvec{l}}^{\mathrm{CE}}_{\delta _\theta }}^{{\varvec{\lambda }}^\theta }\mathrm {d}{\varvec{l}}^{\mathrm{CE}}= & {} \int _{{\varvec{\theta }}_{\delta _\theta }}^{{\varvec{\theta }}^\text {des}} {J}^{{\varvec{\lambda }}{\varvec{\theta }}}({\varvec{\theta }})\cdot \mathrm {d}{\varvec{\theta }}, \qquad \text {with}\; \lambda ^\theta := \left. {\varvec{l}}^{\mathrm{CE}}\right| _{{\varvec{\theta }}^\text {des}}\nonumber \\ \end{aligned}$$

78 $$\begin{aligned} {\varvec{l}}^{\mathrm{CE}}_{\delta _\theta }-{\varvec{\lambda }}^\theta= & {} {\bar{J}}^{\lambda \theta }({\varvec{\theta }}_{\delta _\theta }) -{\bar{J}}^{\lambda \theta }({\varvec{\theta }}^\text {des}). \end{aligned}$$

Here the antiderivative ${\bar{J}}^{\lambda \theta }$ is an unknown, joint angle-dependent function ${\bar{J}}^{\lambda \theta }({\varvec{\theta }})$ that can be approximated by a first-order Taylor approximation at the set-point of the actual measured joint angle configuration ${\varvec{\theta }}^*={\varvec{\theta }}_{\delta _\theta }(t)$:

79 $$\begin{aligned} \left. {\bar{J}}^{\lambda \theta }({\varvec{\theta }})\right| _{{\varvec{\theta }}^*}= & {} {\bar{J}}^{\lambda \theta } ({\varvec{\theta }}^*)+\left. {J}^{{\varvec{\lambda }}{\varvec{\theta }}}\right| _{{\varvec{\theta }}*}\cdot ({\varvec{\theta }}-{\varvec{\theta }}^*) +{\mathcal {O}}({\varvec{\theta }}^2), \;\text {i.e.}\nonumber \\ \left. {\bar{J}}^{\lambda \theta }({\varvec{\theta }}^\text {des})\right| _{{\varvec{\theta }}^*={\varvec{\theta }}_{\delta _\theta }}\approx & {} {\bar{J}}^{\lambda \theta }({\varvec{\theta }}_{\delta _\theta })+{J}^{{\varvec{\lambda }}{\varvec{\theta }}}\cdot ({\varvec{\theta }}^\text {des}-{\varvec{\theta }}_{\delta _\theta }). \end{aligned}$$

The use of (79) in (78) cancels out all occurrences of the unknown antiderivative ${\bar{J}}^{\lambda \theta }$ and the following remains:

80 $$\begin{aligned} {\varvec{l}}^{\mathrm{CE}}_{\delta _\theta }-{\varvec{\lambda }}^\theta \approx {J}^{{\varvec{\lambda }}{\varvec{\theta }}}\cdot ({\varvec{\theta }}_{\delta _\theta }-{\varvec{\theta }}^\text {des}) \;\Leftrightarrow \;{\varvec{l}}^\text {err}_{{\varvec{\delta _\theta }}}\approx {J}^{{\varvec{\lambda }}{\varvec{\theta }}}{\varvec{\theta }}^\text {err}_{\delta _\theta }. \end{aligned}$$

The Jacobian matrix ${J}^{{\varvec{\lambda }}{\varvec{\theta }}}$ can thus be used to approximately transform the discrete control error ${\varvec{\theta }}^\text {err}$ from the transformational layer to a control error of CE lengths ${\varvec{l}}^\text {err}$ in the structural layer. Due to the linear approximation (79), the transformation is less accurate for large control errors. During a successful movement execution (${\varvec{\theta }}\rightarrow {\varvec{\theta }}^\text {des}$), this approximation becomes more accurate and, thus, the controller performances are more precise, the closer the system approaches the desired state.

## Details on the calculations of the length–stimulation Jacobian ${J}^{{\varvec{u}}{\varvec{\lambda }}}$

For the calculation of the length–stimulation Jacobian matrix ${J}^{{\varvec{u}}{\varvec{\lambda }}}:=\frac{\partial {\varvec{u}}}{\partial {\varvec{l}}^{\mathrm{CE}}}$ the activation dynamics (56) in steady state with ${\dot{\gamma }}_k=0$ ($\rightarrow \,\gamma _k(t)=u_k(t)$) are utilised. This yields a steady-state activation $a^\text {ss}_k(u_k(t),l^{\mathrm{CE}}_k(t))$, dependent on the current stimulation $u_k(t)$ and CE length $l^{\mathrm{CE}}_k(t)$ of the *k*-th MTU. In steady state, the activation $a^\text {ss}_k$ does not change and its total differential vanishes.

$$\begin{aligned} 0=\mathrm {d}a^\text {ss}_k=\frac{\partial a^\text {ss}_k}{\partial u_k}\mathrm {d}u_k+\frac{\partial a^\text {ss}_k}{\partial l^{\mathrm{CE}}_k}\mathrm {d}l^{\mathrm{CE}}_k, \end{aligned}$$

Using the partial derivatives (58-60) of $a^\text {ss}_k$ then leads to

81 $$\begin{aligned} 0=\frac{\mathrm {d} a^\text {ss}_k}{\mathrm {d} l^{\mathrm{CE}}_k}= & {} \frac{\partial a^\text {ss}_k}{\partial u_k}\cdot \frac{\mathrm {d} u_k}{\mathrm {d}l^{\mathrm{CE}}_k} +\frac{\partial a^\text {ss}_k}{\partial l^{\mathrm{CE}}_k}\nonumber \\ \Rightarrow \frac{\mathrm {d} u_k}{\mathrm {d} l^{\mathrm{CE}}_k}= & {} -\left. \frac{\partial a^\text {ss}_k}{\partial l^{\mathrm{CE}}_k} \big / \frac{\partial a^\text {ss}_k}{\partial u_k}\right. =-\frac{u_k}{l^{\mathrm{CE}}_k}\left( =j^{{\varvec{u}}{\varvec{\lambda }}}_k\right) . \end{aligned}$$

The length–stimulation Jacobian matrix ${J}^{{\varvec{u}}{\varvec{\lambda }}}$ is finally obtained as a diagonal matrix ${J}^{{\varvec{u}}{\varvec{\lambda }}}=\text {diag}(j^{{\varvec{u}}{\varvec{\lambda }}}_k)$ for $k=1\,\ldots \, {n_\text {MTU}}$.

### First-order Taylor approximation

With the same reasoning as for the transformation of the joint angle error ${\varvec{\theta }}^\text {err}$ from the transformational layer to the CE length error ${\varvec{l}}^\text {err}$ in the structural layer as described in Appendix B.2, the CE length error ${\varvec{l}}^\text {err}$ can be transformed with ${J}^{{\varvec{u}}{\varvec{\lambda }}}$ to an error ${\varvec{u}}^\text {err}$ of MTU stimulations. Firstly the discrete integration with the limits of ${\varvec{u}}^\text {coc}_\text {ref}$ and ${\varvec{u}}^\text {opn}_\text {task}$ on the left-hand side ${\varvec{l}}^{\mathrm{CE}}_{\delta _\theta }$ and ${\varvec{\lambda }}^\theta$ on the right-hand side is performed:

82 $$\begin{aligned}&\partial {\varvec{u}}={J}^{{\varvec{u}}{\varvec{\lambda }}}\cdot \partial {\varvec{l}}^{\mathrm{CE}}\quad \Leftrightarrow \quad \int _{{\varvec{u}}^\text {coc}_\text {ref}}^{{\varvec{u}}^\text {opn}_\text {task}}\text {d}{\varvec{u}}=\int _{{\varvec{l}}^{\mathrm{CE}}_{\delta _\theta }}^{{\varvec{\lambda }}^\theta }{J}^{{\varvec{u}}{\varvec{\lambda }}}({\varvec{l}}^{\mathrm{CE}})\text {d}{\varvec{l}}^{\mathrm{CE}}\nonumber \\&{\varvec{u}}^\text {coc}_\text {ref}-{\varvec{u}}^\text {opn}_\text {task}={\bar{J}}^{u\lambda }({\varvec{l}}^{\mathrm{CE}}_{\delta _\theta }) -{\bar{J}}^{u\lambda }({\varvec{\lambda }}^\theta ). \end{aligned}$$

With an analogue first-order Taylor approximation as in (79), the unknown antiderivatives ${\bar{J}}^{u\lambda }({\varvec{l}}^{\mathrm{CE}}_{\delta _\theta })$ and ${\bar{J}}^{u\lambda }({\varvec{\lambda }}^\theta )$ in (82) can be linearly estimated to obtain:

83 $$\begin{aligned} {\varvec{u}}^\text {err}={J}^{{\varvec{u}}{\varvec{\lambda }}}\cdot {\varvec{l}}^\text {err}+{\mathcal {O}}({{\varvec{l}}^{\mathrm{CE}}}^2) \end{aligned}$$

With this, an error of CE lengths can be transformed to an error of the reference co-contraction (base stimulation level) ${\varvec{u}}^\text {coc}_\text {ref}$ and the desired task fulfilling stimulation ${\varvec{u}}^\text {opn}_{\text {task}}$. By rearranging, the task fulfilling stimulation ${\varvec{u}}^\text {opn}_\text {task}$ can be obtained, based on the transformed CE length error ${\varvec{l}}^\text {err}$ and the chosen reference stimulation level ${\varvec{u}}^\text {coc}_\text {ref}$ (see also 31).

## Details on the calculations of the torque–angle Jacobian ${J}^{{\varvec{\theta }}{\varvec{\tau }}}$

The torque–angle Jacobian matrix defined as ${J}^{{\varvec{\theta }}{\varvec{\tau }}}:=\frac{\partial {\varvec{\theta }}}{\partial {\varvec{\tau }}^\text {MTU}}$. is the inverse of the joint stiffness matrix $K_\theta$ (Stanev and Moustakas 2019), ${J}^{{\varvec{\theta }}{\varvec{\tau }}}=K_\theta ^{-1}$, with $K_\theta =\frac{\partial {\varvec{\tau }}^\text {MTU}}{\partial {\varvec{\theta }}}$. which can be calculated by inserting the definition of the joint torques, i.e. the generalised forces generated by the MTUs (9):

84 $$\begin{aligned} K_\theta =\frac{\partial (-{{R}}^T\cdot {\varvec{f}}^\text {MTU})}{\partial {\varvec{\theta }}}. \end{aligned}$$

Applying the product rule and inserting the definition (17) of the angle–length Jacobian yields

85 $$\begin{aligned} K_\theta = -\frac{\partial {{R}}^T}{\partial {\varvec{\theta }}}\bullet _2 {\varvec{f}}^\text {MTU}- {{R}}^T\frac{\partial {\varvec{f}}^\text {MTU}}{\partial {\varvec{l}}^{\mathrm{CE}}} {J}^{{\varvec{\lambda }}{\varvec{\theta }}}. \end{aligned}$$

where $\frac{\partial {{R}}^T}{\partial {\varvec{\theta }}}\in {\mathbb {R}}^{{n_\theta }\times {n_\theta }\times {n_\text {MTU}}}$ is a third-order tensor and $\bullet _2$ denotes a tensor product, leading to $(\frac{\partial {{R}}^T}{\partial {\varvec{\theta }}}\bullet _2 {\varvec{f}}^\text {MTU})\in {\mathbb {R}}^{{n_\theta }\times {n_\theta }}$ being a second-order tensor (Kolda and Bader 2009; Stanev and Moustakas 2019).

The calculation of $\partial {\varvec{f}}^\text {MTU}/\partial {\varvec{l}}^{\mathrm{CE}}$ in (85) follows similar calculations as already given in Appendix B. For the simulation study of this paper, the first part of the joint stiffness matrix $K_\theta$ is neglected due to the assumption of small changes in the joint angles during quiet upright standing and hence, the torque–angle Jacobian is used as

86 $$\begin{aligned} {J}^{{\varvec{\theta }}{\varvec{\tau }}}=\left( -{{R}}^T\frac{\partial {\varvec{f}}^\text {MTU}}{\partial {\varvec{l}}^{\mathrm{CE}}}{J}^{{\varvec{\lambda }}{\varvec{\theta }}}\right) ^{-1}. \end{aligned}$$

### First-order Taylor approximation

To transform the discrete error between the actual and desired torques ${\varvec{\tau }}^\text {err}$ from the conceptional layer to an error of joint angles ${\varvec{\theta }}^\text {err}$ in the transformational layer, analogously as in Appendix B.2 and C.1 , firstly an integration with the limits of ${\varvec{\tau }}_{\delta _\tau }$ and ${\varvec{\tau }}^\text {des}$, and ${\varvec{\theta }}_{\delta _\tau }$ and ${\varvec{\theta }}^\text {des}$ is performed:

87 $$\begin{aligned} \partial {\varvec{\theta }}= & {} {J}^{{\varvec{\theta }}{\varvec{\tau }}}\cdot \partial {\varvec{\tau }}\quad \Leftrightarrow \quad \int _{{\varvec{\theta }}_{\delta _\tau }}^{{\varvec{\theta }}^\text {des}}\text {d}{\varvec{\theta }}= \int _{{\varvec{\tau }}_{\delta _\tau }}^{{\varvec{\tau }}^\text {des}}{J}^{{\varvec{\theta }}{\varvec{\tau }}}({\varvec{\tau }})\text {d}{\varvec{\tau }}\nonumber \\ {\varvec{\theta }}_{\delta _\tau }-{\varvec{\theta }}^\text {des}= & {} {\bar{J}}^{\theta \tau }({\varvec{\tau }}_{\delta _\tau })-{\bar{J}}^{\theta \tau }({\varvec{\tau }}^\text {des}). \end{aligned}$$

The unknown antiderivative ${\bar{J}}^{\theta \tau }$ is eliminated by a first-order Taylor approximation in the same way as in (79), leading to the linear estimation ${\varvec{\theta }}^\text {err}={J}^{{\varvec{\theta }}{\varvec{\tau }}}{\varvec{\tau }}^\text {err}+{\mathcal {O}}({\varvec{\tau }}^2)$.

Thus, the torque error ${\varvec{\tau }}^\text {err}$ can approximately related to an joint angle error ${\varvec{\theta }}^\text {err}$. This linear approximation becomes more precise, the smaller the torque error is. For a large deviation in the actual and desired torques therefore only a rough estimation is present that becomes more detailed in the fine tuning of the controlled movement as ${\varvec{\tau }}\rightarrow {\varvec{\tau }}^\text {des}$.

## Simulation software and solver

To perform the here presented simulations, the C/C++ simulation environment *demoa* is used (Mörl et al. 2020; Rupp et al. 2015).

The simulation software *demoa* uses homogeneous matrix representations (Hartenberg and Denavit 1955) to algorithmically set-up the mechanical equations of motion (1), as described by Legnani et al. 1996b, a, using the C/C++ library *eigen* (Guennebaud et al. 2010). The integration method used within *demoa* for the mechanical equations of motion (1), the activation dynamics (7), and the MTU contraction dynamics (5) is based on the Shampine–Gordon algorithm ‘*de*’ (Shampine and Gordon 1975), which has been slightly modified (Henze 2002) to allow event handling (root finding). The simulations parameters are the same for the four simulations studies from Sect. 3.1. The maximum simulation step size is set to $0.001~\text {s}$ with relative and absolute error boundaries of $10^{-6}$.

The integration in the PID controllers (24, 16, 39) is performed using Euler’s method (first-order Runge–Kutta), and the differentiation in the ${\varvec{\tau }}$-PID controller (39) is performed by discrete differentiation based on the integration step time.

## Abbreviations

FE: Flexion–extension
AA: Abduction–adduction
Hp: Hip
Kn: Knee
An: Ankle
Lu: Lumbar joint
Ce: Cervical joint
Sh: Shoulder
Eb: Elbow
CNS: Central nervous system
COM: Centre of mass
DHM: Digital human model
MTU: Muscle–tendon unit
MTUs: Muscle–tendon units
CE: Contractile element
PEE: Parallel elastic element
SEE: Serial elastic element
SDE: Serial damping element
DoF: Degree of freedom
DoFs: Degrees of freedom
PID: Proportional–integral–derivative
PD: Proportional–derivative
Fx: Flexor
Ex: Extensor

## Footnotes

1 Despite some critique on the mathematical denotations (Arbib and Amari 1985) in the work of Pellionisz and Llinás (1985), their work is still a suitable tool in the computational analysis of movement organisation (Habas et al. 2020).

2 The integrative part, on the other hand, may show so-called wind-up behaviour, wherefore a simple anti-wind-up technique may be included if required, e.g. see Tarbouriech and Turner (2009)

## Code availability

The code of our mechanics engine *demoa* is available on request.

## References

[CR1] Alexandrov AV, Frolov AA, Massion J (2001). Biomechanical analysis of movement strategies in human forward trunk bending I. Modeling. Biol Cybern.

[CR2] Arbib MA, Amari S-I (1985). Sensori-motor transformations in the brain (with a critique of the tensor theory of cerebellum). J Theor Biol.

[CR3] Bayer A, Schmitt S, Günther M, Haeufle DF (2017). The influence of biophysical muscle properties on simulating fast human arm movements. Comput Methods Biomech Biomed Eng.

[CR4] Bernstein NA (1967). The co-ordination and regulation of movements.

[CR5] Bizzi E, Hogan N, Mussa-Ivaldo F, Giszter S (1992). Does the nervous system use equilibrium-point control to guide single and multiple joint movements?. Behav Brain Sci.

[CR6] Blakemore S-J, Frith CD, Wolpert DM (1999). Spatio-temporal prediction modulates the perception of self-produced stimuli. J Cogn Neurosci.

[CR7] Brändle S, Schmitt S, Müller MA (2020). A systems-theoretic analysis of low-level human motor control: application to a single-joint arm model. J Math Biol.

[CR8] de Luca CJ, Contessa P (2012). Hierarchical control of motor units in voluntary contractions. J Neurophysiol.

[CR9] De Serres S, Milner T (1991). Wrist muscle activation patterns and stiffness associated with stable and unstable mechanical loads. Exp Brain Res.

[CR10] DeWolf T, Eliasmith C (2011). The neural optimal control hierarchy for motor control. J Neural Eng.

[CR11] Doya K (2000). Complementary roles of basal ganglia and cerebellum in learning and motor control. Curr Opin Neurobiol.

[CR12] Edwards WT (2007). Effect of joint stiffness on standing stability. Gait Posture.

[CR13] Feldman A (1974). Control of the length of a muscle. Biophysics.

[CR14] Ford L, Huxley A, Simmons R (1977). Tension responses to sudden length change in stimulated frog muscle fibres near slack length. J Physiol.

[CR15] Full R, Koditschek D (1999). Templates and anchors: neuromechanical hypotheses of legged locomotion on land. J Exp Biol.

[CR16] Ganesh G, Nakamura K, Saetia S, Tobar AM, Yoshida E, Ando H, Yoshimura N, Koike Y (2018). Utilizing sensory prediction errors for movement intention decoding: a new methodology. Sci Adv.

[CR17] Gao J-H, Parsons LM, Bower JM, Xiong J, Li J, Fox PT (1996). Cerebellum implicated in sensory acquisition and discrimination rather than motor control. Science.

[CR18] Gentili R, Han CE, Schweighofer N, Papaxanthis C (2010). Motor learning without doing: trial-by-trial improvement in motor performance during mental training. J Neurophysiol.

[CR19] Ghazi-Zahedi K (2019). Morphological Intelligence.

[CR20] Gribble P, Mullin L, Cothros N, Mattar A (2003). Role of cocontraction in arm movement accuracy. J Neurophysiol.

[CR21] Gribble P, Ostry D, Sanguineti V, Laboissière R (1998). Are complex control signals required for human arm movement?. J Neurophysiol.

[CR22] Guennebaud G, Jacob B et al (2010) Eigen v3. http://eigen.tuxfamily.org

[CR23] Günther M (1997) Computersimulationen zur Synthetisierung des muskulär erzeugten menschlichen Gehens unter Verwendung eines biomechanischen Mehrkörpermodells. Ph.D. thesis, Eberhard-Karls-Universität, Tübingen, Germany

[CR24] Günther M, Haeufle DFB, Schmitt S (2018). The basic mechanical structure of the skeletal muscle machinery: one model for linking microscopic and macroscopic scales. J Theor Biol.

[CR25] Günther M, Müller O, Blickhan R (2011). Watching quiet human stance to shake off its straitjacket. Arch Appl Mech.

[CR26] Günther M, Müller O, Blickhan R (2012). What does head movement tell about the minimum number of mechanical degrees of freedom in quiet human stance?. Arch Appl Mech.

[CR27] Günther M, Ruder H (2003). Synthesis of two-dimensional human walking: a test of the $$\lambda $$-model. Biol Cybern.

[CR28] Günther M, Wagner H (2016). Dynamics of quiet human stance: computer simulations of a triple inverted pendulum model. Comput Methods Biomech Biomed Eng.

[CR29] Habas C, Bertholz A, Flash T, Bennequin D (2020). Does the cerebellum implement or select geometries? A speculative note. The Cerebellum.

[CR30] Haeufle DFB, Günther M, Bayer A, Schmitt S (2014). Hill-type muscle model with serial damping and eccentric force–velocity relation. J Biomech.

[CR31] Haeufle DFB, Günther M, Wunner G, Schmitt S (2014). Quantifying control effort of biological and technical movements: an information-entropy-based approach. Phys Rev E.

[CR32] Hammer M, Günther M, Haeufle D, Schmitt S (2019). Tailoring anatomical muscle paths: a sheath-like solution for muscle routing in musculo-skeletal computer models. Math Biosci.

[CR33] Hartenberg RS, Denavit J (1955). A kinematic notation for lower pair mechanisms based on matrices. J Appl Mech.

[CR34] Hatze H (1977). A myocybernetic control model of skeletal muscle. Biol Cybern.

[CR35] Henze A (2002) Dreidimensionale biomechanische Modellierung und die Entwicklung eines Reglers zur Simulation zweibeinigen Gehens. Ph.D. thesis, Eberhard-Karls-Universität, Tübingen, Germany

[CR36] Herzfeld DJ, Shadmehr R (2014). Cerebellum estimates the sensory state of the body. Trends Cognit Sci.

[CR37] Hogan N, Winters JM, Woo SL-Y (1990). Chapter 9: Mechanical impedance of single- and multi-articular systems. Multiple muscle systems.

[CR38] Holmes P, Full R, Koditschek D, Guckenheimer J (2006). The dynamics of legged locomotion: models, analyses, and challenges. SIAM Rev.

[CR39] Hsu W-L, Scholz JP (2012). Motor abundance supports multitasking while standing. Hum Mov Sci.

[CR40] Hulliger M, Dürmüller N, Prochazka A, Trend P (1989). Flexible fusimotor control of muscle spindle feedback during a variety of natural movements. Prog Brain Res.

[CR41] Kiehn O (2016). Decoding the organization of spinal circuits that control locomotion. Nat Rev Neurosci.

[CR42] Kistemaker DA, van Soest AJ, Bobbert MF (2006). Is equilibrium point control feasible for fast goal-directed single-joint movements?. J Neurophysiol.

[CR43] Kistemaker DA, van Soest AJ, Bobbert MF (2007). A model of open-loop control of equilibrium position and stiffness of the human elbow joint. Biol Cybern.

[CR44] Kolda TG, Bader BW (2009). Tensor decompositions and applications. SIAM Rev.

[CR45] Latash ML (2012). The bliss (not the problem) of motor abundance (not redundancy). Exp Brain Res.

[CR46] Latash ML, Scholz JP, Schöner G (2002). Motor control strategies revealed in the structure of motor variability. Exerc Sport Sci Rev.

[CR47] Legnani G, Casalo F, Righettini P, Zappa B (1996). A homogeneous matrix approach to 3d kinematics and dynamics - II. Applications to chains of rigid bodies and serial manipulators. Mech Mach Theory.

[CR48] Legnani G, Casolo F, Righettini P, Zappa B (1996). A homogeneous matrix approach to 3d kinematics and dynamics—I. Theory. Mech Mach Theory.

[CR49] Martin JH (2005). The corticospinal system: from development to motor control. Neuroscientist.

[CR50] Matthews P (1959). A study of certain factors influencing the stretch reflex of the decerebrate cat. J Physiol.

[CR51] McIntyre J, Bizzi E (1993). Servo hypotheses for the biological control of movement. J Mot Behav.

[CR52] Merel J, Botvinick M, Wayne G (2019). Hierarchical motor control in mammals and machines. Nat Commun.

[CR53] Milner T (2002). Adaptation to destabilizing dynamics by means of muscle cocontraction. Exp Brain Res.

[CR54] Milner T, Cloutier C, Leger A, Franklin D (1995). Inability to activate muscles maximally during cocontraction and the effect on joint stiffness. Exp Brain Res.

[CR55] Mörl F, Gönther M, Riede JM, Hammer M, Schmitt S (2020) Loads distributed in vivo among vertebrae, muscles, spinal ligaments, and intervertebral discs in a passively flexed lumbar spine. Biomech Model Mechanobiol 19(6):2015–2047 10.1007/s10237-020-01322-7 32314072

[CR56] Naito E, Morita T, Amemiya K (2016). Body representations in the human brain revealed by kinesthetic illusions and their essential contributions to motor control and corporeal awareness. Neurosci Res.

[CR57] Park JH (2001). Impedance control for biped robot locomotion. IEEE Trans Robot Autom.

[CR58] Pellionisz A, Llinás RR (1985). Tensor network theory of the metaorganization of functional geometries in the central nervous system. Neuroscience.

[CR59] Piazzesi G, Lombardi V (1995). A cross-bridge model that is able to explain mechanical and energetic properties of shortening muscle. Biophys J.

[CR60] Pinter IJ, van Soest AJ, Bobbert MF, Smeets JBJ (2012). Conclusions on motor control depend on the type of model used to represent the periphery. Biol Cybern.

[CR61] Pratt J, Dilworth P, Pratt G (1997) Virtual model control of a bipedal walking robot. In: Proceedings of the international conference on robotics and automation, vol 1. IEEE, pp 193–198

[CR62] Pratt J, Pratt G (1998) Intuitive control of a planar bipedal walking robot. In: Proceedings of the international conference on robotics and automation, vol 3. IEEE, pp 2014–2021

[CR63] Prescott TJ, Redgrave P, Gurney K (1999). Layered control architectures in robots and vertebrates. Adapt Behav.

[CR64] Rockenfeller R, Günther M (2017). Hill equation and Hatze’s muscle activation dynamics complement each other: enhanced pharmacological and physiological interpretability of modelled activity-pCa curves. J Theor Biol.

[CR65] Rockenfeller R, Günther M (2018). Inter-filament spacing mediates calcium binding to troponin: a simple geometric-mechanistic model explains the shift of force-length maxima with muscle activation. J Theor Biol.

[CR66] Rockenfeller R, Günther M, Schmitt S, Götz T (2015). Comparative sensitivity analysis of muscle activation dynamics. Comput Math Methods Med.

[CR67] Rozendaal LA, van Soest AJ (2005) Joint stiffness requirements in a multi-segment stance model. In: Proceedings of the XXth congress of the ISB, Cleveland, Ohio, p 622

[CR68] Rupp T, Ehlers W, Karajan N, Günther M, Schmitt S (2015). A forward dynamics simulation of human lumbar spine flexion predicting the load sharing of intervertebral discs, ligaments, and muscles. Biomech Model Mechanobiol.

[CR69] Schmitt S, Günther M, Haeufle DFB (2019). The dynamics of the skeletal muscle: a systems biophysics perspective on muscle modeling with the focus on Hill-type muscle models. GAMM-Mitteilungen.

[CR70] Scholz JP, Schöner G (1999). The uncontrolled manifold concept: identifying control variables for a functional task. Exp Brain Res.

[CR71] Shampine L, Gordon M (1975). Computer solution of ordinary differential equations: the initial value problem.

[CR72] Sherman MA, Seth A, Delp SL (2013) What is a moment arm? Calculating muscle effectiveness in biomechanical models using generalized coordinates. In: 9th international conference on multibody systems, nonlinear dynamics, and control, vol 7B. pp DETC2013–13633 10.1115/DETC2013-13633 PMC4404026 25905111

[CR73] Smith JW (1957). The forces operating at the human ankle joint during standing. J Anat.

[CR74] Stanev D, Moustakas K (2019). Stiffness modulation of redundant musculoskeletal systems. J Biomech.

[CR75] Stollenmaier K, Ilg W, Haeufle DFB (2020). Predicting perturbed human arm movements in a neuro-musculoskeletal model to investigate the muscular force response. Front Bioeng Biotechnol.

[CR76] Tarbouriech S, Turner M (2009). Anti-windup design: an overview of some recent advances and open problems. IET Control Theory Appl.

[CR77] Todorov E, Li W, Pan X (2005). From task parameters to motor synergies: a hierarchical framework for approximately optimal control of redundant manipulators. J Robot Syst.

[CR78] Tresch MC, Saltiel P, Bizzi E (1999). The construction of movement by the spinal cord. Nat Neurosci.

[CR79] Tseng Y-W, Diedrichsen J, Krakauer JW, Shadmehr R, Bastian AJ (2007). Sensory prediction errors drive cerebellum-dependent adaptation of reaching. J Neurophysiol.

[CR80] van Soest AJ, Bobbert MF (1993). The contribution of muscle properties in the control of explosive movements. Biol Cybern.

[CR81] Wiener N (1948). Cybernetics, or control and communication in the animal and the machine.

[CR82] Wolpert D, Kawato M (1998). Multiple paired forward and inverse models for motor control. Neural Netw.

[CR83] Wolpert DM (1997). Computational approaches to motor control. Trends Cogn Sci.

[CR84] Wolpert DM, Doya K, Kawato M (2003). A unifying computational framework for motor control and social interaction. Philos Trans R Soc Lond Ser B Biol Sci.

[CR85] Wolpert DM, Flanagan JR (2001). Motor prediction. Curr Biol.

