# Supplementary material for: A geometry- and muscle-based control architecture for synthesising biological movement
Source: Biol Cybern. 2021 Feb 15;115(1):7–37. doi: 10.1007/s00422-020-00856-4 (PMC7925510; doi:10.1007/s00422-020-00856-4)
Supplement: Supplementary file 1 — Supplementary material 1 (pdf 1387 KB) [file 422_2020_856_MOESM1_ESM.pdf]

Supplementary material describing the all-body model allmin

Johannes Walter, Marc Jacob, Katrin Stollenmaier, Patrick Lerge, and Syn Schmitt

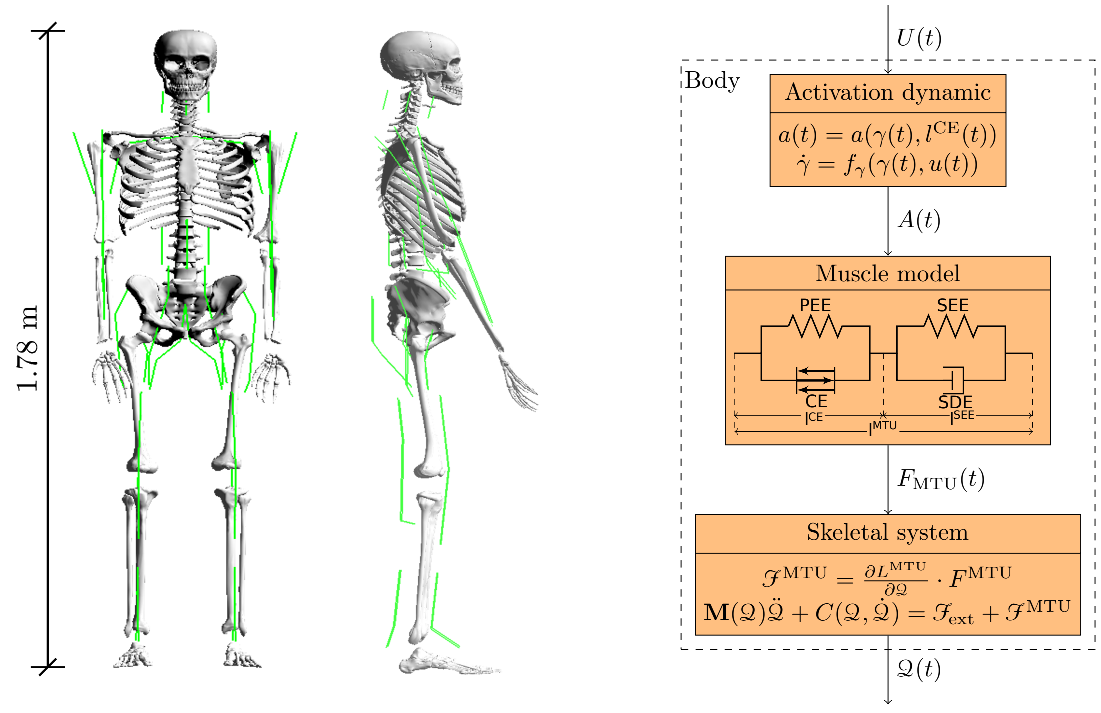

(a) (b)

Figure 1: (a) Frontal and side view of the visualization of the musculoskeletal model of the human body. The green lines show the muscle geometry. (c) Structure of the model: the motor command $U(t) \in \mathbb{R}^{n_{\mathrm{MTU}}}$ is fed into the model of activation dynamics (Hatze, 1977; Rockenfeller and Günther, 2018) of muscles which relates the neuronal stimulation to muscular activity $A(t) \in \mathbb{R}^{n_{\mathrm{MTU}}}$ that drives the muscle model (Haeufle et al., 2014). The muscles produce forces $F^{\mathrm{MTU}}(t) \in \mathbb{R}^{n_{\mathrm{MTU}}}$ that act on the rigid bodies of the skeletal system. The resultant joint torques $\mathcal{T}^{\mathrm{MTU}}$ depend on the respective moment arms $\frac{\partial L^{\mathrm{MTU}}}{\partial \mathcal{Q}}$. In combination with external forces, this results in a movement of the DoFs $\mathcal{Q}(t) \in \mathbb{R}^{n_{\mathrm{DoF}}}$ of the body.

The musculoskeletal model *allmin* consists of $n_{\mathrm{RGB}} = 15$ rigid bodies (see Table 1). The rigid bodies are connected via 14 joints (see Table 2) including $n_{\mathrm{DoF}} = 20$ degrees of freedom. Each Degree of Freedom (DoF) (except for the wrist) is controlled by an Agonistic-Antagonistic Setup (AAS) beeing congruent with the Elementary Biological Drive (EBD) as described by Schmitt et al. (2019). The musculoskeletal model is actuated by $n_{\mathrm{MTU}} = 36$ Muscle Tendon Units (MTU) (see Table 4 and Figure 1a for first impression).

The model is implemented in C/C++ code within our in-house multi-body simulation code *demoa*.

1 The Multibody System

The skeletal system is modeled as a chain of rigid bodies, connected by rotational joints and described by differential equations. The resulting DoFs $\mathcal{Q}(t) = [q_1(t), \ldots, q_{n_{\text{DoF}}}(t)]^T \in \mathbb{R}^{n_{\text{DoF}}}$ of these rotational joints describe the movement of the rigid bodies over time and are referred to as generalized coordinates. For the equations of motion, a Lagrangian formulation with the generalized coordinates $\mathcal{Q}(t)$ as state variables is realized, which can be set up algorithmically, e.g. as described by Legnani et al. (1996). The evaluation of this algorithm leads to the differential equation of motion of the rigid body system in the form

$$\mathbf{M}(\mathcal{Q})\ddot{\mathcal{Q}} + C(\mathcal{Q}, \dot{\mathcal{Q}}) = \mathcal{F}, \tag{1}$$

where $\mathbf{M} \in \mathbb{R}^{n_{\text{DoF}} \times n_{\text{DoF}}}$ is the mass matrix, $C \in \mathbb{R}^{n_{\text{DoF}}}$ is a vector of gravitational, centrifugal and Coriolis forces and $\mathcal{F} \in \mathbb{R}^{n_{\text{DoF}}}$ is a vector of forces (internal and external) acting on the mechanical part of the system. Hereby $\mathcal{F}$ includes forces, e.g. due to contact of the body to the environment (external), as well as forces of the biological structures, such as muscles, joint limitations (internal).

2 Joint limitations

The joint limitations are modeled as linear one-sided spring-damper elements, acting directly on the respective DoF:

$$f_i^{\text{lmt}} = \begin{cases} k_l(q_i - q_{l,i}) + d_l\dot{q}_i, & q_i < q_{l,i} \\ 0, & q_{l,i} \leq q_i \leq q_{u,i} \\ k_u(q_i - q_{u,i}) + d_u\dot{q}_i, & q_i > q_{u,i} \end{cases} \tag{2}$$

with the lower and upper threshold angles $q_{l/u}$, corresponding to the respective Range of Motion (RoM) (Table 2), and linear spring and damping parameters $k_{l/u} = 100\,[\frac{\text{Nm}}{\text{rad}}]$ and $d_{l/u} = 0.001\,[\frac{\text{Nm}\cdot\text{s}}{\text{rad}}]$. For the joints of the lumbar and cervical spine, as well as the wrist, the same force law is used to model passive properties with different parameters. The upper and lower threshold angles are set to $q_{l/u} = 0$ and the spring and damping parameters are set to $k_{cs} = 10\,[\frac{\text{Nm}}{\text{rad}}]$, $d_{cs} = 0.2\,[\frac{\text{Nm}\cdot\text{s}}{\text{rad}}]$, $k_{ls} = 20\,[\frac{\text{Nm}}{\text{rad}}]$, $d_{ls} = 0.2\,[\frac{\text{Nm}\cdot\text{s}}{\text{rad}}]$, $k_{wr} = 15\,[\frac{\text{Nm}}{\text{rad}}]$, $d_{wr} = 1\,[\frac{\text{Nm}\cdot\text{s}}{\text{rad}}]$.

3 Muscles

The muscles are modeled as lumped muscles, i.e. they represent a multitude of anatomical muscles and motor units. A list of all included muscle elements can be found in Table 4.
The MTU structure is modeled using an extended Hill-type muscle model as described in Haeufle et al. (2014) with muscle activation dynamics as introduced by Hatze (1977) and simplified by Rockenfeller and Günther (2018). Herein, the muscles are activated using a 1st order differential equation of normalized calcium ion concentration (Rockenfeller et al., 2014)

$$\dot{\gamma}(t) = M_{\text{H}}(u(t) - \gamma(t)) \tag{3}$$

and a nonlinear mapping onto the muscles activation

$$a(t) = \frac{a_0 + \varpi}{1 + \varpi}, \tag{4}$$

with $\varpi(\gamma(t), l^{\mathrm{CE}}(t)) = (\gamma(t) \cdot \rho(l^{\mathrm{CE}}))^{\nu}$ and $\rho(l^{\mathrm{CE}}) = \varpi_{\mathrm{opt}} \cdot \frac{l^{\mathrm{CE}}}{l_{\mathrm{opt}}} = \gamma_{\mathrm{c}} \cdot \rho_0 \cdot \frac{l^{\mathrm{CE}}}{l_{\mathrm{opt}}}$. The parameter values are chosen muscle non specifically and are given in Table 5.

The muscle model is a macroscopic model consisting of four elements: the Contractile Element (CE), the Parallel Elastic Element (PEE), the Serial Elastic Element (SEE) and Serial Damping Element (SDE), as illustrated in Figure 1b. Herein, the muscle fibers and their contraction dynamics are described by a contractile element (CE) representing the cross-bridge-cycle of the myosin heads and a parallel elastic element (PEE) representing the passive connective tissue in the muscle belly. The viscoelastic properties of tendons are approximated using a series elastic element (SEE) and a serial damping element (SDE).

The inputs to the muscle model are the length of the MTU l^{MTU}, the contraction velocity of the MTU $\dot{l}^{\mathrm{mtu}}$ and the muscular activity a. The output of the muscle model is a one-dimensional muscle force f^{MTU}. This force drives the movement of the skeletal system.

For the routing of the muscle path around the joints, deflection ellipses are implemented as described by Hammer et al. (2019). The muscle path can move within these ellipses and is deflected as soon as it touches the boundary. For the investigations presented here, we set the length of both half-axes of all ellipses to zero, resulting in fixed via points. The position of these points can be found in Table 3. The resulting moment arms translate the muscle force F^{MTU} to generalized forces $\mathcal{F}^{\mathrm{MTU}}$ acting on the DoFs of the system

$$\mathcal{F}^{\mathrm{MTU}} = \frac{\partial L^{\mathrm{MTU}}}{\partial \mathcal{Q}} \cdot F^{\mathrm{MTU}}. \tag{5}$$

All in all, the governing model dependencies for all muscles $i = 1, ..., n$ are:

$$\begin{aligned}
\dot{l}_i^{\mathrm{CE}} &= f^{\mathrm{CE}}(l_i^{\mathrm{CE}}, l_i^{\mathrm{MTU}}, \dot{l}_i^{\mathrm{MTU}}, a_i) && (6)\\
\dot{a}_i &= f^{a}(a_i, u_i, l_i^{\mathrm{CE}}) && (7)\\
f_i^{\mathrm{MTU}} &= f_i^{\mathrm{MTU}}(l_i^{\mathrm{MTU}}, \dot{l}_i^{\mathrm{MTU}}, l_i^{\mathrm{CE}}, a_i) && (8)\\
\ddot{\mathcal{Q}} &= f^{\mathcal{Q}}(\dot{\mathcal{Q}}, \mathcal{Q}, F^{\mathrm{MTU}}, \mathcal{F}^{\mathrm{lmt}}, \mathcal{F}^{\mathrm{ext}}), && (9)
\end{aligned}$$

where $\mathcal{Q} = \{q_i\}_{i=1}^{n_{\mathrm{DoF}}}$ denotes a generalized state vector that contains all joint angles and $F^{\mathrm{MTU}} = \left\{f_i^{\mathrm{MTU}}\right\}_{i=1}^{n}$, $\mathcal{F}^{\mathrm{lmt,i}} = \left\{f_i^{\mathrm{lmt}}\right\}_{i=1}^{n}$ and $\mathcal{F}^{\mathrm{ext}} = \{f_i^{\mathrm{ext}}\}_{i=1}^{n}$ contain the muscle forces, the joint limitation forces and the external forces, respectively.

4 Model parameters

Body Name	m [kg]	r_x [m]	r_y [m]	h_z [m]	$\mathbf{d}_1$ [m]	Child	$\mathbf{d}_2$ [m]
Pelvis (world)	10.2516	0.1224	0.1643	0.18783	[0,0,0]	Spine	[0.000557293, 0.0000, 0.12213]
						Thigh (l/r)	[0.0147, $\pm$0.0796, $-$0.0657]
Spine	33.2397	0.1224	0.1643	0.4166	[-0.00055, 0.0000, -0.2083]	Head	[0.00055, 0.0000, 0.2083]
						Uparm (l/r)	[0.00677703, $\pm$0.1816, 0.10507988]
Head	4.8869	0.0993	0.0778	0.278194	[-0.0092, 0.0000, -0.11]	-	-
Uparm (l/r)	2.1631	0.0495	–	0.3065	[0.0000, 0.0000, 0.1456]	Forearm (l/r)	[0.0000, 0.0000, -0.1609]
Forearm (l/r)	1.3389	0.0477	–	0.2725	[0.0000, 0.0000, 0.1117]	Hand (l/r)	[0.0000, 0.0000, -0.1608]
Hand (l/r)	0.5252	0.028	0.089	0.192	[0.0000, 0.0000, 0.0574]	-	-
Thigh (l/r)	8.1719	0.0947	–	0.4347	[0.0000, $\mp$0.0188, 0.1782]	Shank (l/r)	[0.0000, 0.0000, -0.2565]
Shank (l/r)	3.3541	0.0597	–	0.4239	[0.0000, $\mp$0.0059, 0.1865]	Foot (l/r)	[0.0000, 0.0000, -0.2374]
Foot (l/r) $*$	1.0172	0.0398	–	0.272	[-0.0656, 0.0000, 0.0402]	-	-

Table 1: List of all bodies included in the model with their mechanical properties with m: mass, r_x,r_y: radius in x and y direction, h_z: height in z direction, $\mathbf{d}_1$: distance proximal joint to the body's center of mass and $\mathbf{d}_2$: distance center of mass to distal joint. The spine body has an underlying curvature based on Kitazaki and Griffin (1997). The allover body dimensons are based on data describing a 50th percentile male from NASA (1978).

Name	Type	Movement	RoM [°]
Lumbar spine	Universal	left/right	$[-30 \dots 30]$
Lumbar spine	Universal	flexion/extension	$[0 \dots 30]$
Cervival spine	Universal	left/right	$[-30 \dots 30]$
Cervival spine	Universal	flexion/extension	$[-30 \dots 30]$
Shoulder (Right)	Universal	abduction/adduction	$[-10 \dots 60]$
Shoulder (Right)	Universal	flexion/extension	$[-100 \dots 10]$
Ellbow (Right)	Revolute	flexion/extension	$[-120 \dots 10]$
Wrist (Right)	Revolute	flexion/extension	$[0 \dots 0]$
Shoulder (Left)	Universal	abduction/adduction	$[-10 \dots 60]$
Shoulder (Left)	Universal	flexion/extension	$[-100 \dots 10]$
Ellbow (Left)	Revolute	flexion/extension	$[-120 \dots 10]$
Wrist (Left)	Revolute	flexion/extension	$[0 \dots 0]$
Hip (Right)	Universal	flexion/extension	$[-120 \cdots -10]$
Hip (Right)	Universal	abduction/adduction	$[-10 \dots 70]$
Knee (Right)	Revolute	flexion/extension	$[-1 \dots 120]$
Ankle (Right)	Revolute	flexion/extension	$[-20 \dots 40]$
Hip (Left)	Universal	flexion/extension	$[-120 \dots 10]$
Hip (Left)	Universal	abduction/adduction	$[-10 \dots 70]$
Knee (Left)	Revolute	flexion/extension	$[-1 \dots 120]$
Ankle (Left)	Revolute	flexion/extension	$[-20 \dots 40]$

Table 2: List of all joints included in the model.

Name	R_O [m]			Parent	R_{DF1} [m]			Parent	R_{DF2} [m]			Parent	R_I [m]			Parent
LSE	−0.0283	0.0000	0.1082	Pelvis	−0.0399	0.0000	0.1101	Pelvis	−0.0416	0.0000	−0.1310	Spine	−0.0315	0.0000	−0.1288	Spine
LSF	0.0181	0.0000	0.1006	Pelvis	0.0878	0.0000	0.0891	Pelvis	0.0693	0.0000	−0.1059	Spine	0.0089	0.0000	−0.1196	Spine
LSSBL	−0.0051	0.0500	0.1044	Pelvis	−0.0051	0.0500	0.1044	Pelvis	−0.0113	0.0500	−0.1242	Spine	−0.0113	0.0500	−0.1242	Spine
LSSBR	−0.0051	−0.0500	0.1044	Pelvis	−0.0051	−0.0500	0.1044	Pelvis	−0.0113	−0.0500	−0.1242	Spine	−0.0113	−0.0500	−0.1242	Spine
CSE	−0.0544	0.0000	0.1990	Spine	−0.0544	0.0000	0.1990	Spine	−0.0560	0.0000	−0.0701	Head	−0.0560	0.0000	−0.0701	Head
CSF	0.0427	0.0000	0.1752	Spine	0.0427	0.0000	0.1752	Spine	0.0436	0.0000	−0.0796	Head	0.0436	0.0000	−0.0796	Head
CSSBL	−0.0059	0.0500	0.1871	Spine	−0.0059	0.0500	0.1871	Spine	−0.0062	0.0500	−0.0748	Head	−0.0062	0.0500	−0.0748	Head
CSSBR	−0.0059	−0.0500	0.1871	Spine	−0.0059	−0.0500	0.1871	Spine	−0.0062	−0.0500	−0.0748	Head	−0.0062	−0.0500	−0.0748	Head
HE (l/r)	−0.0750	±0.0796	0.0253	Pelvis	−0.0750	±0.0896	−0.0947	Pelvis	−0.0750	∓0.0188	0.1209	Thigh	−0.0200	∓0.0088	0.0309	Thigh
HF (l/r)	0.0650	±0.0396	0.1008	Pelvis	0.0750	±0.0396	0.0208	Pelvis	0.0150	∓0.0188	0.1011	Thigh	0.0150	∓0.0188	0.0200	Thigh
HAbd (l/r)	−0.0250	±0.1200	0.0500	Pelvis	0.0000	±0.1519	−0.0300	Pelvis	−0.0300	±0.0400	0.0354	Thigh	−0.0200	±0.0300	0.0050	Thigh
HAdd (l/r)	0.0000	0.0000	0.0000	Pelvis	−0.0100	±0.0100	−0.1000	Pelvis	−0.0050	∓0.0351	0.0902	Thigh	0.0000	∓0.0201	0.0102	Thigh
KF (l/r)	−0.0500	0.0000	0.0000	Thigh	−0.0500	0.0000	−0.1075	Thigh	−0.0594	0.0000	0.1060	Shank	−0.0297	0.0000	0.1000	Shank
KE (l/r)	0.0400	0.0000	0.0000	Thigh	0.0299	0.0000	0.2527	Thigh	0.0300	0.0000	0.0500	Shank	0.0300	0.0000	0.0500	Shank
FE (l/r)	−0.0500	0.0000	−0.0250	Shank	−0.0500	0.0000	−0.1750	Shank	−0.1250	0.0000	0.0500	Foot	−0.1250	0.0000	0.0500	Foot
FF (l/r)	0.0300	0.0000	−0.0250	Shank	0.0300	0.0000	−0.1750	Shank	0.0300	0.0000	0.0500	Foot	0.0300	0.0000	0.0500	Foot
SE (l/r)	−0.0688	±0.1816	0.1128	Spine	−0.0500	0.0000	0.1250	Uparm	−0.0172	0.0000	0.0000	Uparm	−0.0172	0.0000	0.0000	Uparm
SF (l/r)	0.0216	±0.1816	0.1387	Spine	0.0216	±0.1816	0.1387	Spine	0.0172	0.0000	0.0000	Uparm	0.0172	0.0000	0.0000	Uparm
SAbd (l/r)	−0.0263	±0.2422	0.1353	Spine	−0.0263	±0.2422	0.1353	Spine	0.0000	±0.0172	0.0000	Uparm	0.0000	±0.0172	0.0000	Uparm
SAdd (l/r)	−0.0236	0.0000	0.1257	Spine	0.0073	±0.1250	0.1033	Spine	0.0000	∓0.0400	0.1250	Uparm	0.0000	∓0.0172	0.0000	Uparm
EF (l/r)	0.0246	0.0000	0.0000	Uparm	0.0300	0.0000	−0.0500	Uparm	0.0300	0.0000	0.0136	Forearm	0.0238	0.0000	−0.1000	Forearm
EE (l/r)	−0.0246	0.0000	0.0000	Uparm	−0.0493	0.0000	−0.1603	Uparm	−0.0476	0.0000	0.1002	Forearm	−0.0238	0.0000	0.0000	Forearm

Table 3: Muscle routing parameters: Origin R_O, Deflection Point 1 R_{DF1} and 2 R_{DF2} and Insertion R_I relative to their parent body. All numbers in this table are rounded to four decimal digits. Muscle names: EF, EE, FF, FE, HAbd, HAdd, HF, HE, CSF, CSSBL, CSSBR, CSE, KF, KE, LSF, LSSBL, LSSBR, LSE, SAbd, SAdd, SF, SE.

	$F^{\max}$ [N]	$l^{\mathrm{CE,opt}}$ [m]	ΔW^{asc}	$l^{\mathrm{SEE,0}}$ [m]
EF	1420.0	0.1885	1.0	0.1845
EE	1550.0	0.171	0.525	0.18
FF	3000.0	0.15	1.0	0.133
FE	3000.0	0.13	1.0	0.115
HAbd	2000.0	0.18	1.0	0.121
HAdd	2000.0	0.204	0.75	0.136
HF	5000.0	0.195	1.0	0.135
HE	5000.0	0.192	1.0	0.191
CSF	5000.0	0.07	1.5	0.01
CSSBL	5000.0	0.05	1.5	0.01
CSSBR	5000.0	0.046	1.5	0.01
CSE	5000.0	0.062	1.5	0.01
KF	6000.0	0.258	0.525	0.112
KE	6000.0	0.264	1.0	0.28
LSF	15000.0	0.2	1.5	0.11
LSSBL	15000.0	0.09	1.5	0.02
LSSBR	15000.0	0.09	1.5	0.02
LSE	15000.0	0.075	1.5	0.04
SAbd	6000.0	0.12	1.0	0.08
SAdd	6000.0	0.225	1.0	0.12
SF	10000.0	0.1	1.0	0.073
SE	6000.0	0.165	1.0	0.105

Table 4: Muscle-specific actuation parameters, with $F^{\max}$: maximum isometric force, $l^{\mathrm{CE,opt}}$: optimal length of the CE, ΔW^{asc}: width of normalized bell curve in ascending branch of the force-length relationship, $l^{\mathrm{SEE,0}}$ rest length of the SEE, $l^{\mathrm{CE,init}}$: initial length of the CE. Muscle names: Elbow Flexion (EF), Elbow Extension (EE), Foot Flexion (FF), Foot Extension (FE), Hip Abduction (HAbd), Hip Adduction (HAdd), Hip Flexion (HF), Hip Extension (HE), Cervical Spine Flexion (CSF), Cervical Spine Side Bend Left (CSSBL), Cervical Spine Side Bend Right (CSSBR), Cervical Spine Extension (CSE), Knee Flexion (KF), Knee Extension (KE), Lumbar Spine Flexion (LSF), Lumbar Spine Side Bend Left (LSSBL), Lumbar Spine Side Bend Right (LSSBR), Lumbar Spine Extension (LSE), Shoulder Abduction (SAbd), Shoulder Adduction (SAdd), Shoulder Flexion (SF), Shoulder Extension (SE).

	Parameter	Unit	Value	Source	Description
CE	ΔW^{des}	[ ]	0.45	similar to Bayer et al. (2017); Kistemaker et al. (2006)	width of normalized bell curve in descending branch, adapted to match observed force-length curves
	$\nu^{\text{CE,des}}$	[ ]	1.5	Mörl et al. (2012)	exponent for descending branch
	$\nu^{\text{CE,asc}}$	[ ]	3.0	Mörl et al. (2012)	exponent for ascending branch
	$A^{\text{rel},0}$	[ ]	0.2	Günther (1997)	parameter for contraction dynamics: maximum value of A^{rel}
	$B^{\text{rel},0}$	[1/s]	2.0	Günther (1997)	parameter for contraction dynamics: maximum value of B^{rel}
	$\mathcal{S}^{\text{ecc}}$	[ ]	2.0	van Soest et al. (1993)	relation between $F(v)$ slopes at $v^{\text{CE}} = 0$
	$\mathcal{F}^{\text{ecc}}$	[ ]	1.5	van Soest et al. (1993)	factor by which the force can exceed F^{isom} for large eccentric velocities
PEE	$\mathcal{L}^{\text{PEE},0}$	[ ]	0.95	Günther (1997)	rest length of PEE normalized to optimal length of CE
	ν^{PEE}	[ ]	2.5	Mörl et al. (2012)	exponent of F^{PEE}
	$\mathcal{F}^{\text{PEE}}$	[ ]	2.0	Mörl et al. (2012)	force of PEE if l^{CE} is stretched to ΔW^{des}
SDE	D^{SDE}	[ ]	0.3	Mörl et al. (2012)	dimensionless factor to scale $d^{\text{SDE,max}}$
	R^{SDE}	[ ]	0.01	Mörl et al. (2012)	minimum value of d^{SDE} (at $F^{MTU} = 0$), normalized to $d^{\text{SDE,max}}$
SEE	$\Delta U^{\text{SEE,nll}}$	[ ]	0.0425	Mörl et al. (2012)	relative stretch at non-linear linear transition
	$\Delta U^{\text{SEE,l}}$	[ ]	0.017	Mörl et al. (2012)	relative additional stretch in the linear part providing a force increase of $\Delta F^{\text{SEE},0}$
	$\Delta F^{\text{SEE},0}$	[N]	$0.4\,F^{\text{max}}$		both force at the transition and force increase in the linear part
activation dynamics	M_H	[1/s]	11.3	Kistemaker et al. (2006)	time constant for the activation dynamics
	γ_c	[mol/l]	1.37e-4	Kistemaker et al. (2006)	constant for the activation dynamics
	ρ_0	[l/mol]	5.27e4	Kistemaker et al. (2006)	constant for the activation dynamics
	a_0	[ ]	0.005	Günther (1997)	resting active state for all activated muscle fibers
	ν	[ ]	3	Kistemaker et al. (2006)	constant for the activation dynamics

Table 5: Muscle non-specific actuation parameters for the muscles and the activation dynamics.

List of Abbreviations

AAS Agonistic-Antagonistic Setup

MTU Muscle Tendon Unit

DHM Digital Human Model

CE Contractile Element

PEE Parallel Elastic Element

SEE Serial Elastic Element

SDE Serial Damping Element

EBD Elementary Biological Drive

DoF Degree of Freedom

RoM Range of Motion

EF Elbow Flexion

EE Elbow Extension

FF Foot Flexion

FE Foot Extension

HAbd Hip Abduction

HAdd Hip Adduction

HF Hip Flexion

HE Hip Extension

CSF Cervical Spine Flexion

CSSBL Cervical Spine Side Bend Left

CSSBR Cervical Spine Side Bend Right

CSE Cervical Spine Extension

KF Knee Flexion

KE Knee Extension

LSF Lumbar Spine Flexion

LSSBL Lumbar Spine Side Bend Left

LSSBR Lumbar Spine Side Bend Right

LSE Lumbar Spine Extension

SAbd Shoulder Abduction

SAdd Shoulder Adduction

SF Shoulder Flexion

SE Shoulder Extension

References

Bayer, A., Schmitt, S., Günther, M., and Haeufle, D. F. The influence of biophysical muscle properties on simulating fast human arm movements. *Computer Methods in Biomechanics and Biomedical Engineering*, 20(8):803–821, 2017.

Günther, M. *Computersimulationen zur Synthetisierung des muskulär erzeugten menschlichen Gehens unter Verwendung eines biomechanischen Mehrkörpermodells.* PhD thesis, Eberhard-Karls-Universität zu Tübingen, 1997.

Haeufle, D. F. B., Günther, M., Bayer, A., and Schmitt, S. Hill-type muscle model with serial damping and eccentric force-velocity relation. *Journal of Biomechanics*, 47(6):1531–1536, 2014.

Hammer, M., Günther, M., Haeufle, D., and Schmitt, S. Tailoring anatomical muscle paths: a sheath-like solution for muscle routing in musculoskeletal computer models. *Mathematical Biosciences*, accepted (February):68–81, 2019.

Hatze, H. A Myocybernetic Control Model of Skeletal Muscle. *Biol. Cybernetics*, 25:103–119, 1977.

Kistemaker, D. a., Van Soest, A. J., and Bobbert, M. F. Is equilibrium point control feasible for fast goal-directed single-joint movements? *Journal of Neurophysiology*, 95(5):2898–912, 2006.

Kitazaki, S. and Griffin, M. A modal analysis of whole-body vertical vibration, using a finite element model of the human body. *Journal of Sound and Vibration*, 200(1):83 – 103, 1997. doi: https://doi.org/10.1006/jsvi.1996.0674.

Legnani, G., Casolo, F., Righettini, P., and Zappa, B. A homogeneous matrix approach to 3d kinematics and dynamics - i. theory. *Mechanism and Machine Theory*, 31(5):573 – 587, 1996.

Mörl, F., Siebert, T., Schmitt, S., Blickhan, R., and Günther, M. Electro-Mechanical Delay in Hill-Type Muscle Models. *Journal of Mechanics in Medicine and Biology*, 12(05):1250085, 2012.

NASA. *Anthropometric Source Book, Volume I-III.* NASA Anthropometry Project, 1978.

Rockenfeller, R. and Günther, M. Inter-filament spacing mediates calcium binding to troponin: A simple geometric-mechanistic model explains the shift of force-length maxima with muscle activation. *Journal of Theoretical Biology*, 454:240 – 252, 2018.

Rockenfeller, R., Günther, M., Schmitt, S., and Götz, T. Comparing different muscle activation dynamics using sensitivity analysis. *CoRR*, 2014.

Schmitt, S., Günther, M., and Haeufle, D. F. B. The dynamics of the skeletal muscle: a systems biophysics perspective on muscle modeling with the focus on hill-type muscle models. *GAMM-Mitteilungen*, 42(3):nil, 2019.

van Soest, A., Bobbert, M., Iijima, T., Shimizu, K., and Asanuma, N. The Contribution of Muscle Properise in the Control of Explosive Movments. *Biological Cybernetics*, 69(3):195–204, 1993.
